# Carboxylations of (Hetero)Aromatic C–H Bonds Using an Alkyl Silyl Carbonate Reagent

**DOI:** 10.1021/acs.orglett.4c04388

**Published:** 2024-12-20

**Authors:** Kanta Shimotai, Ozora Sasamoto, Masanori Shigeno

**Affiliations:** † Department of Biophysical Chemistry, Graduate School of Pharmaceutical Science, Tohoku University, AobaSendai 980-8578, Japan; ‡ JST, PRESTO, KawaguchiSaitama332-0012, Japan

## Abstract

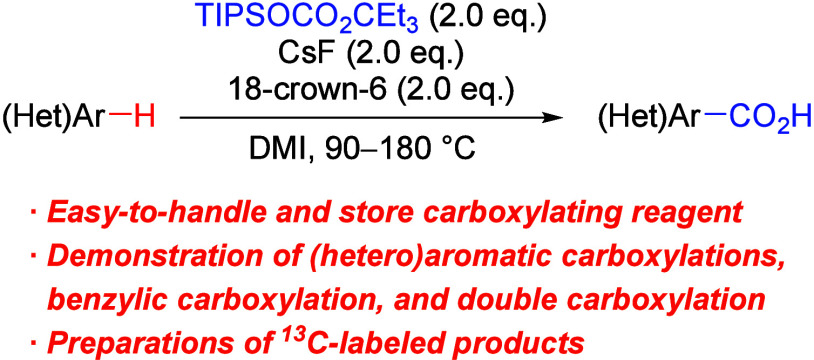

In this paper, we report that the use of an alkyl silyl carbonate reagent combined with CsF and 18-crown-6 facilitates efficient direct carboxylations of (hetero)aromatic C–H bonds. This system also enables benzylic carboxylation of a toluene derivative and double carboxylation of methyl heteroarene. The carbonate reagent is characterized by its ease of handling and storage. Moreover, we demonstrate the application of this system in ^13^C-labeling experiments.

Direct carboxylations of (hetero)aromatic C–H bonds using CO_2_ have been extensively investigated over the past decade, particularly because its abundance, low cost, and low toxicity make CO_2_ an ideal C1 source.^1^ The resulting (hetero)aromatic carboxylic acid derivatives are valuable compounds with potent biologically activities.^2^ Existing carboxylation protocols encompass transition-metal catalysis,^1a−1g^ Lewis-acid mediated reactions,^1e,1f^ and photocatalysis.^1g,1h^ Additionally, significant advancements have been made using Brønsted bases, which utilize a distinct reaction pathway involving the deprotonation of the C–H bond to form a carbanion species, followed by nucleophilic addition to CO_2_.^1e,1f,3,4^ This approach offers complementary advantages in terms of substrate scope and functional group tolerance. Our group has contributed to this field, demonstrating that a combined Brønsted base system (e.g., LiO-*t*-Bu/CsF/18-crown-6) is highly effective for such carboxylations (Figure 1A).^5^ For instance, this system facilitates reactions with electron-rich heteroarenes, such as benzothiophene and benzofuran, which have p*K*_a_ values exceeding 32,^5a,5c^ and with aromatic compounds that contain electron-withdrawing substituents.^5d^ It also promotes benzylic carboxylations of toluenes^5e^ and double carboxylations of methyl heteroarenes.^5b,5f^

**Figure 1:**
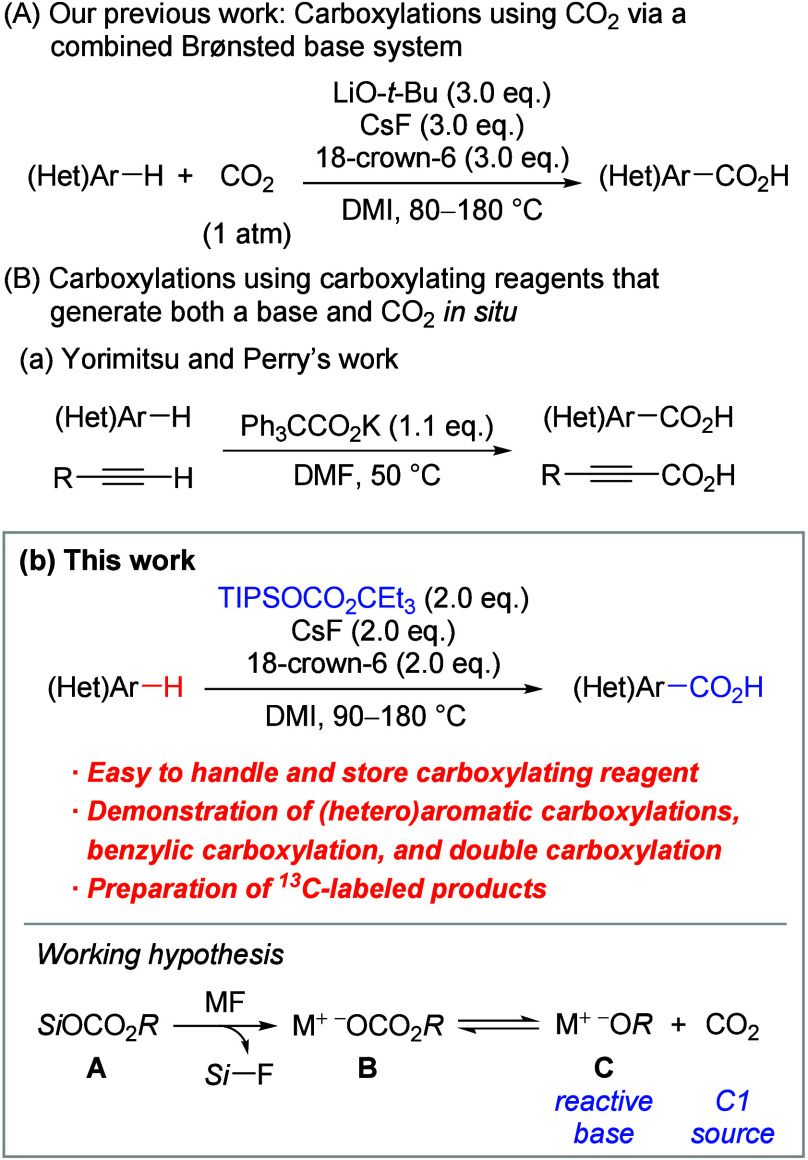
(A) Carboxylations using CO_2_ via a combined Brønsted base system (our previous work) and (B) carboxylations using carboxylating reagents that generate both a base and CO_2_*in situ*.

Along with the progress in carboxylations using CO_2_, there has been increasing focus on developing reactions with carboxylating reagents that generate a base and CO_2_*in situ* (Figure 1B).^6−8^ This approach simplifies experimental operations by eliminating the need for gaseous CO_2_, thereby making it suitable for the library synthesis of carboxylic acid derivatives. Additionally, the liquid or solid state of the carboxylating reagent enables easy and precise weighing. This is particularly advantageous for carbon labeling experiments, given the high costs of ^13^CO_2_ and ^14^CO_2_. Yorimitsu and Perry’s group employed potassium triphenylacetate (Ph_3_CCO_2_K), which generates the base Ph_3_CK (p*K*_a_ of Ph_3_CH, 30.6, DMSO) *in situ*,^9^ to demonstrate its efficacy in carboxylating (hetero)arenes and alkynes, particularly targeting substrates with p*K*_a_ values below 30 (Figure 1B-a).^6a^ They also observed that benzothiophene, with a p*K*_a_ of 32, underwent the reaction under heated conditions at 160 °C, whereas benzofuran, with a p*K*_a_ of 33.2, afforded a modest product yield.^10^ In our studies of the combined Brønsted base systems, we found that the lithium carbonate reagent LiOCO_2_-*t*-Bu, when used with CsF and 18-crown-6, serves as an effective carboxylating reagent.^5^ However, this reagent is sensitive to air and moisture, leading to degradation under atmospheric conditions. Therefore, the development of practical reactions that utilize robust carboxylation reagents is crucial and warrants investigation. In this study, we focused on the use of silyl-protected alkyl carbonate reagents (Figure 1B-b, upper scheme). The reagent has previously been employed in the synthesis of silyl-carbamates and -carbonates using amine and alcohol nucleophiles, respectively; however, its application in carboxylation reactions involving C–C bond formation remains unexplored.^11^ We hypothesized that treating alkyl silyl carbonate reagent **A** with a fluoride salt would yield anionic carbonate species **B**, which in turn would generate reactive alkoxide base **C** (e.g., p*K*_a_ of *t*-BuOH: 32.2 in DMSO)^12^ and CO_2_*in situ* for carboxylation (Figure 1B-b, lower scheme). The results of this investigation are reported below.

At the outset of this study, we examined the reaction conditions using benzothiophene (**1a**) as a model substrate (Table 1). Initially, the reaction was conducted with *tert*-butyl trimethylsilyl carbonate (**2a**) in the presence of CsF and 18-crown-6, producing the desired carboxylation product, **3a**, in 32% yield (entry 1). Subsequent experiments with *tert*-butyldimethylsilyl-carbonate (**2b**) and triisopropylsilyl-carbonate (**2c**) afforded slight increases in yields, achieving 34% and 50% yields, respectively (entries 2 and 3). Employing 3-ethylpentan-3-yl triisopropylsilyl carbonate (**2d**) was more effective, delivering the product in 86% yield (entry 4).^13^ Excluding 18-crown-6 from the reaction moderately reduced the product yield (entry 5).^14^ Moreover, increasing the reaction temperature to 160 °C resulted in a product yield of 98% (entry 6). Reducing the amount of each reagent to 1.5 equiv resulted in a slight decrease in efficiency (entry 7); however, a good yield of 81% was still obtained.^15^ This system was scalable to 1.0 mmol, producing the product in 90% yield (entry 8).

**Table 1:**
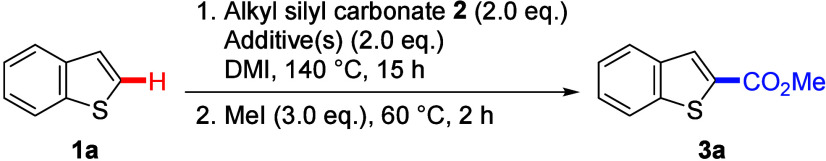
Optimization of Reaction Conditions of **1a**^a^

entry	alkyl silyl carbonate	additive(s)	yield of **3a** (%)^b^
1	Me_3_SiOCO_2_-*t*-Bu (**2a**)	CsF + 18-crown-6	32
2	*t*-BuMe_2_SiOCO_2_-*t*-Bu (**2b**)	CsF + 18-crown-6	34
3	*i*-Pr_3_SiOCO_2_-*t*-Bu (**2c**)	CsF + 18-crown-6	50
4	*i*-Pr_3_SiOCO_2_-CEt_3_ (**2d**)	CsF + 18-crown-6	86
5	**2d**	CsF	75
6	**2d**	CsF + 18-crown-6	98^c^
7	**2d** (1.5 equiv)	CsF + 18-crown-6 (1.5 equiv)	81^c^
8	**2d**	CsF + 18-crown-6	90^d^

a Carboxylation: **1a** (0.20 mmol), alkyl silyl carbonate (0.40 mmol), additive(s) (0.40 mmol), DMI (1.0 mL), 140 °C, 15 h. Methyl esterification: MeI (0.60 mmol), 60 °C, 2 h.

b Isolated yields.

c Reaction was conducted at 160 °C.

d **1a** (1.0 mmol), **2d** (2.0 mmol), CsF (2.0 mmol), 18-crown-6 (2.0 mmol), DMI (5.0 mL), 160 °C, 15 h.

Using the optimized reaction conditions, we explored the scope of heteroarenes (Scheme 1). The carboxylation reactions of benzothiophene derivatives **1b**–**1f**, which bear methyl, methoxy, chloro, bromo, and cyano groups, respectively, proceeded in excellent yields (95–99%). Moreover, 3-methyl-benzothiophene (**1g**) and 3-bromobenzothiophene (**1h**) afforded the products in 72% and 99% yields, respectively. The carboxylation of benzofuran (**1i**) was also completed, resulting in the formation of product **3i** in 74% yield.^16^ Additionally, 3-cyano-indole (**1j**) and 3-formyl-indole (**1k**) were amenable to the reaction, yielding the products in 99% and 82% yields, respectively. Furthermore, this system proved effective for the carboxylations of thiophene derivatives **1l**–**1n** containing phenyl, bromo, and benzoyl moieties, respectively, achieving high yields (85–99%). The furan derivative **1o** produced product **3o**, albeit in a moderate yield of 47%.^17^

**Scheme 1:**
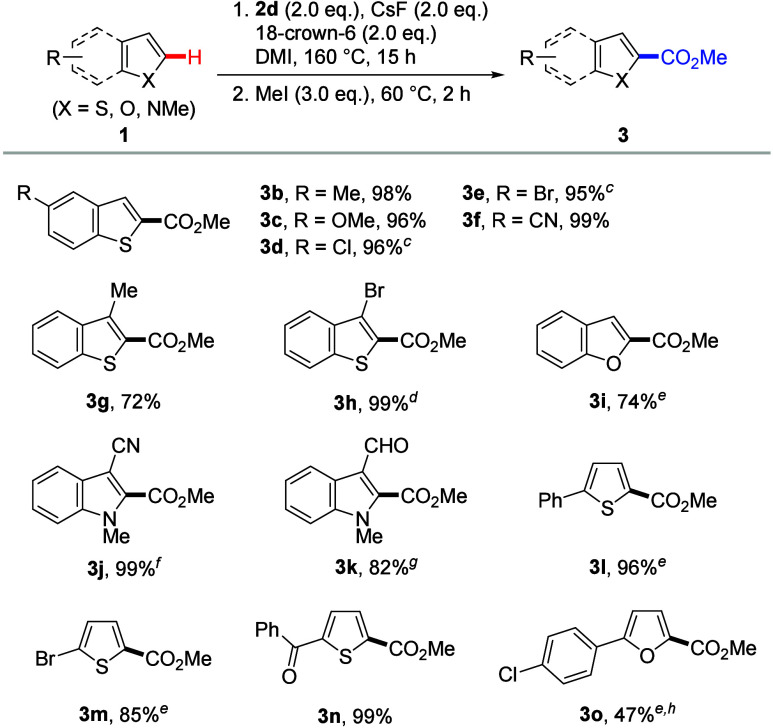
Scope of Heteroarenes, Reactions were conducted on a 0.2 mmol scale. Isolated yields. Reaction was conducted for 3 h. Reaction was conducted for 1 h. Reaction was conducted at 180 °C. Reaction was conducted at 120 °C. Reaction was conducted at 150 °C. **2d** (3.0 equiv), CsF (3.0 equiv), and 18-crown-6 (3.0 equiv) were used.

Subsequently, we explored the scope of arenes (Scheme 2). The carboxylation reactions of 1,3,5-trichlorobenzene (**4a**) and 1,3,5-tribromobenzene (**4b**) occurred preferentially over undesirable side reactions, such as aryne formation,^18a^ halogen dance,^18b^ and single electron transfer involving the elimination of halogen atoms.^18c^ When 3-fluorobenzonitrile (**4c**), 3-chlorobenzonitrile (**4d**), 1,3-dicyanobenzene (**4e**), 3-nitrobenzonitrile (**4f**), and 3-(trifluoromethoxy)benzonitrile (**4g**) were subjected to the reactions, C2-carboxylation products **5c**–**5g** were obtained, respectively, in moderate to good yields. Furthermore, the reactions of 1,2,3-trichlorobenzene (**4h**) and 1,2-dicyanobenzene (**4i**) produced the target products in 76% and 79% yields, respectively.

**Scheme 2:**
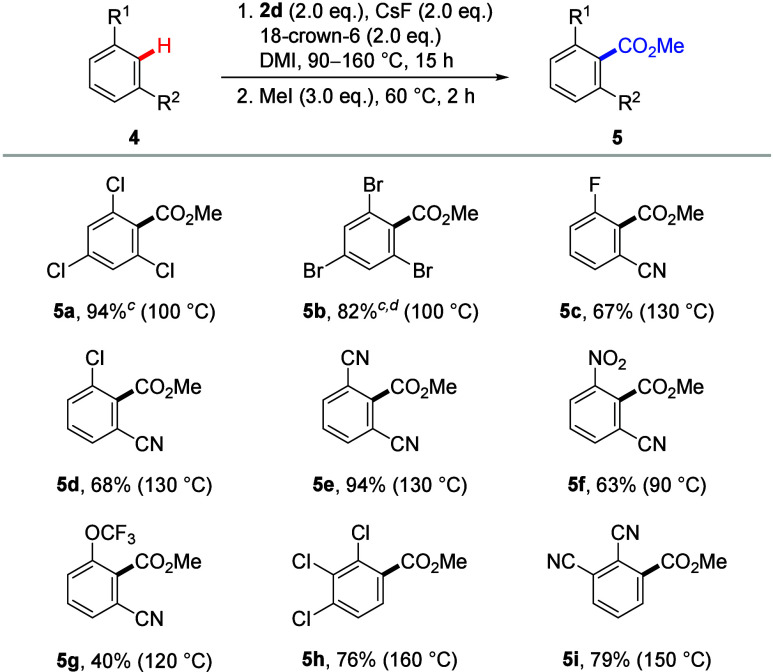
Scope of Arenes, Reactions were conducted on a 0.2 mmol scale. Isolated yields. Reactions were conducted for 5 h. **2d** (3.0 equiv), CsF (3.0 equiv), and 18-crown-6 (3.0 equiv) were used.

The current system was utilized for benzylic carboxylation reactions (Scheme 3). Specifically, treatment of 4-methyl biphenyl (**6**), which has a high p*K*_a_ (38.57, THF),^19^ afforded product **7** in 54% yield.^20^ This achievement is attributed to the acidification of the benzylic C–H bond through the interaction between the Cs^+^ cation and aromatic π system, as reported in our previous studies.^5e,21^ Moreover, double carboxylation of 2-methylbenzothiophene (**8**) at both the benzylic and 3-positions was achieved, forming product **9** in 67% yield.^22^

**Scheme 3:**
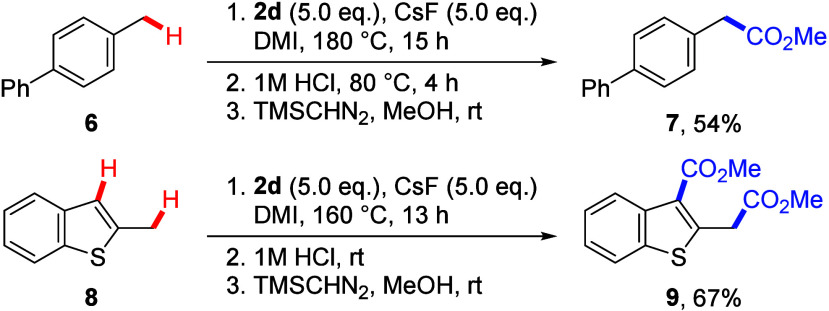
Benzylic Carboxylation of **6** and Double Carboxylation of **8**, Reactions were conducted on a 0.2 mmol scale. Isolated yields.

The stabilities of the carbonate reagents were also investigated (Scheme 4). Storing LiOCO_2_-*t*-Bu in air for 7 days prior to use did not lead to the formation of carboxylation product **3a**. In contrast, alkyl silyl carbonate **2d** stored under the same conditions afforded **3a** in a good yield of 91%, comparable to that obtained with a freshly prepared reagent. These results highlight the robustness of the alkyl silyl carbonate reagent.

**Scheme 4:**
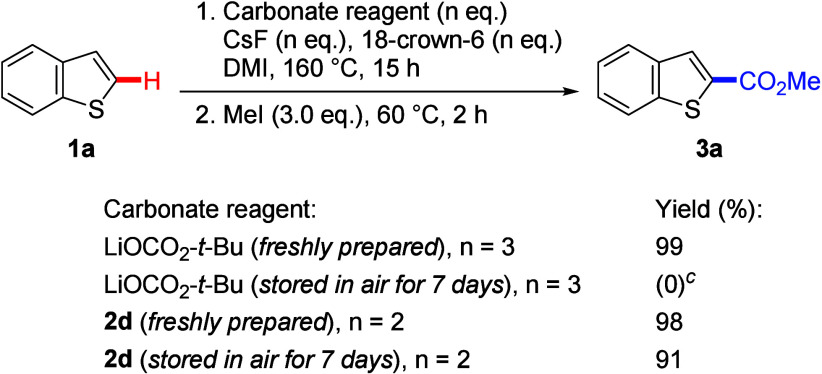
Investigation of the Stability of Carbonate Reagents, Reactions were conducted on a 0.2 mmol scale. Isolated yields. ^1^H NMR yield was determined using 1,1,2-trichloroethane as the internal standard.

We subsequently directed our attention to the application of this system for the preparation of carbon-isotope-labeled products (Scheme 5). Carbon labeling is crucial for elucidating reaction mechanisms and advancing pharmaceutical research.^23^ Treatment of substrate **1a** with carbonate reagent 2d*, derived from ^13^CO_2_, resulted in the formation of ^13^C-labeled product 3a* in 95% yield. Furthermore, this system was demonstrated to facilitate the synthesis of doubly ^13^C-labeled product 9* from substrate **8**.

**Scheme 5:**
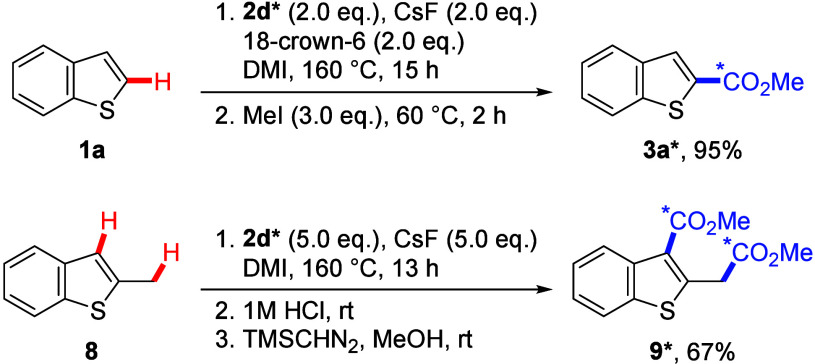
^13^C-Labeling Experiments with ^13^C-Labeled Carbonate Reagent 2d*, Reactions were conducted on a 0.2 mmol scale. Isolated yields.

In summary, we developed carboxylation reactions utilizing an alkyl silyl carbonate reagent. This system facilitates the efficient carboxylations of (hetero)arenes containing a diverse array of functional groups. It also effectively promotes the benzylic carboxylation of toluene derivative and the double carboxylation of methyl heteroarene. Moreover, the silyl carbonate reagent demonstrates superior stability in air compared to its lithium counterpart. These results underscore the practicality of this silyl carbonate reagent. Additionally, we successfully applied this strategy to the preparation of ^13^C-labeled products.

## Data Availability

The data underlying this study are available in the published article and its Supporting Information.

## Supporting Information Available

Effects of additives, effects of solvents, experimental procedures and spectral data for obtained products, and ^1^H, ^13^C, and ^19^F NMR spectra (PDF)
