# Supplementary material for: Carboxylations of (Hetero)Aromatic C–H Bonds Using an Alkyl Silyl Carbonate Reagent
Source: Org Lett. 2024 Dec 20;27(1):352–6. doi: 10.1021/acs.orglett.4c04388 (PMC11731326; doi:10.1021/acs.orglett.4c04388)

Carboxylations of (hetero)aromatic C−H bonds using an alkyl silyl carbonate reagent

Kanta Shimotai,[1] Ozora Sasamoto,[1] and Masanori Shigeno*[1,2]

[1]Department of Biophysical Chemistry, Graduate School of Pharmaceutical Science,

Tohoku University, Aoba, Sendai, 980-8578, Japan

[2]JST, PRESTO, Kawaguchi, Saitama 332-0012, Japan

*E-mail: masanori.shigeno.e5@tohoku.ac.jp

Supporting Information

Table of Contents

General methods......S2
Effects of additives (Table S1)......S3
Effects of solvents (Table S1)......S4
Experimental procedures and spectra data for obtained products......S5-S20
References......S21-S22
^{1}H, ^{13}C, and ^{19}F NMR spectra......S23-S55

General methods. All reactions were carried out under N_2 or Ar atmosphere. Flash column chromatography was performed with Kanto silica gel 60 N (spherical, neutral, 40-50 μm). Preparative thin-layer chromatography was performed with silica gel (Wakogel® B-5F). Melting points (Mp) were determined with a Yazawa micro melting point apparatus without correction. Infrared (IR) data were recorded on SensIR ATR (Attenuated Total Reflectance) FT-IR, and absorbance frequencies are reported in reciprocal centimeters (cm^{-1}). NMR data were recorded on a JEOL AL400 spectrometer (395.75 MHz for ^1H, 99.50 MHz for ^{13}C), a JEOL 400YH spectrometer (100.5 MHz for ^{13}C, 376.1 MHz for ^{19}F), a Varian Mercury (399.17 MHz for ^1H, 100.38 MHz for ^{13}C), or a JEOL ECA600 spectrometer (597.17 MHz for ^1H, 150.907 MHz for ^{13}C). Chemical shifts are expressed in δ (parts per million, ppm) values, and coupling constants are expressed in herts (Hz). ^1H NMR spectra were referenced to tetramethylsilane as an internal standard or to a solvent signal ($CDCl_3$: 7.26 ppm). ^{13}C NMR spectra were referenced to a solvent signal ($CDCl_3$: 77.0 ppm). ^{19}F NMR spectra were referenced to 4-fluorotoluene ($\delta = -118.0$ ppm) as an internal standard. Low and high resolution mass spectra (LRMS and HRMS) were obtained from Mass Spectrometry Resource, Graduate School of Pharmaceutical Sciences, Tohoku University, on a JEOL JMS-DX 303 and JMS-700/JMS-T 100 GC spectrometer, respectively. Gel permeation chromatography (GPC) was conducted with a Recycling Preparative HPLC LC-9210 (Japan Analytical Industry, Co. Ltd.).

Table S1. Effects of additives[a]

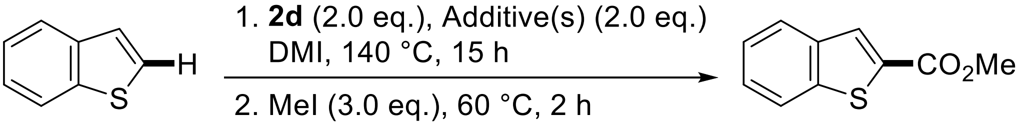

1a **3a**

Entry	Additive(s)	Yield of **3a** (%)[b]
1	LiF + 12-crown-4	0
2	NaF + 15-crown-5	0
3	KF + 18-crown-6	14
4	RbF + 18-crown-6	41
5	CsF + 18-crown-6	(86)[c]
6	18-crown-6	0
7	–	0

[a]**1a** (0.20 mmol), **2d** (0.40 mmol), additive(s) (0.40 mmol), DMI (1.0 mL), 140 °C, 15 h. [b]The yields of **3a** were determined using ^{1}H NMR spectroscopy with 1,1,2-trichloroethane as the internal standard. [c]Yield in parentheses denotes an isolated yield of **3a**.

Table S2. Effects of solvents[a]

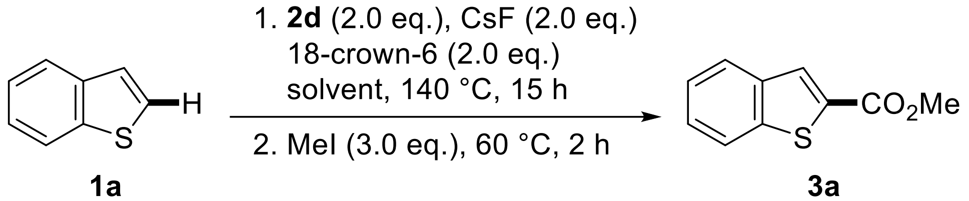

Entry	Solvent	Yield of **3a** (%)[b]
1	DMI	(86)[c]
2	DMF	(86)[c]
3	DMSO	0
4	NMP	0
5	Bu_2O	0

[a]**1a** (0.20 mmol), **2d** (0.40 mmol), CsF (0.40 mmol), 18-crown-6 (0.40 mmol), solvent (1.0 mL), 140 °C, 15 h. [b]The yields of **3a** were determined using ^{1}H NMR spectroscopy with 1,1,2-trichloroethane as the internal standard. [c]Yields in parentheses denote isolated yields of **3a**.

Materials. Unless otherwise noted, materials were purchased from Tokyo Kasei Co., Aldrich Inc., and other commercial suppliers, and were used as received. Anhydrous DMF was purchased from Kanto Chemical Co. **1c**[1], **1d**[1], **1e**[2], **1f**[3], **1j**[4], **1k**[5], **1l**[6], **1o**[7], **2a**[8], **8a**[9], and *t*-$BuOCO_2Li$[10] were prepared according to the literature procedures. **2b**, **2c**, **2d**, and **2d*** were prepared according to the procedure shown below.

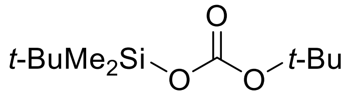

***tert*-Butyl (*tert*-butyldimethylsilyl) carbonate (2b).** In a glove box under Ar atmosphere, LiO-*t*-Bu (804.2 mg, 10.0 mmol) was added to an oven-dried two-neck round flask (50 mL) equipped with a stirrer bar. The flask was sealed with a rubber cap and three-way stopcock, and taken out from the glove box. The flask was evacuated and refilled with CO_2 gas using a CO_2 cylinder. Subsequently, THF (15 mL) was added. After stirring at 0 °C for 1 h, a solution of *tert*-butyldimethylsilyl chloride (1.57 g, 10.4 mmol) in THF (7 mL) was slowly added. The mixture was further stirred at 0 °C for 1 h. Then, after hexane (15 mL) was added, the mixture was filtered (Celite®, hexane) and concentrated. This procedure was repeated two times. The resulting oil was purified by distillation (5 mmHg, 49 °C) to afford **2b** (0.901 g, 3.88 mmol, 39%) as a colorless oil. ^{1}H NMR (400 MHz, $CDCl_3$/TMS) δ 1.47 (s, 9H), 0.95 (s, 9H,), 0.27 (s, 6H). ^{13}C NMR (100 MHz, $CDCl_3$/TMS) δ 151.4, 81.2, 27.7, 25.6, 17.8, -4.70. LRMS (FAB) *m/z* 233 $[M + H]^+$. HRMS (FAB-EB) *m/z*: $[M + H]^+$ Calcd. for $C_{11}H_{25}O_3Si$: 233.1568, found: 233.1571. IR (neat): 2953, 2861, 1756, 1721, 1370, 1294, 1253, 1163, 829 cm^{-1}.

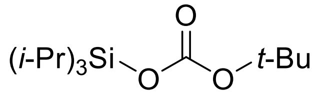

***tert*-Butyl (triisopropylsilyl) carbonate (2c).** In a glove box under Ar atmosphere, LiO-*t*-Bu (801.4 mg, 10.0 mmol) was added to an oven-dried two-neck round flask (50 mL) equipped with a stirrer bar. The flask was sealed with a rubber cap and three-way stopcock, and taken out from the glove box. The flask was evacuated and refilled with CO_2 gas using a CO_2 cylinder. Subsequently, THF (15 mL) was added. After stirring at 0 °C for 1 h, a solution of triisopropylsilyl chloride (1.94 g, 10.1 mmol) in THF (7 mL) was slowly added. The mixture was further stirred at 0 °C for 1 h. Then, after hexane (15 mL) was added, the mixture was filtered (Celite®, hexane) and concentrated. This procedure was repeated two times. The resulting oil was purified by distillation (5 mmHg, 81 °C) to afford **2c** (1.21 g, 4.41 mmol, 44 %) as a colorless oil. ^{1}H NMR (400 MHz, $CDCl_3$/TMS) δ 1.48 (s, 9H), 1.37-1.25 (m, 3H), 1.10 (d, 18H, J = 7.3 Hz). ^{13}C NMR (100 MHz) δ 151.6, 81.0, 27.7, 17.7, 12.0. LRMS (FAB) *m/z* 275 $[M + H]^+$. HRMS (FAB-EB) *m/z*: $[M + H]^+$ Calcd. for $C_{14}H_{31}O_3Si$: 275.2037, found: 275.2037. IR (neat): 2944, 2870, 1719, 1369, 1295, 1162, 884. cm^{-1}.

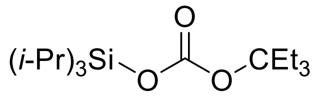

3-Ethylpentan-3-yl (triisopropylsilyl) carbonate (2d). In a glove box under Ar atmosphere, $LiOCEt_3$[11] (4.89 g, 40.0 mmol) was added to an oven-dried two-neck round flask (200 mL) equipped with a stirrer bar. The flask was sealed with a rubber cap and three-way stopcock, and taken out from the glove box. The flask was evacuated and refilled with CO_2 gas with CO_2 gas using a CO_2-filled balloon. Subsequently, THF (40 mL) was added. After stirring at 0 °C for 1 h, a solution of triisopropylsilyl chloride (7.71 g, 40.0 mmol) in THF (20 mL) was slowly added. The mixture was further stirred at 0 °C for 1 h. Then, after hexane (40 mL) was added, the mixture was filtered (Celite®, hexane) and concentrated. This procedure was repeated two times. The resulting oil was purified by distillation (8 mmHg, 120 °C) to afford **2d** (7.93 g, 25.0 mmol, 63%) as a colorless oil. ^1H NMR (400 MHz, $CDCl_3$/TMS) δ 1.82 (q, 6H, J = 7.3 Hz), 1.36-1.26 (m, 3H), 1.10 (d, 18H, J = 7.3 Hz), 0.85 (t, 9H, 7.3 Hz). ^{13}C NMR (100 MHz) δ 151.6, 88.6, 26.3, 17.7, 12.0, 7.58. LRMS (FAB) *m/z* 317 [M + H]$^+$. HRMS (FAB-EB) *m/z*: [M + H]$^+$ Calcd. for $C_{17}H_{37}O_3Si$: 317.2507, found: 317.2518. IR (neat): 2947, 2870, 1716, 1460, 1274, 1137, 883 cm^{-1}.

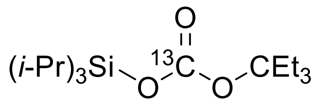

3-Ethylpentan-3-yl (triisopropylsilyl) carbonate-^{13}C (2d*). In a glove box under Ar atmosphere, $LiOCEt_3$[11] (4.88 g, 40.0 mmol) was added to an oven-dried two-neck round flask (200 mL) equipped with a stirrer bar. The flask was sealed with a rubber cap and three-way stopcock, and taken out from the glove box. The flask was evacuated and refilled with $^{13}CO_2$ gas using a $^{13}CO_2$-filled balloon. Subsequently, THF (60 mL) was added. After stirring at 0 °C for 1 h, a solution of triisopropylsilyl chloride (7.72 g, 40.0 mmol) in THF (20 mL) was slowly added. The mixture was further stirred at 0 °C for 1 h. Then, after hexane (60 mL) was added, the mixture was filtered (Celite®, hexane) and concentrated. This procedure was repeated two times. The resulting oil was purified by distillation (8 mmHg, 120 °C) to afford **2d*** (4.71 g, 14.8 mmol, 37%) as a colorless oil. ^1H NMR (400 MHz, $CDCl_3$/TMS) δ 1.82 (q, 6H, J = 7.8 Hz), 1.35-1.26 (m, 3H), 1.10 (d, 18H, J = 7.8 Hz), 0.84 (t, 9H, 7.8 Hz). ^{13}C NMR (100 MHz) δ 151.5, 88.5 (d, J = 1.7 Hz), 26.4 (d, J = 1.7 Hz), 17.7, 12.1, 7.58. LRMS (FAB) *m/z* 318 [M + H]$^+$. HRMS (FAB-EB) *m/z*: [M + H]$^+$ Calcd. for $C_{16}{}^{13}CH_{37}O_3Si$: 318.2540, found: 318.2560. IR (neat): 2946, 2869, 1704, 1673, 1460, 1232, 1136, 883 cm^{-1}.

General procedure of carboxylation of (hetero)aromatic C–H bonds using an alkyl silyl carbonate reagent (Table 1, Scheme 1, and Scheme 2).

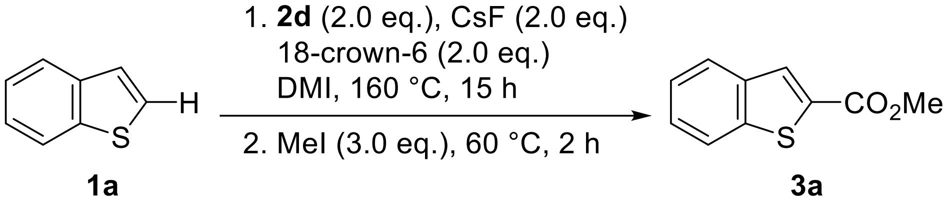

Methyl benzo[*b*]thiophene-2-carboxylate (3a). In a glove box under an Ar atmosphere, a solution of **1a** (27.0 mg, 0.201 mmol), CsF (60.7 mg, 0.400 mmol), 18-crown-6 (105.8 mg, 0.400 mmol), and **2d** (126.6 mg, 0.400 mmol) in DMI (1 mL) was prepared in an oven-dried glass screw tube (ϕ = 1.65 cm, 10.5 cm) equipped with a stirrer bar. The tube was sealed with a cap containing an inner Teflon film. After stirring at 160 °C in a heat block for 15 h, the mixture was added methyl iodide (38 mL, 0.6 mmol) and stirred at 60 °C in a heat block for 2 h. The mixture was extracted with AcOEt (3 mL x 3). The combined organic layer was washed with H_2O (3 mL) and brine (3 mL), dried over Na_2SO_4, and concentrated. The crude material was purified by column chromatography on silica gel (hexane:AcOEt = 20:1) to afford **3a** (37.9 mg, 0.197 mmol, 98%) as a white solid. Mp 69-70 °C (lit. 72-74 °C;[12a] 70-71 °C[12b]). ^{1}H NMR (400 MHz, $CDCl_3$/TMS) δ 8.07 (s, 1H), 7.89-7.86 (m, 2H), 7.48-7.39 (m, 2H), 3.95 (s, 3H). ^{13}C NMR (150 MHz, $CDCl_3$) δ 163.2, 142.2, 138.6, 133.3, 130.6, 126.9, 125.5, 124.9, 122.7, 52.4. LRMS (EI) *m/z*: 192 $[M]^+$. HRMS (EI-TOF) *m/z*: $[M]^+$ Calcd. for $C_{10}H_8O_2S$: 192.0243, found: 192.0245. IR (neat): 2945, 1708, 1520, 1289, 1242, 1173, 760 cm^{-1}. The spectra data matched those reported in the literature.[12b,12c]

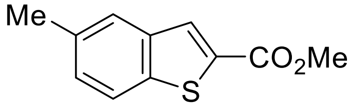

Methyl 5-methylbenzo[*b*]thiophene-2-carboxylate (3b). According to the general procedure analogues to that described for **3a**, **3b** (40.2 mg, 0.195 mmol, 98%) was obtained from **1b** (29.7 mg, 0.200 mmol) as a white solid: Mp 82-83 °C (lit. 78-79 °C (MeOH)[13]). ^{1}H NMR (400 MHz, $CDCl_3$/TMS) δ 7.98 (s,1H), 7.74 (d, 1H, J = 8.8 Hz), 7.66 (s, 1H), 7.29 (d, 1H, J = 8.3 Hz), 3.94 (s, 3H), 2.47 (s, 3H). ^{13}C NMR (100 MHz, $CDCl_3$/TMS) δ 163.3, 139.5, 139.0, 134.7, 133.3, 130.3, 128.9, 125.2, 122.3, 52.4, 21.3. LRMS (EI) *m/z*: 206 $[M]^+$. HRMS (EI-EB) *m/z*: $[M]^+$ Calcd. for $C_{11}H_{10}O_2S$: 206.0402, found: 206.0402. IR (neat): 2950, 1706, 1555, 1442, 1289, 1201, 1146 cm^{-1}. The spectra data matched those reported in the literature.[10a]

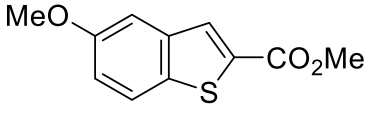

Methyl 5-methoxybenzo[*b*]thiophene-2-carboxylate (3c). According to the general procedure analogues to that described for **3a**, except that the crude material was purified by column chromatography on silica gel (hexane:AcOEt = 5:1), **3c** (42.4 mg, 0.191 mmol, 96%) was obtained from **1c** (32.7 mg, 0.199 mmol) as a white solid: Mp 102-104 °C (lit. 102-103 °C[14]). ^{1}H NMR (400 MHz, $CDCl_3$/TMS) δ 7.98 (s, 1H), 7.72 (d, 1H, J = 8.8 Hz), 7.28 (d, 1H, J = 2.4 Hz), 7.12 (dd, 1H, J = 8.8, 2.4 Hz), 3.94 (s, 3H), 3.88 (s, 3H). ^{13}C NMR (100 MHz, $CDCl_3$/TMS) δ 163.2, 157.8, 139.6, 134.8, 134.2, 130.2, 123.4, 118.0, 106.4, 55.4, 52.4. LRMS (EI) *m/z*: 222 $[M]^+$. HRMS (EI-TOF) *m/z*: $[M]^+$ Calcd. for $C_{11}H_{10}O_3S$: 222.0351, found: 222.0344. IR (neat): 2962, 2931, 1706, 1517, 1456, 1423, 1289, 1077 cm^{-1}. The spectra data matched those reported in the literature.[14]

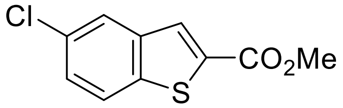

Methyl 5-chlorobenzo[*b*]thiophene-2-carboxylate (3d). According to the general procedure analogues to that described for **2a**, except for that the carboxylation was conducted at 160 °C in a heat block for 3 h, **3d** (43.5 mg, 0.192 mmol, 96%) was obtained from **1d** (33.7 mg, 0.200 mmol) as a white solid: Mp 108-110 °C (lit. 109-110 °C (EtOH)[15]). ^{1}H NMR (400 MHz, $CDCl_3$/TMS) δ 7.95 (s, 1H), 7.82 (d, 1H, J = 1.9 Hz), 7.75 (d, 1H, J = 8.8 Hz), 7.40 (dd, 1H, J = 8.8, 1.9 Hz), 3.95 (s, 3H). ^{13}C NMR (100 MHz, $CDCl_3$/TMS) δ 162.8, 140.1, 139.6, 135.3, 131.2, 129.5, 127.4, 124.8, 123.8, 52.6. LRMS (EI) *m/z*: 226 $[M]^+$. HRMS (EI-TOF) *m/z*: $[M]^+$ Calcd. for $C_{10}H_7ClO_2S$: 225.9855, found: 225.9859. IR (neat): 2933, 1720, 1559, 1517, 1251, 1175 cm^{-1}. The spectra data matched those reported in the literature.[10a]

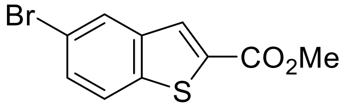

Methyl 5-bromobenzo[*b*]thiophene-2-carboxylate (3e). According to the general procedure analogues to that described for **2a**, except for that the carboxylation was conducted at 160 °C in a heat block for 3 h, **3e** (51.2 mg, 0.190 mmol, 95%) was obtained from **1e** (42.5 mg, 0.199 mmol) as a white solid: Mp 112-114 °C (lit. 110 °C[16]). ^{1}H NMR (400 MHz, $CDCl_3$/TMS) δ 8.03 (d, 1H, J = 1.4 Hz), 7.98 (s, 1H), 7.73 (d, 1H, J = 8.8 Hz), 7.55 (dd, 1H, J = 8.8, 2.0 Hz), 3.95 (s, 3H). ^{13}C NMR (100 MHz, $CDCl_3$/TMS) δ 162.7, 140.6, 140.1, 135.1, 130.0, 129.4, 127.9, 124.1, 118.9, 52.6. LRMS (EI) *m/z*: 272 $[M]^+$. HRMS (EI-TOF) *m/z*: $[M]^+$ Calcd. for $C_{10}H_7BrO_2S$: 269.9350, found: 269.9355. IR (neat): 2967, 1718, 1516, 1283, 1248, 1065 cm^{-1}. The spectra data matched those reported in the literature.[16]

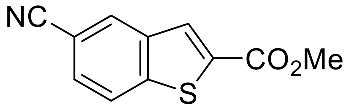

Methyl 5-cyanobenzo[*b*]thiophene-2-carboxylate (3f). According to the general procedure analogues to that described for **2a**, except that the crude material was purified by column chromatography on silica gel (hexane:AcOEt = 5:1), **3f** (42.8 mg, 0.197 mmol, 99%) was obtained from **1f** (31.6 mg, 0.198 mmol) as a white solid: Mp 168-170 °C (lit. 161-162 °C[17]). ^{1}H NMR (400 MHz, $CDCl_3$/TMS) δ 8.22 (s, 1H), 8.10 (s, 1H), 7.98 (d, 1H, J = 8.8 Hz), 7.66 (dd, 1H, J = 8.8, 1.5 Hz), 3.98 (s, 3H). ^{13}C NMR (100 MHz, $CDCl_3$/TMS) δ 162.3, 145.7, 138.4, 136.3, 130.1, 129.8, 128.4, 123.9, 118.7, 109.0, 52.9. LRMS (EI) *m/z*: 217 $[M]^+$. HRMS (EI-TOF) *m/z*: $[M]^+$ Calcd. for $C_{11}H_7NO_2S$: 217.0198, found: 217.0194. IR (neat): 3087, 2965, 2925, 2229, 1720, 1527, 1435, 1248, 1057 cm^{-1}. The spectra data matched those reported in the literature.[17]

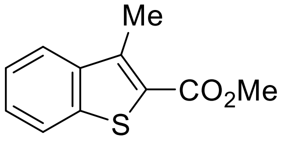

Methyl 3-methylbenzo[*b*]thiophene-2-carboxylate (3g). According to the general procedure analogues to that described for **2a**, **3g** (29.8 mg, 0.144 mmol, 72%) was obtained from **1g** (29.8 mg, 0.201 mmol) as a white solid: 100-103 °C (lit. 102.5-103 °C[18]). ^{1}H NMR (400 MHz, $CDCl_3$/TMS) δ 7.86-7.82 (m, 2H), 7.52-7.41 (m, 2H), 3.93 (s, 3H), 2.79 (s, 3H). ^{13}C NMR (100 MHz, $CDCl_3$/TMS) δ 163.9, 141.2, 140.4, 140.1, 127.1, 126.5, 124.4, 123.6, 122.6, 52.0, 13.1. LRMS (EI) *m/z*: 206 $[M]^+$. HRMS (EI-TOF) *m/z*: $[M]^+$ Calcd. for $C_{11}H_{10}O_2S$: 206.0402, found: 206.0405. IR (neat): 2951, 1707, 1141, 1237,1107 cm^{-1}. The spectra data matched those reported in the literature.[10a]

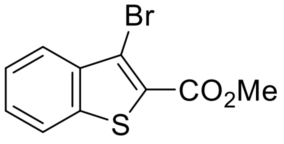

Methyl 3-bromobenzo[*b*]thiophene-2-carboxylate (3h). According to the general procedure analogues to that described for **2a**, except that the carboxylation was conducted at 160 °C in a heat block for 1 h, **3h** (53.5 mg, 0.198 mmol, 99%) was obtained from **1h** (42.6 mg, 0.200 mmol) as a white solid: 68-70 °C (lit. 74-76 °C;[19a] 67-69 °C (AcOEt-petroleum ether)[19b]). ^{1}H NMR (400 MHz, $CDCl_3$/TMS) δ 8.00-7.98 (m, 1H), 7.85-7.82 (m, 1H), 7.56-7.49 (m, 2H), 3.98 (s, 3H). ^{13}C NMR (100 MHz, $CDCl_3$/TMS) δ 161.8, 139.3, 138.5, 128.1, 127.2, 125.6, 125.3, 122.6, 115.0, 52.5. LRMS (EI) *m/z*: 272 $[M]^+$. HRMS (EI-TOF) *m/z*: $[M]^+$ Calcd. for $C_{10}H_7BrO_2S$: 269.9350, found: 269.9356. IR (neat): 2962, 1723, 1517, 1228, 1058 cm^{-1}. The spectra data matched those reported in the literature.[19]

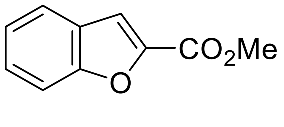

Methyl benzofuran-2-carboxylate (3i). According to the general procedure analogues to that described for **2a**, except for that the carboxylation was conducted at 180 °C in a heat block for 15 h, **3i** (26.0 mg, 0.148 mmol, 74%) was obtained from **1i** (23.8 mg, 0.201 mmol) as a yellow solid: Mp 51-53 °C (lit. 53-55 °C[20]). ^{1}H NMR (400 MHz, $CDCl_3$/TMS) δ 7.69 (d, 1H, J = 7.8 Hz), 7.60 (d, 1H, J = 8.8 Hz), 7.54 (s, 1H), 7.46 (dt, 1H, J = 7.3, 1.5 Hz), 7.31 (t, 1H, J = 7.3 Hz), 3.98 (s, 3H). ^{13}C NMR (150 MHz, $CDCl_3$/TMS) δ 159.9, 155.7, 145.3, 127.6, 126.9, 123.7, 122.8, 114.0, 112.3, 52.3. LRMS (EI) *m/z*: 176 $[M]^+$. HRMS (EI-TOF) *m/z*: $[M]^+$ Calcd. for $C_{10}H_8O_3$: 176.0473, found: 176.0463. IR (neat): 2956, 1734, 1713, 1564, 1296, 1175, 1086 cm^{-1}. The spectra data matched those reported in the literature.[20]

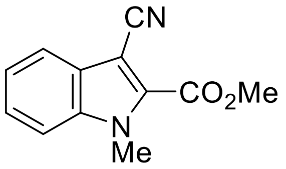

Methyl 3-cyano-1-methyl-1*H*-indole-2-carboxylate (3j). According to the general procedure analogues to that described for **2a**, except that the carboxylation was conducted at 120 °C in a heat block and that the crude material was purified by column chromatography on silica gel (hexane:AcOEt = 2:1), **3j** (42.4 mg, 0.198 mmol, 99%) was obtained from **1j** (31.2 mg, 0.200 mmol) as a white solid: Mp 161-162 °C (lit. 161-162 °C (hexane/AcOEt)[21]). ^{1}H NMR (400 MHz, $CDCl_3$/TMS) δ 7.84 (d, 1H, J = 8.3 Hz), 7.52-7.46 (m, 2H), 7.40-7.34 (m, 1H), 4.15 (s, 3H), 4.05 (s, 3H). ^{13}C NMR (100 MHz, $CDCl_3$/TMS) δ 160.2, 137.9, 131.6, 126.9, 126.6, 123.3, 120.8, 114.7, 111.0, 91.9, 52.5, 32.4. LRMS (EI) *m/z*: 214 $[M]^+$. HRMS (EI-EB) *m/z*: $[M]^+$ Calcd. for $C_{12}H_{10}N_2O_2$: 214.0742, found: 214.0736. IR (neat): 2952, 2223, 1723, 1250, 759, 752 cm^{-1}. The spectra data matched those reported in the literature.[21]

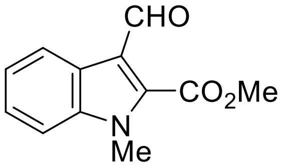

Methyl 3-formyl-1-methyl-1*H*-indole-2-carboxylate (3k). According to the general procedure analogues to that described for **2a**, except that the carboxylation was conducted at 150 °C in a heat block and that the crude material was purified by column chromatography on silica gel (hexane:AcOEt = 5:1), **3k** (35.7 mg, 0.164 mmol, 82%) was obtained from **1k** (31.8 mg, 0.200 mmol) as a white solid: Mp 159-161 °C (lit. 150-152 °C (EtOH)[22]). ^{1}H NMR (400 MHz, $CDCl_3$/TMS) δ 10.59 (s, 1H), 8.52 (d, 1H, J = 8.3 Hz), 7.48-7.43 (m, 2H), 7.39-7.35 (m, 1H), 4.10 (s, 3H), 4.06 (s, 3H). ^{13}C NMR (100 MHz, $CDCl_3$/TMS) δ 188.3, 161.4, 138.2, 133.1, 126.2, 124.4, 124.0, 123.7, 119.8, 110.3, 52.6, 32.4. LRMS (EI) *m/z*: 217 $[M]^+$. HRMS (EI-TOF) *m/z*: $[M]^+$ Calcd. for $C_{12}H_{11}NO_3$: 217.0739, found:

217.0741. IR (neat): 2955, 1714, 1641, 1260, 1245, 1151, 1134, 742 cm^{-1}. The spectra data matched those reported in the literature.[22]

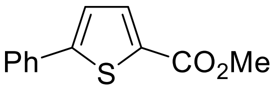

Methyl 5-phenylthiophene-2-carboxylate (3l). According to the general procedure analogues to that described for **2a**, except for that the carboxylation was conducted at 180 °C in a heat block for 15 h, **3l** (41.5 mg, 0.191 mmol, 96%) was obtained from **1l** (32.0 mg, 0.200 mmol) as a white solid: Mp 98-99 °C (lit. 97-98 °C (petroleum ether)[23a]). ^{1}H NMR (400 MHz, $CDCl_3$/TMS) δ 7.77 (d, 1H, J = 3.9 Hz), 7.65-7.63 (m, 2H), 7.43-7.35 (m, 3H), 7.29 (d, 1H, J = 3.9 Hz), 3.91 (s, 3H). ^{13}C NMR (100 MHz, $CDCl_3$/TMS) δ 162.6, 151.2, 134.3, 133.4, 132.0, 129.0, 128.7, 126.2, 123.6, 52.1. LRMS (EI) *m/z*: 218 $[M]^+$. HRMS (EI-TOF) *m/z*: $[M]^+$ Calcd. for $C_{12}H_{10}O_2S$: 218.0402, found: 218.0391. IR (neat): 2929, 1700, 1559, 1540, 1454, 1433, 1347, 1265, 1102 cm^{-1}. The spectra data matched those reported in the literature.[23b, 23c]

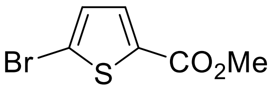

Methyl 5-bromothiophene-2-carboxylate (3m). According to the general procedure analogues to that described for **2a**, **3m** (37.2 mg, 0.169 mmol, 85%) was obtained from **1m** (32.6 mg, 0.200 mmol) as a white solid: Mp 59-60 °C (lit. 58-60 °C;[24a] 56-57 °C[24b]). ^{1}H NMR (400 MHz, $CDCl_3$/TMS) δ 7.55 (d, 1H, J = 3.9 Hz), 7.07 (d, 1H, J = 4.4 Hz), 3.87 (s, 3H). ^{13}C NMR (150 MHz, $CDCl_3$/TMS) δ 161.5, 134.7, 133.7, 130.9, 120.2, 52.3. LRMS (EI) *m/z*: 220 $[M]^+$. HRMS (EI-TOF) *m/z*: $[M]^+$ Calcd. for $C_6H_5BrO_2S$: 219.9194, found: 219.9193. IR (neat): 2965, 1710, 1437, 1332, 1260, 1093 cm^{-1}. The spectra data matched those reported in the literature.[24b, 24c]

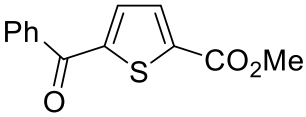

Methyl 5-benzoylthiophene-2-carboxylate (3n). According to the general procedure analogues to that described for **2a**, **3n** (49.2 mg, 0.200 mmol, 99%) was obtained from **1n** (38.0 mg, 0.202 mmol) as a white solid: Mp 69-70 °C (lit. 72-73 °C (hexane-AcOEt)[10a]). ^{1}H NMR (400 MHz, $CDCl_3$/TMS) δ 7.89-7.87 (m, 2H), 7.80 (d, 1H, J = 3.9 Hz), 7.65-7.60 (m, 2H), 7.54-7.50 (m, 2H), 3.94 (s, 3H). ^{13}C NMR (100 MHz, $CDCl_3$/TMS) δ 188.0, 162.1, 147.8, 139.7, 137.2, 133.8, 133.1, 132.8, 129.3, 128.6, 52.6. LRMS (EI) *m/z*: 246 $[M]^+$. HRMS (EI-TOF) *m/z*: $[M]^+$ Calcd. for $C_{13}H_{10}O_3S$: 246.0351, found: 246.0357. IR (neat): 2950, 1715, 1635, 1525,1283, 1102 cm^{-1}. The spectra data matched those reported in the literature.[10a]

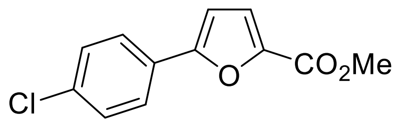

Methyl 5-(4-chlorophenyl)furan-2-carboxylate (3o). According to the general procedure analogues to that described for **2a**, except that the carboxylation was carried out with CsF (91.0 mg, 0.599 mmol), 18-crown-6 (158.5 mg, 0.599 mmol), and **2d** (189.9 mg, 0.600 mmol) at 180 °C in a heat block for 15 h, **3o** (22.4 mg, 0.0946 mmol, 47%) was obtained from **1o** (36.3 mg, 0.203 mmol) as a white solid: Mp 130-131 °C (lit. 130-132 °C[25]). ^{1}H NMR (400 MHz, $CDCl_3$/TMS) δ 7.72 (d, 2H, J = 8.8 Hz), 7.40 (d, 2H, J = 8.8 Hz), 7.24 (d, 1H, J = 3.4 Hz), 6.73 (d, 1H, J = 3.4 Hz), 3.92 (s, 3H). ^{13}C NMR (100 MHz, $CDCl_3$/TMS) δ 159.1, 156.4, 143.8, 134.8, 129.1, 127.9, 126.0, 120.0, 107.2, 51.9. LRMS (EI) *m/z*: 236 $[M]^+$. HRMS (EI-EB) *m/z*: $[M]^+$ Calcd. for $C_{12}H_9ClO_3$: 236.0240, found: 236.0251. IR (neat): 2947, 1725, 1472, 1435, 1298, 1137 cm^{-1}. The spectra data matched those reported in the literature.[25]

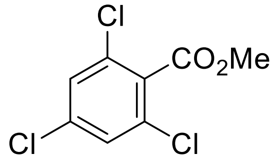

Methyl 2,4,6-trichlorobenzoate (5a). According to the general procedure analogues to that described for **2a**, except for that the carboxylation was conducted at 100 °C in a heat block for 5 h and that the crude material was purified by chromatography on silica gel (hexane:AcOEt = 40:1), **5a** (45.1 mg, 0.188 mmol, 94%) was obtained from **4a** (36.5 mg, 0.201 mmol) as a white solid: Mp 41-43 °C (lit. 42-43 °C (hexane)[26]). ^{1}H NMR (400 MHz, $CDCl_3$/TMS) δ 7.35 (s, 2H), 3.97 (s, 3H). ^{13}C NMR (100 MHz, $CDCl_3$/TMS) δ 164.5, 136.2, 132.7, 132.1, 128.0, 53.1. LRMS (EI) *m/z*: 238 $[M]^+$. HRMS (EI-EB) *m/z*: $[M]^+$ Calcd. for $C_8H_5Cl_3O_2$: 237.9355, found: 237.9351. IR (neat): 2962, 1744, 1580, 1383, 1266, 1115, 846 cm^{-1}. The spectra data matched those reported in the literature.[26]

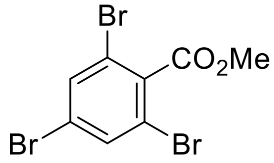

Methyl 2,4,6-tribromobenzoate (5b). According to the general procedure analogues to that described for **2a**, except for that the carboxylation was conducted with CsF (91.6 mg, 0.603 mmol), 18-crown-6 (158.2 mg, 0.599 mmol), and **2d** (191.2 mg, 0.605 mmol) at 100 °C in a heat block for 5 h and that the crude material was purified by chromatography on silica gel (hexane:AcOEt = 40:1), **5b** (61.0 mg, 0.164 mmol, 82%) was obtained from **4b** (62.7 mg, 0.199 mmol) as a white solid: Mp 65-66 °C (lit. 65-66 °C (hexane)[26]). ^{1}H NMR (400 MHz, $CDCl_3$/TMS) δ 7.71 (s, 2H), 3.97 (s, 3H). ^{13}C NMR (100 MHz, $CDCl_3$/TMS) δ 165.9, 136.6, 134.0, 124.0, 120.2, 53.1. LRMS (EI) *m/z*: 372 $[M]^+$. HRMS (EI-

EB) *m/z*: $[M]^+$ Calcd. for $C_8H_5Br_3O_2$: 369.7840, found: 369.7847. IR (neat): 2960, 1739, 1540, 1271, 1193, 1136, 832 cm^{-1}. The spectra data matched those reported in the literature.[26]

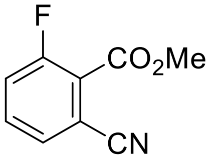

Methyl 2-cyano-6-fluorobenzoate (5c). According to the general procedure analogues to that described for **2a**, except for that the carboxylation was conducted at 130 °C in a heat block and that the crude material was purified by preparative thin-layer chromatography (hexane:AcOEt = 10:1), **5c** (23.8 mg, 0.133 mmol, 67%) was obtained from **4c** (24.2 mg, 0.200 mmol) as a white solid: Mp 70-75 °C (lit. 69-70 °C (hexane/AcOEt)[26]). ^{1}H NMR (400 MHz, $CDCl_3$/TMS) δ 7.64-7.56 (m, 2H), 7.46-7.39 (m, 1H), 4.03 (s, 3H). ^{13}C NMR (100 MHz, $CDCl_3$/TMS) δ 162.5 (d, $^3J_{CF}$ = 2.0 Hz), 160.7 (d, $^1J_{CF}$ = 258.9 Hz), 133.4 (d, $^3J_{CF}$ = 10.0 Hz), 130.0 (d, $^3J_{CF}$ = 4.0 Hz), 122.9 (d, $^3J_{CF}$ = 16.0 Hz), 121.5 (d, $^2J_{CF}$ = 22.0 Hz), 116.1 (d, $^4J_{CF}$ = 4.0 Hz), 114.1 (d, $^4J_{CF}$ = 4.0 Hz), 53.2. ^{19}F NMR (376.1 MHz, $CDCl_3$/4-fluorotoluene) δ -106.8. LRMS (EI) *m/z*: 179 $[M]^+$. HRMS (EI-TOF) *m/z*: $[M]^+$ Calcd. for $C_9H_6FNO_2$: 179.0383, found: 179.0388. IR (neat): 3090, 2959, 2243, 1726, 1578 cm^{-1}. The spectra data matched those reported in the literature.[26]

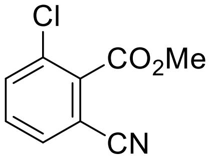

Methyl 2-chloro-6-cyanobenzoate (5d). According to the general procedure analogues to that described for **2a**, except that the carboxylation was conducted at 130 °C in a heat block and that the crude material was purified by column chromatography on silica gel (hexane:AcOEt = 10:1), **5d** (26.9 mg, 0.138 mmol, 68%) was obtained from **4d** (27.6 mg, 0.201 mmol) as a white solid: Mp 72-75 °C (lit. 72-74 °C (hexane/AcOEt)[26]). ^{1}H NMR (400 MHz, $CDCl_3$/TMS) δ 7.73-7.61 (m, 2H), 7.50 (t, 1H, J = 8.1 Hz), 4.04 (s, 3H). ^{13}C NMR (100 MHz, $CDCl_3$/TMS) δ 164.2, 135.7, 134.5, 133.1, 131.42, 131.36, 115.8, 112.9, 53.4. LRMS (EI) *m/z*: 195 $[M]^+$. HRMS (EI-TOF) *m/z*: $[M]^+$ Calcd. for $C_9H_6ClNO_2$: 195.0087, found: 195.0078. IR (neat): 3081, 2238, 1724, 1561, 1460, 1282, 1110 cm^{-1}. The spectra data matched those reported in the literature.[26]

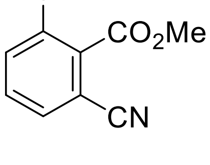

Methyl 2,6-dicyanobenzoate (5e). According to the general procedure analogues to that described for **2a**, except that the carboxylation was conducted at 130 °C in a heat block and that the crude material was purified by column chromatography on silica gel (CH_2Cl_2), **5e** (35.2 mg, 0.189 mmol, 94%) was obtained from **4e** (25.7 mg, 0.201 mmol) as a white solid: Mp 189-190 °C. ^{1}H NMR (400 MHz, $CDCl_3$/TMS) δ 8.02 (d, 2H, J = 7.3 Hz), 7.78 (t, 1H, J = 7.8 Hz), 4.11 (s, 3H). ^{13}C NMR (100 MHz, $CDCl_3$/TMS) δ 162.5, 137.8, 136.4, 132.4, 115.6, 114.5, 53.8. LRMS (EI) *m/z*: 186 $[M]^+$. HRMS (EI-EB) *m/z*: $[M]^+$ Calcd. for $C_{10}H_6N_2O_2$: 186.0429, found: 186.0434. IR (neat): 3073, 2243, 2237, 1728, 1582, 1456, 1440 cm^{-1}. The spectra data matched those reported in the literature.[26]

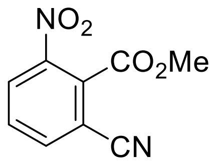

Methyl 2-cyano-6-nitrobenzoate (5f). According to the general procedure analogues to that described for **2a**, except that the carboxylation was conducted at 90 °C in a heat block for 15 h and that the crude material was purified by column chromatography on silica gel (hexane:CH_2Cl_2 = 1:10), **5f** (25.8 mg, 0.125 mmol, 63%) was obtained from **4f** (29.4 mg, 0.198 mmol) as a yellow solid: 102-105 °C (lit. 106–108 °C (hexane/AcOEt)[26]). ^{1}H NMR (400 MHz, $CDCl_3$/TMS) δ 8.34 (dd, 1H, J = 8.5, 1.2 Hz), 8.01 (dd, 1H, J = 7.8, 1.0 Hz), 7.77 (t, 1H, J = 8.0 Hz), 4.07 (s, 3H). ^{13}C NMR (100 MHz, $CDCl_3$/TMS) δ 163.5, 146.5, 137.6, 132.7, 131.5, 128.2, 114.6, 113.1, 54.2. LRMS (EI) *m/z*: 206 $[M]^+$. HRMS (EI-TOF) *m/z*: $[M]^+$ Calcd. for $C_9H_6N_2O_4$: 206.0328, found: 206.0335. IR (neat): 3104, 2239, 1733, 1535, 1363, 1278 cm^{-1}. The spectra data matched those reported in the literature.[26]

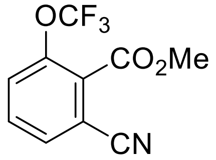

Methyl 2-cyano-6-(trifluoromethoxy)benzoate (5g). According to the general procedure analogues to that described for **2a**, except that the carboxylation was conducted at 120 °C in a heat block for 15 h and that the crude material was purified by column chromatography on silica gel (hexane:AcOEt = 10:1) and GPC ($CHCl_3$), **5g** (19.1 mg, 0.078 mmol, 40%) was obtained from **4g** (37.2 mg, 0.199 mmol) as a colorless oil. ^{1}H NMR (600 MHz, $CDCl_3$/TMS) δ 7.71 (d, 1H, J = 6.8 Hz), 7.64 (t, 1H, J = 7.9 Hz), 7.60-7.58 (m, 1H), 4.03 (s, 3H). ^{13}C NMR (100 MHz, $CDCl_3$/TMS) δ 162.8, 146.9, 132.5, 131.9, 129.8, 126.0, 120.1 (q, $^1J_{CF}$ = 258.5), 115.6, 113.9, 53.4. ^{19}F NMR (376.1 MHz, $CDCl_3$/4-fluorotoluene) δ -56.8. LRMS (EI) *m/z*: 245 $[M]^+$. HRMS (EI-EB) *m/z*: $[M]^+$ Calcd. for $C_{10}H_6F_3NO_3$: 245.0300, found: 245.0308. IR (neat): 2965, 2235, 1739, 1600, 1578, 1464, 1435 cm^{-1}. The spectra data matched those reported in the literature.[26]

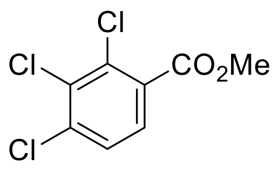

Methyl 2,3,4-trichlorobenzoate (5h). According to the general procedure analogues to that described for **2a**, **5h** (36.3 mg, 0.153 mmol, 76%) was obtained from **4h** (36.5 mg, 0.201 mmol) as a white solid: Mp 63-66 °C (lit. 63-64 °C (hexane)[26]). ^{1}H NMR (400 MHz, $CDCl_3$/TMS) δ 7.63 (d, 1H, J = 8.8 Hz), 7.44 (d, 1H, J = 8.8 Hz), 3.94 (s, 3H). ^{13}C NMR (100 MHz, $CDCl_3$/TMS) δ 165.1, 137.5, 133.7, 133.5, 130.7, 128.9, 128.1, 52.8. LRMS (EI) *m/z*: 238 $[M]^+$. HRMS (EI-EB) *m/z*: $[M]^+$ Calcd. for $C_8H_5Cl_3O_2$: 237.9355, found: 237.9359. IR (neat): 3095, 2959, 1732, 1576, 1425, 1361 cm^{-1}. The spectra data matched those reported in the literature.[26]

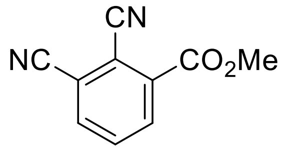

Methyl 2,3-dicyanobenzoate (5i). According to the general procedure analogues to that described for **2a**, except that the carboxylation was conducted at 150 °C in a heat block and that the crude material was purified by column chromatography on silica gel (hexane:AcOEt = 2:1), **5i** (29.6 mg, 0.159 mmol, 79%) was obtained from **4i** (25.8 mg, 0.201 mmol) as a pale yellow solid: Mp 180-182 °C (lit. 178-180 °C (hexane/CH_2Cl_2)[26]). ^{1}H NMR (400 MHz, $CDCl_3$/TMS) δ 8.39 (dd, 1H, J = 8.1, 1.2 Hz), 8.01 (dd, 1H, J = 8.0, 1.2 Hz), 7.84 (t, 1H, J = 7.8 Hz), 4.06 (s, 3H). ^{13}C NMR (100 MHz, $CDCl_3$/TMS) δ 162.9, 136.6, 134.8, 134.1, 132.9, 118.7, 116.2, 114.9, 114.0, 53.5. LRMS (EI) *m/z*: 186 $[M]^+$. HRMS (EI-TOF) *m/z*: $[M]^+$ Calcd. for $C_{10}H_6N_2O_2$: 186.0429, found: 186.0435. IR (neat): 2236, 1723, 1579, 1437, 1290, 776 cm^{-1}. The spectra data matched those reported in the literature.[26]

Procedure of carboxylation of 1a on a 1.0 mmol scale (Table 1, Entry 8).

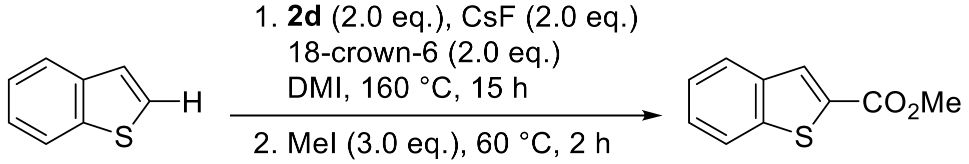

1a
1.00 mmol

3a
0.900 mmol, 90%

In a glove box under an Ar atmosphere, a solution of **1a** (134.7 mg, 1.00 mmol), CsF (303.8 mg, 2.00 mmol), 18-crown-6 (545.8 mg, 2.07 mmol), and **2d** (636.1 mg, 2.01 mmol) in DMI (5 mL) was prepared in an oven-dried glass screw tube (ϕ = 2.5 cm, 15.0 cm) equipped with a stirrer bar. The tube was sealed with a cap containing an inner Teflon film. After stirring at 160 °C in a heat block for 15 h, the mixture was added methyl iodide (0.19 mL, 3.1 mmol) and stirred at 60 °C in a heat block for 2 h.

The mixture was extracted with AcOEt (10 mL x 3). The combined organic layer was washed with H_2O (10 mL) and brine (10 mL), dried over Na_2SO_4, and concentrated. The crude material was purified by column chromatography on silica gel (hexane:AcOEt = 20:1) to afford **3a** (173.0 mg, 0.900 mmol, 90%) as a white solid.

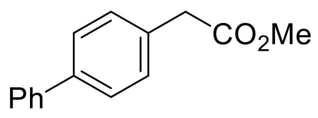

Methyl 2-([1,1'-biphenyl]-4-yl)acetate (7). In a glove box under an Ar atmosphere, a solution of **6** (33.5 mg, 0.199 mmol), CsF (151.7 mg, 1.0 mmol), and **2d** (316.3 mg, 1.0 mmol) in DMI (1 mL) was prepared in an oven-dried glass screw tube (ϕ = 1.65 cm, 10.5 cm) equipped with a stirrer bar. The tube was sealed with a cap containing an inner Teflon film. After stirring at 180 °C in a heat block for 15 h, 1 M aqueous HCl (4 mL) was added to the reaction mixture at 0 ºC. After stirring at 80 °C in a heat block for 4 h, the mixture was extracted with AcOEt (3 mL x 3). The combined organic layer was washed with brine (10 mL), dried over Na_2SO_4, and concentrated. To a solution of the residue in MeOH (1.0 mL) was added trimethylsilyldiazomethane in hexane (0.6 M, 1.25 mL, 0.75 mmol) at 0 °C. After stirring at room temperature for 20 min, the mixture was concentrated. The crude material was purified by preparative thin-layer chromatography (hexane:AcOEt = 20.1) to afford **7** (24.3 mg, 0.107 mmol, 54%) as a colorless oil. ^{1}H NMR (400 MHz, $CDCl_3$/TMS) δ 7.59-7.55 (m, 4H), 7.43 (t, 2H, J = 7.6 Hz), 7.37-7.32 (m, 3H), 3.72 (s, 3H), 3.67 (s, 2H). ^{13}C NMR (150 MHz, $CDCl_3$/TMS) δ 172.0, 140.8, 140.1, 133.0, 129.6, 128.7, 127.3, 127.2, 127.0, 52.1, 40.8. LRMS (EI) *m/z*: 226 $[M]^+$. HRMS (EI-TOF) *m/z*: $[M]^+$ Calcd. for $C_{15}H_{14}O_2$: 226.0994, found: 226.0986. IR (neat): 3031, 1734, 1009, 822, 755 cm^{-1}. The spectra data matched those reported in the literature.[27]

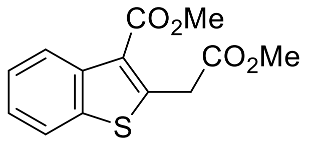

Methyl 2-(2-methoxy-2-oxoethyl)benzo[*b*]thiophene-3-carboxylate (9). In a glove box under an Ar atmosphere, a solution of **8** (29.8 mg, 0.200 mmol), CsF (152.8 mg, 1.01 mmol), and **2d** (325.0 mg, 1.03 mmol) in DMI (1 mL) was prepared in an oven-dried glass screw tube (ϕ = 1.65 cm, 10.5 cm) equipped with a stirrer bar. The tube was sealed with a cap containing an inner Teflon film. After stirring at 160 °C in a heat block for 13 h, 1 M aqueous HCl (3 mL) was added to the reaction mixture at 0 ºC. The mixture was extracted with AcOEt (3 mL x 3). The combined organic layer was washed with brine (10 mL), dried over Na_2SO_4, and concentrated. To a solution of the residue in MeOH (1.0 mL) was added trimethylsilyldiazomethane in hexane (0.6 M, 1.5 mL, 0.9 mmol) at 0 °C. After stirring at room temperature for 20 min, the mixture was concentrated. The crude material was purified by preparative thin-layer chromatography (hexane:AcOEt = 20:1) to afford **9** (35.3 mg, 0.134 mmol, 67%)

as a white solid: Mp 85-86 °C (lit. 89-91 °C (hexane)[28]). ^{1}H NMR (400 MHz, $CDCl_3$/TMS) δ 8.44 (d, 1H, J = 7.8 Hz), 7.79 (d, 1H, J = 7.8 Hz), 7.45 (td, 1H, J = 8.3, 1.0 Hz), 7.37 (td, 1H, J = 8.3, 1.0 Hz), 4.28 (s, 2H), 3.96 (s, 3H), 3.75 (s, 3H). ^{13}C NMR (150 MHz, $CDCl_3$/TMS) δ 169.9, 163.8, 146.9, 137.8, 137.7, 125.3, 124.90, 124.87, 124.1, 121.7, 52.3, 51.6, 35.8. LRMS (EI) *m/z*: 264 $[M]^+$. HRMS (EI-TOF) *m/z*: $[M]^+$ Calcd. for $C_{13}H_{12}O_4S$: 264.0456, found: 264.0445. IR (neat): 2960, 1746, 1696, 1194, 1178, 1016, 749 cm^{-1}. The spectra data matched those reported in the literature.[28]

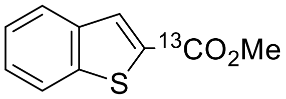

Methyl benzo[*b*]thiophene-2-carboxylate-^{13}*C* (3a*). According to the general procedure analogues to that described for **3a**, except for that the carboxylation was conducted with **2d*** (128.1 mg, 0.403 mmol), **3a*** (36.3 mg, 0.188 mmol, 95%) was obtained from **1a** (26.6 mg, 0.198 mmol) as a white solid. Mp 68-70 °C. ^{1}H NMR (400 MHz, $CDCl_3$/TMS) δ 8.07 (d, 1H, J = 2.9 Hz), 7.88 (t, 2H, J = 6.6 Hz), 7.48-7.39 (m, 2H), 3.95 (d, 3H, J = 3.9 Hz). ^{13}C NMR (100 MHz, $CDCl_3$) δ 163.2, 142.2, 138.7 (d, J = 6.6 Hz), 133.3 (d, J = 84.8 Hz), 130.6 (d, J = 3.3 Hz), 126.9, 125.5, 124.9, 122.7, 52.4 (d, J = 2.5 Hz). LRMS (EI) *m/z*: 193 $[M]^+$. HRMS (EI-TOF) *m/z*: $[M]^+$ Calcd. for $C_9{}^{13}CH_8O_2S$: 193.0279, found: 193.0288. IR (neat): 2950, 1708, 1519, 1265, 1225, 1170, 758 cm^{-1}.

The ^{13}C incorporation (97%) was determined by quantitative ^{13}C NMR analysis. NMR experiment details for ^{13}C incorporation: JEOL ECA600 spectrometer; coupled to ^{1}H; relaxation delay = 28 s, 4096 scans.

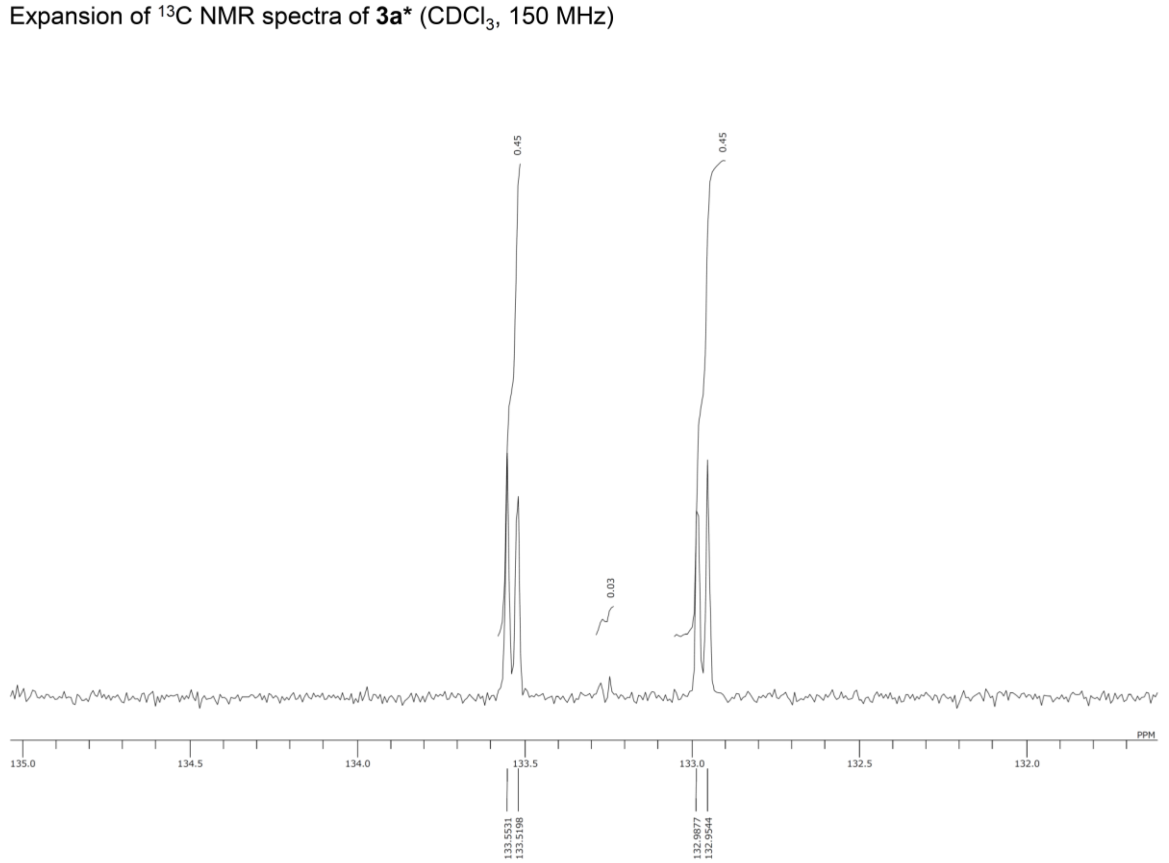

Figure S1. ^{13}C incorporation analysis of **3a***.

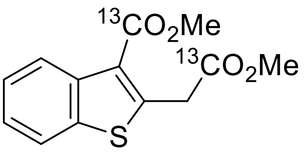

Methyl 2-(2-methoxy-2-oxoethyl-2-^{13}*C*)benzo[*b*]thiophene-3-carboxylate-^{13}*C* (9*). According to the procedure analogues to that described for **9**, except for that the carboxylation was conducted with **2d*** (314.7 mg, 0.992 mmol), **9*** (35.9 mg, 0.135 mmol, 67%) was obtained from **8** (29.8 mg, 0.201 mmol) as a white solid: Mp 83-84 °C. ^{1}H NMR (400 MHz, $CDCl_3$/TMS) δ 8.44 (d, 1H, J = 8.3 Hz), 7.79 (d, 1H, J = 8.8 Hz), 7.45 (td, 1H, J = 7.1, 1.2 Hz), 7.38 (td, 1H, J = 7.3, 1.0 Hz), 4.28 (d, 2H, J = 8.8 Hz), 3.96 (d, 3H, J = 4.4 Hz), 3.75 (d, 3H, J = 3.9 Hz). ^{13}C NMR (100 MHz, $CDCl_3$/TMS) δ 169.9, 163.8, 146.8, 137.8, 137.7, 125.3, 124.89, 124.87, 124.1 (d, J = 79.8 Hz), 121.7, 52.3, 51.5, 35.8 (d, J = 57.6 Hz). LRMS (EI) *m/z*: 266 $[M]^+$. HRMS (EI-TOF) *m/z*: $[M]^+$ Calcd. for $C_{11}{}^{13}C_2H_{12}O_4S$: 266.0523, found: 266.0535. IR (neat): 2960, 1703, 1657, 1192, 1155, 1013, 762 cm^{-1}.

The ^{13}C incorporation at 3-position (96%) and methyl group (98%) was determined by quantitative ^{13}C NMR analysis. NMR experiment details for ^{13}C incorporation: JEOL ECA600 spectrometer; coupled to ^{1}H; relaxation delay = 30 s, 4096 scans.

Expansion of ^{13}C NMR spectra of **9*** ($CDCl_3$, 150 MHz)

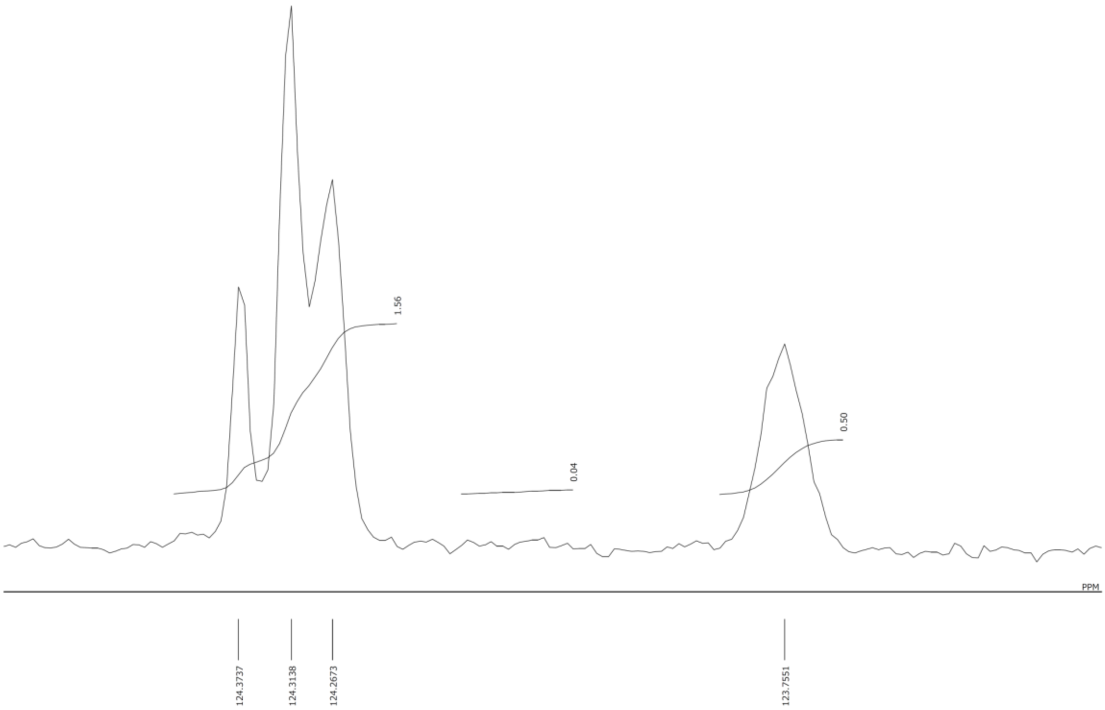

Expansion of ^{13}C NMR spectra of **9*** ($CDCl_3$, 150 MHz)

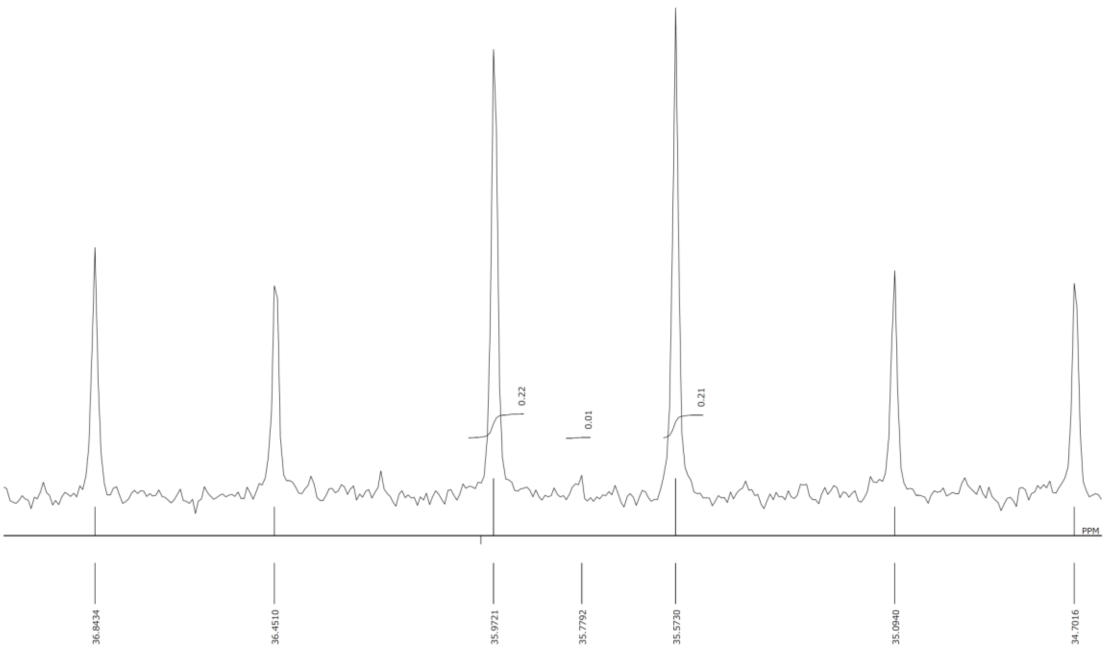

Figure S2. ^{13}C incorporation analysis of **3a***.

References

1. S. Pérez-Silanes, J. Martínez-Esparza, A. M. Oficialdegui, H. Villanueva, L. Orús, A. Monge, *J. Heterocyclic Chem.* **2001**, *38*, 1025.

2. A. J. Seed, K. J. Toyne, J. W. Goodby, M. Hird, *J. Mater. Chem.* **2000**, *10*, 2069.

3. S. K. Thompson, T. Priestley, M. Kundu, A. Saha, S. Nath, PCT Int. Appl. WO2018064135A1.

4. S. Ushijima, K. Moriyama, H. Togo, *Tetrahedron* **2012**, *68*, 4588.

5. S. A. Everett, M. A. Naylor, M. R. L. Stratford, K. B. Patel, E. Ford, A. Mortensen, A. C. Ferguson, B. Vojnovic, P. Wardman, *J. Chem. Soc., Perkin Trans. 2.* **2001**, 1989.

6. M. Herder, F. Eisenreich, A. Bonasera, A. Grafl, L. Grubert, M. Pätzel, J. Schwarz, S. Hecht, *Chem. Eur. J.* **2017**, *23*, 3743.

7. C. K. Hazra, N. Gandhamsetty, S. Park, S. Chang, *Nature Commun.* **2016**, *7*, 13431.

8. Y. Yamamoto, D. S. Tarbell, *J. Org. Chem.* **1971**, *36*, 2954.

9. S. Kawai, T. Nakashima, Y. Kutsunugi, H. Nakagawa, H. Nakano, T. Kawai, *J. Mater. Chem.* **2009**, 19, 3606.

10. (a) M. Shigeno, K. Hanasaka, K. Sasaki, K. Nozawa-Kumada, Y. Kondo, *Chem. Eur. J.* **2019**, *25*, 3235. (b) H. Yildirimyan, G. Gattow, *Z. Anorg. Allg. Chem.* **1984**, *519*, 204. (c) E. E. Coyle, B. J. Doonan, A. J. Holohan, K. A. Walsh, F. Lavigne, E. H. Krenske, C. J. O'Brien, *Angew. Chem. Int. Ed.* **2014**, *53*, 12907.

11. H-Q. Do, R. M. K. Khan, O. Daugulis, *J. Am. Chem. Soc.* **2008**, *130,* 15185.

12. (a) P. S. Mahajan, M. D. Nikaml. U. Nawale, V. M. Khedkar, D. Sarkar, C. H. Gill, *ACS Med. Chem. Lett.* **2016**, *7*, 751. (b) S. Ushijima, K. Moriyama, H. Togo, *Tetrahedron* **2012**, *68*, 4701. (c) D. P. Hari, T. Hering, B. König, *Org. Lett.* **2012**, *14*, 5334.

13. P. E. Cross, R. P. Dickinson, M. J. Parry, M. J. Randall, *J. Med. Chem.* **1986**, *29*, 1637.

14. F. Zeng, H. Alper, *Org. Lett.* **2011**, *13*, 2868.

15. J. R. Beck, *J. Org. Chem.* **1972**, *37*, 3224.

16. N. Terzić, J. Konstantinović, M. Tot, J. Burojević, O. Djurković-Djaković, J. Srbljanović, T. Štajner, T. Verbić, M. Zlatović, M. Machado, I. S. Albuquerque, M. Prudêncio, R. J. Sciotti, S. Pecic, S. D'Alessandro, D. Taramelli, B. A. Šolaja, *J. Med. Chem.* **2017**, *59*, 264.

17. X. Jiang, J. Wang, Y. Zhang, Z. Chen, Y. Zhu, S. Ji, *Tetrahedron* **2015**, *71*, 4883.

18. T. Higa, A. J. Krubsack, *J. Org. Chem.* **1976**, *41*, 3399.

19. (a) R. Romagnoli, P. G. Baraldi, M. K. Salvador, D. Preti, M. A. Tabrizi, M. Bassetto, A. Brancale, E. Hamel, I. Castagliuolo, R. Bortolozzi, G. Basso, G. Viola, *J. Med. Chem.* **2013**, *56*, 2606. (b) M. R.P. Queiroz, R. C. Calhelha, L. A. Vale-Silva, E. Pinto, M. S. Nascimento, *Eur. J. Med. Chem.* **2009**, *44*, 1893.

20. Y. Yamamoto, *Adv. Synth. Catal.* **2010**, *352*, 478.

21. M. Shigeno, I. Tohara, K. Nozawa-Kumada, Y. Kondo, *Eur. J. Org. Chem.* **2020**, 1987.

22. A. S. Bourlot, E. Desarbre, J. Y. Mérour, *Synthesis* **1994**, 411.

23. (a) D. Mackay, *Can. J. Chem.* **1966**, *44*, 2881. (b) T. Noguchi, M. Hasegawa, K. Tomisawa, M. Kitsukuchi, *Bioorg. Med. Chem.* **2003**, *11*, 4729. (c) L. A. McAllister, M. S. Hixon, J. P. Kennedy, T. J. Dickerson, K. D. Janda, *J. Am. Chem. Soc.* **2006**, *128*, 4176.
24. (a) O. Castillo-Aguilera, P. Depreux, L. Halby, N. Azaroual, P. B. Arimondo, L. Goossens, *Tetrahedron Lett.* **2017**, *58*, 2537. (b) Q.-Q. Yang, M. Marchini, W.-J. Xiao, P. Ceroni, M. Bandini, *Chem. Eur. J.* **2015**, *21*, 18052. (c) Z. Tang, Q. Jiang, L. Peng, X. Xu, J. Li, R. Qiu, C.-T. Au, *Green. Chem.* **2017**, *19*, 5396.
25. Y. Zhu, P. Xu, Y. Gong, *J. Org. Chem.* **2016**, *81*, 4829.
26. M. Shigeno, K. Hanasaka, I. Tohara, K. Izumi, H. Yamakoshi, E. Kwon, K. Nozawa-Kumada, Y. Kondo, *Org. Lett.* **2022**, *24*, 809.
27. C. Li, P. Zhao, R. Li, B. Zhang, W. Zhao. *Angew. Chem. Int. Ed.* **2020**, *59,* 10913.
28. M. Shigeno, K. Sasaki, K. Nozawa-Kumada, Y. Kondo, *Org. Lett.* **2019**, *21*, 4515.

^{1}H NMR spectra of **2b** ($CDCl_3$, 400 MHz)

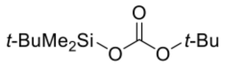

2b

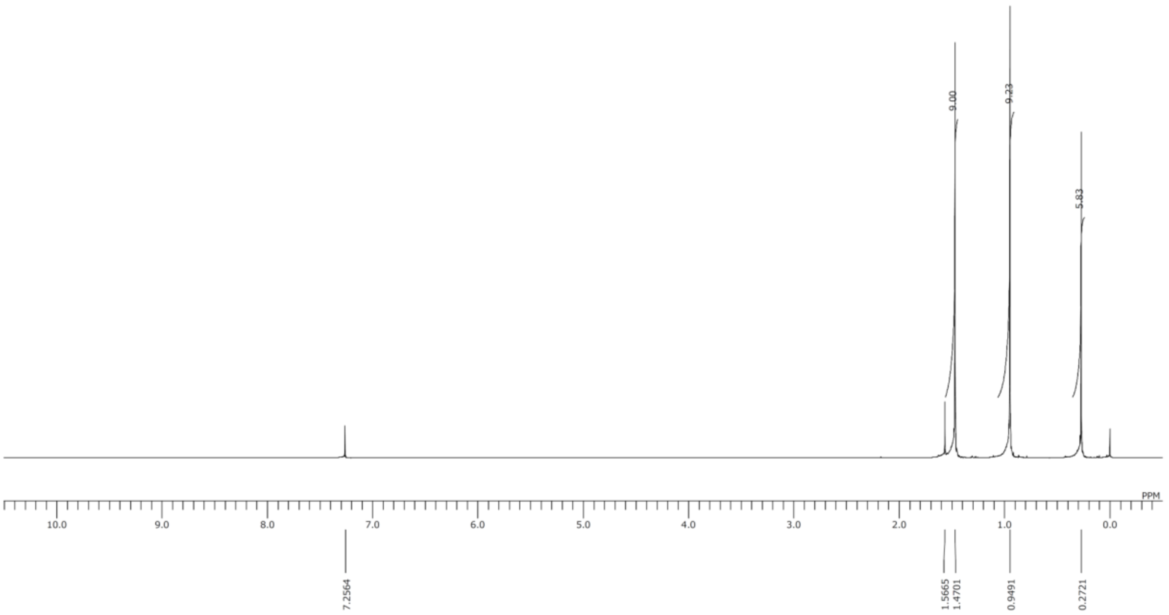

^{13}C NMR spectra of **2b** ($CDCl_3$, 100 MHz)

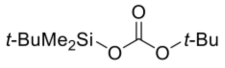

2b

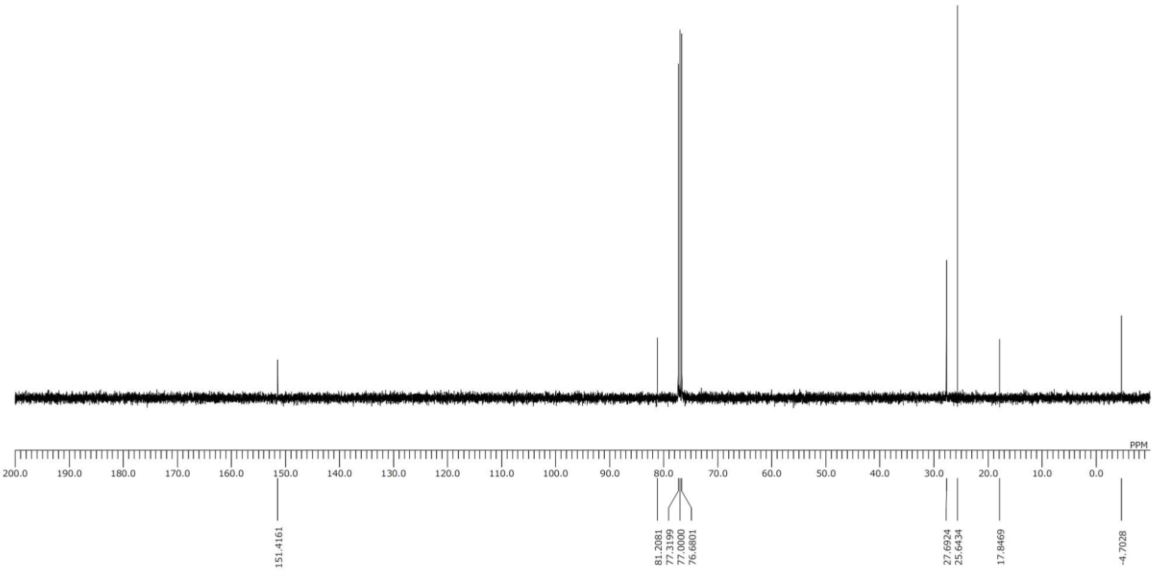

^{1}H NMR spectra of **2c** ($CDCl_3$, 400 MHz)

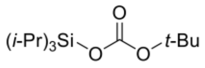

2c

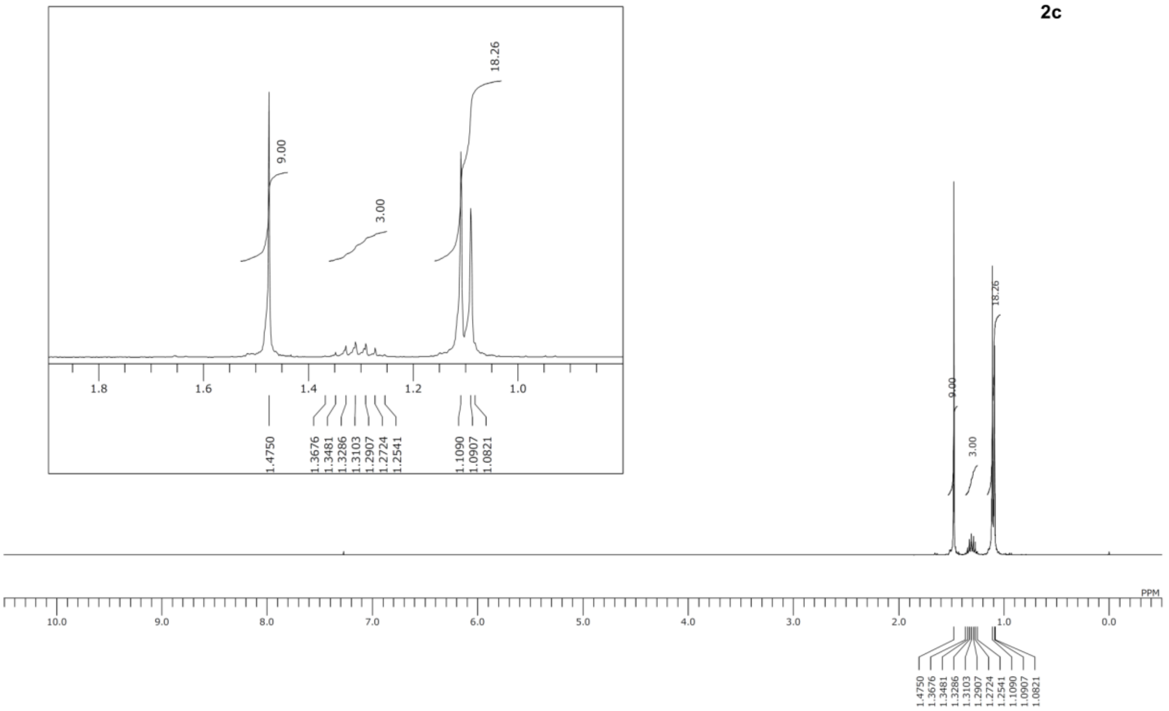

^{13}C NMR spectra of **2c** ($CDCl_3$, 100 MHz)

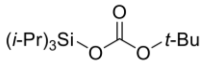

2c

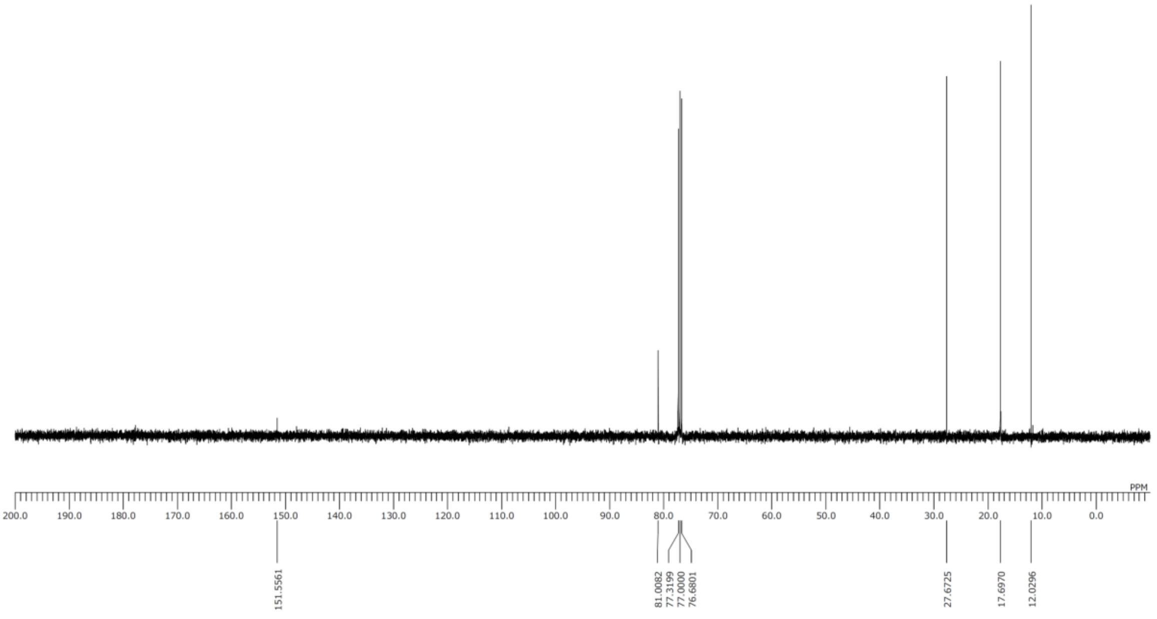

^{1}H NMR spectra of **2d** ($CDCl_3$, 400 MHz)

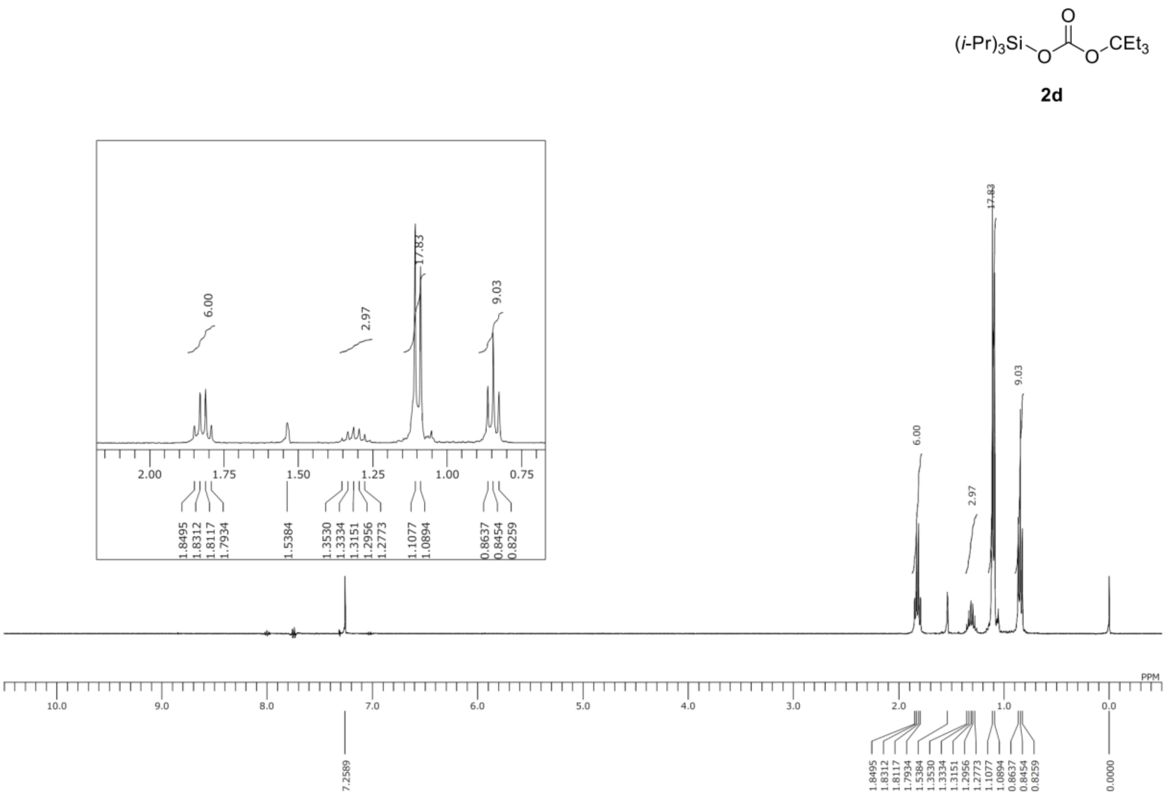

^{13}C NMR spectra of **2d** ($CDCl_3$, 100 MHz)

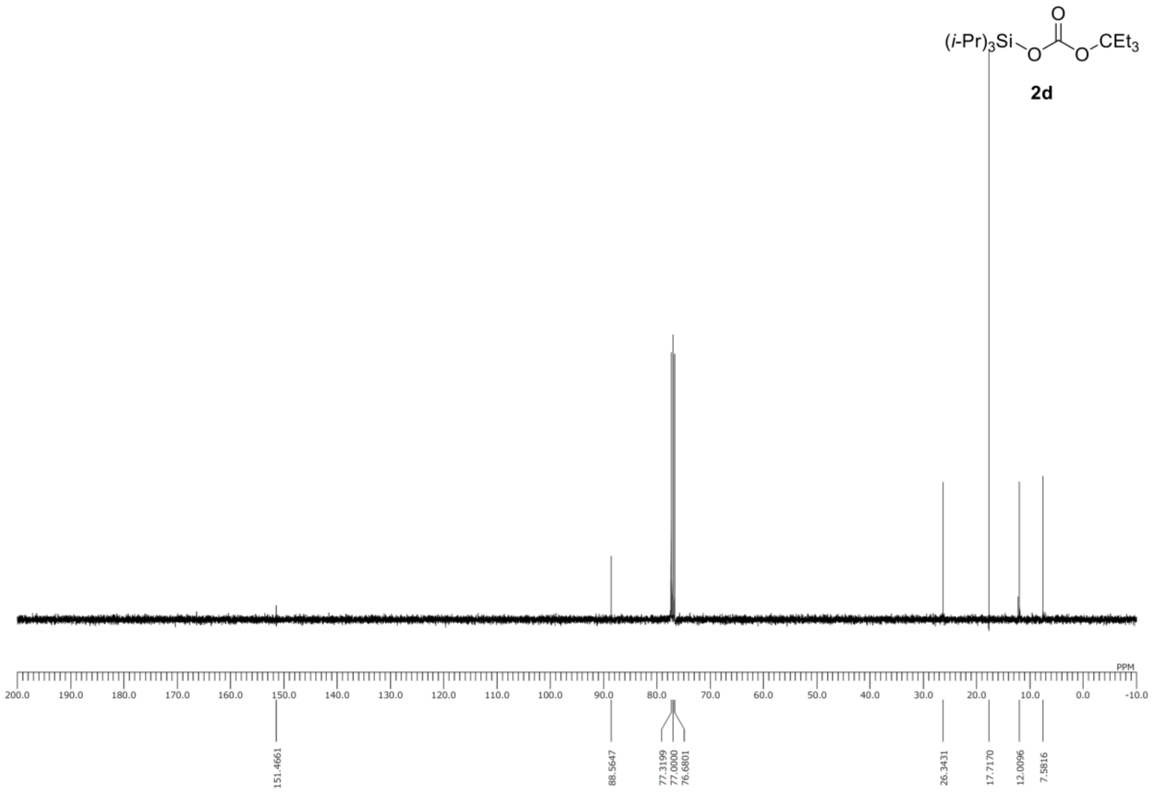

^{1}H NMR spectra of **2d*** ($CDCl_3$, 400 MHz)

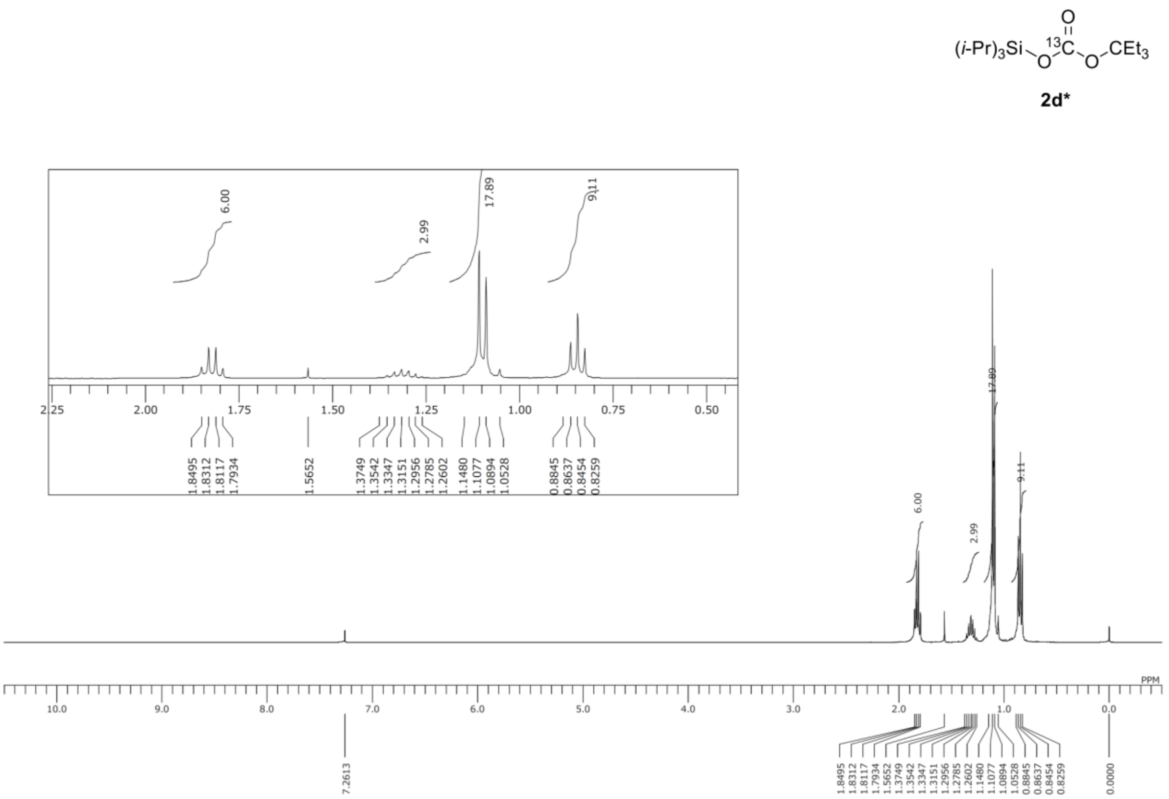

^{13}C NMR spectra of **2d*** ($CDCl_3$, 100 MHz)

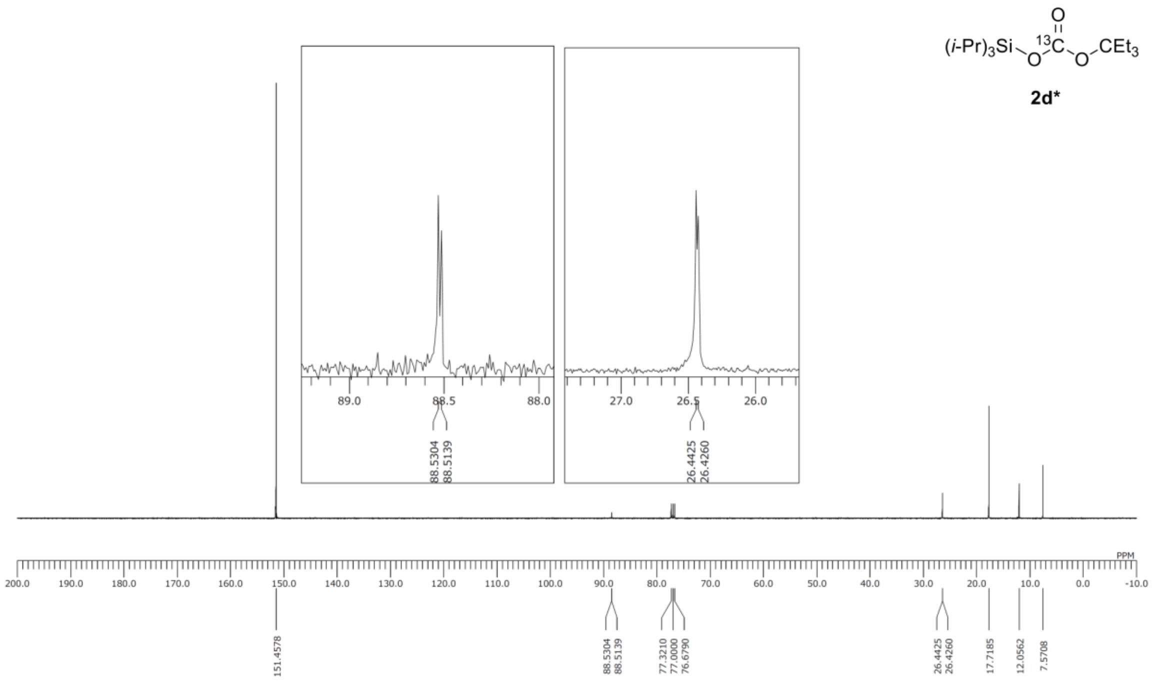

^{1}H NMR spectra of **3a** ($CDCl_3$, 400 MHz)

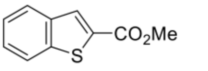

3a

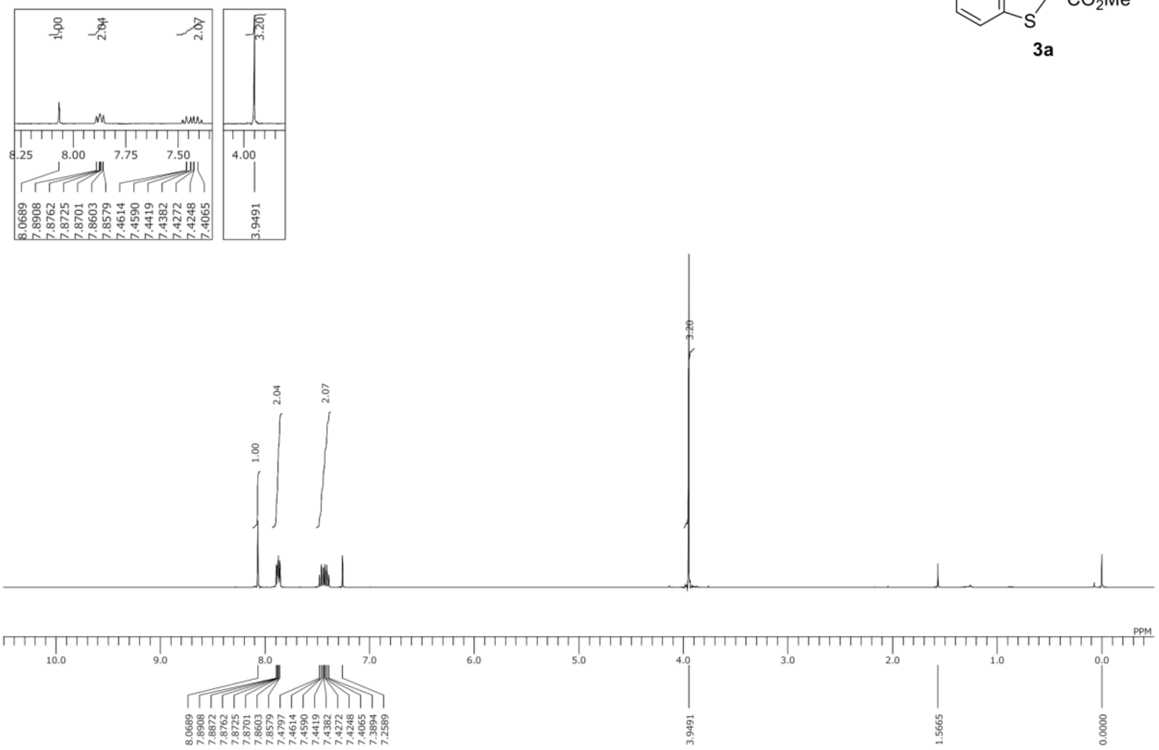

^{13}C NMR spectra of **3a** ($CDCl_3$, 150 MHz)

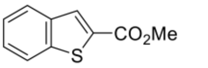

3a

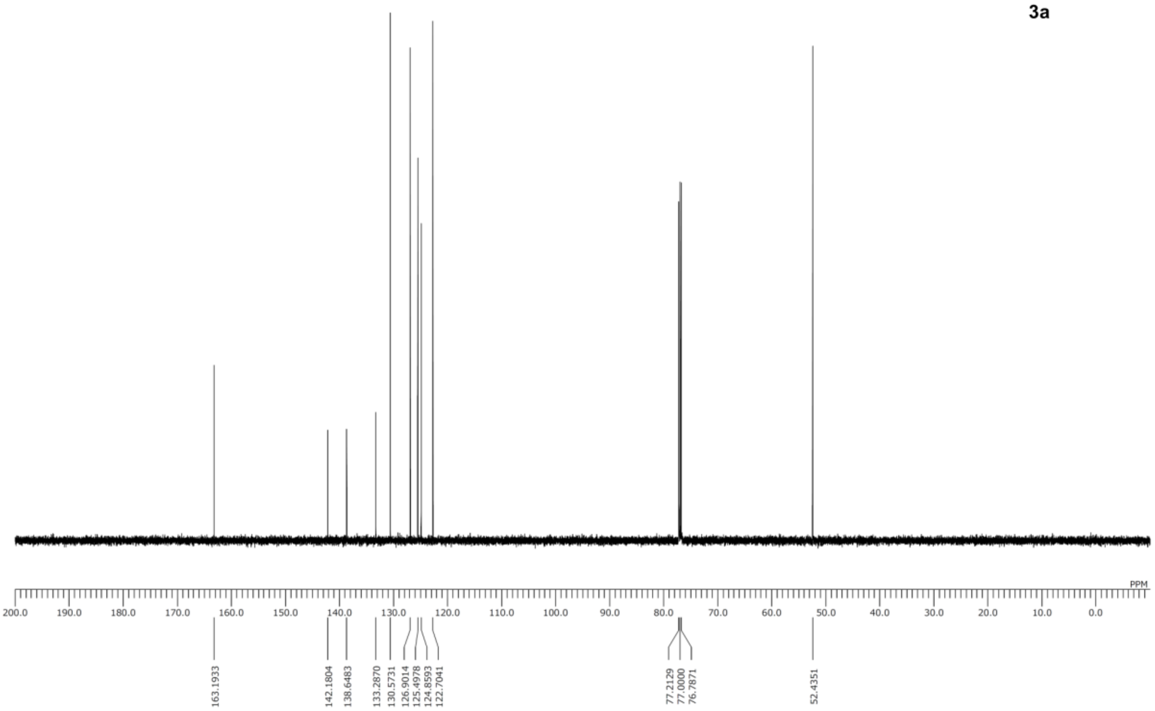

^{1}H NMR spectra of **3b** ($CDCl_3$, 400 MHz)

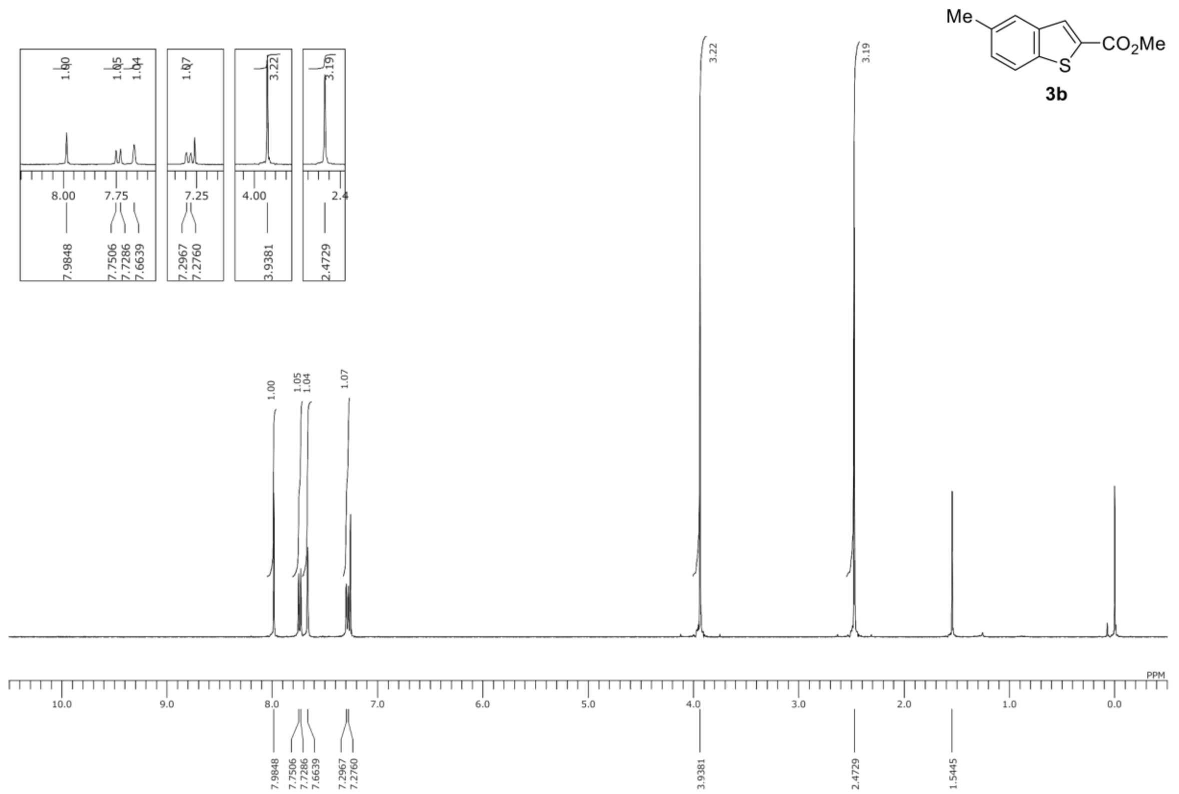

^{13}C NMR spectra of **3b** ($CDCl_3$, 100 MHz)

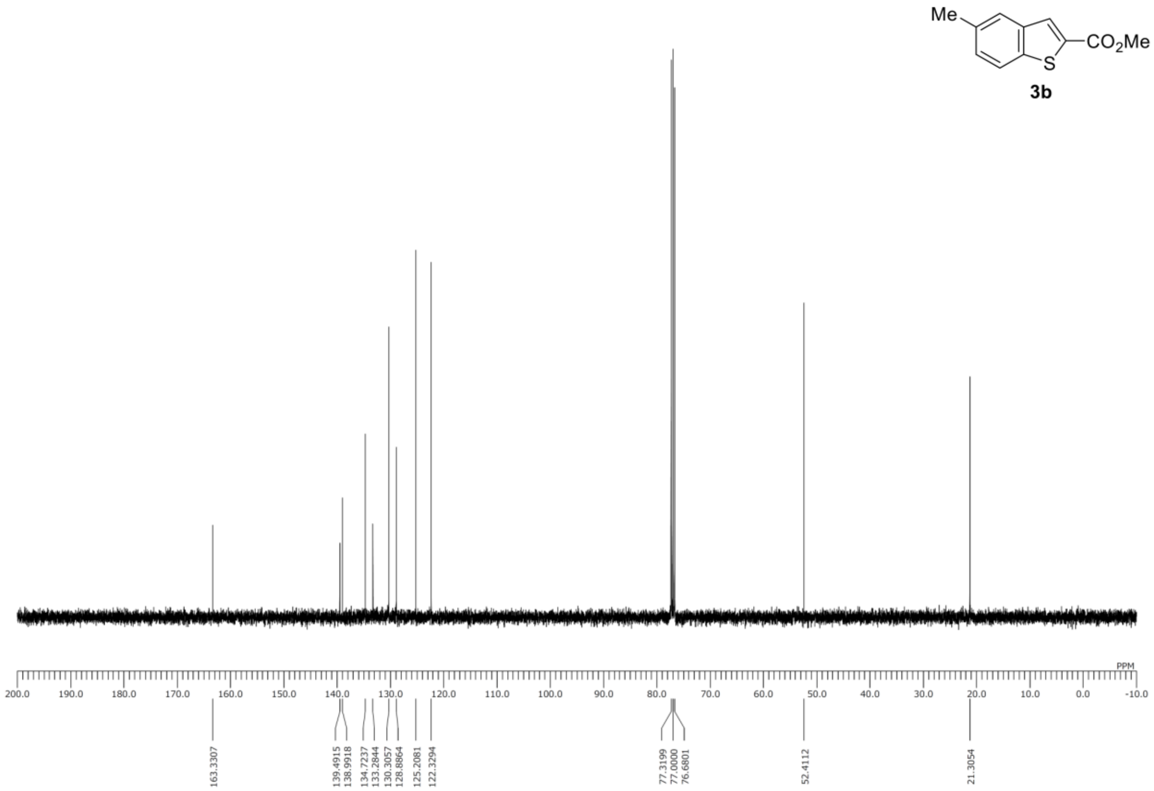

^{1}H NMR spectra of **3c** ($CDCl_3$, 400 MHz)

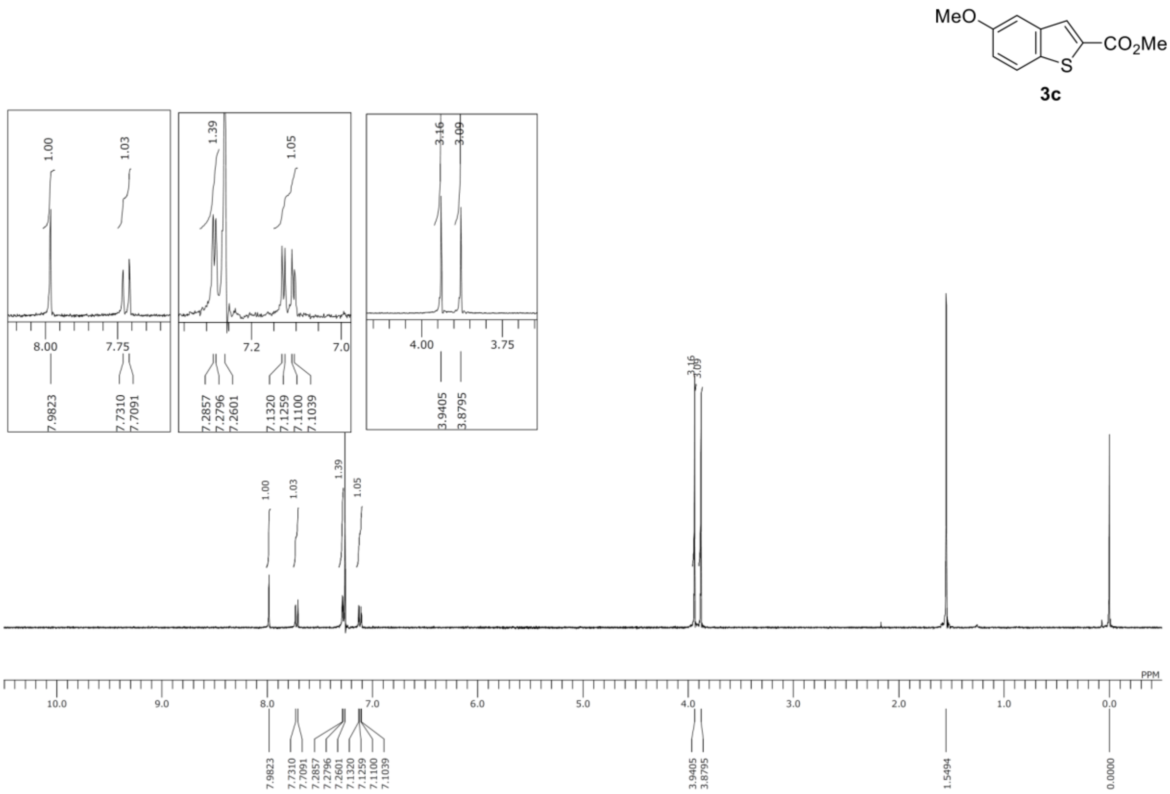

^{13}C NMR spectra of **3c** ($CDCl_3$, 100 MHz)

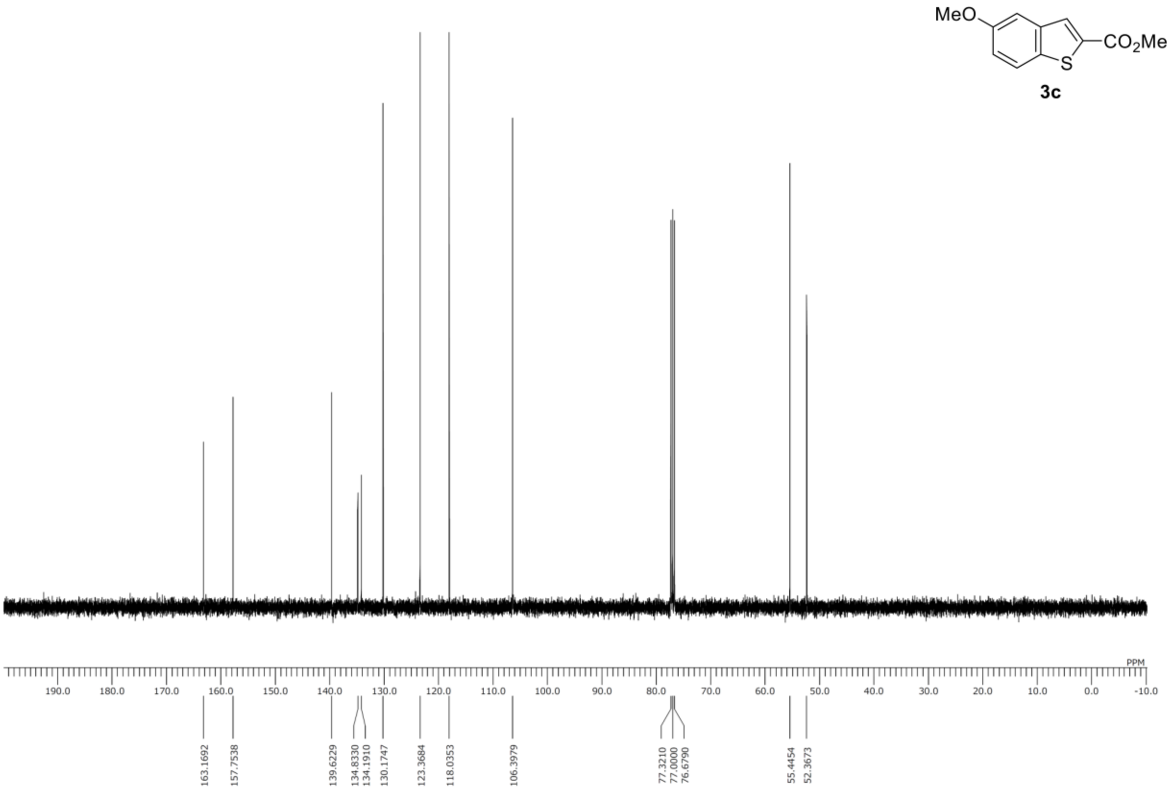

^{1}H NMR spectra of **3d** ($CDCl_3$, 400 MHz)

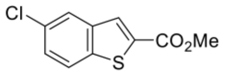

3d

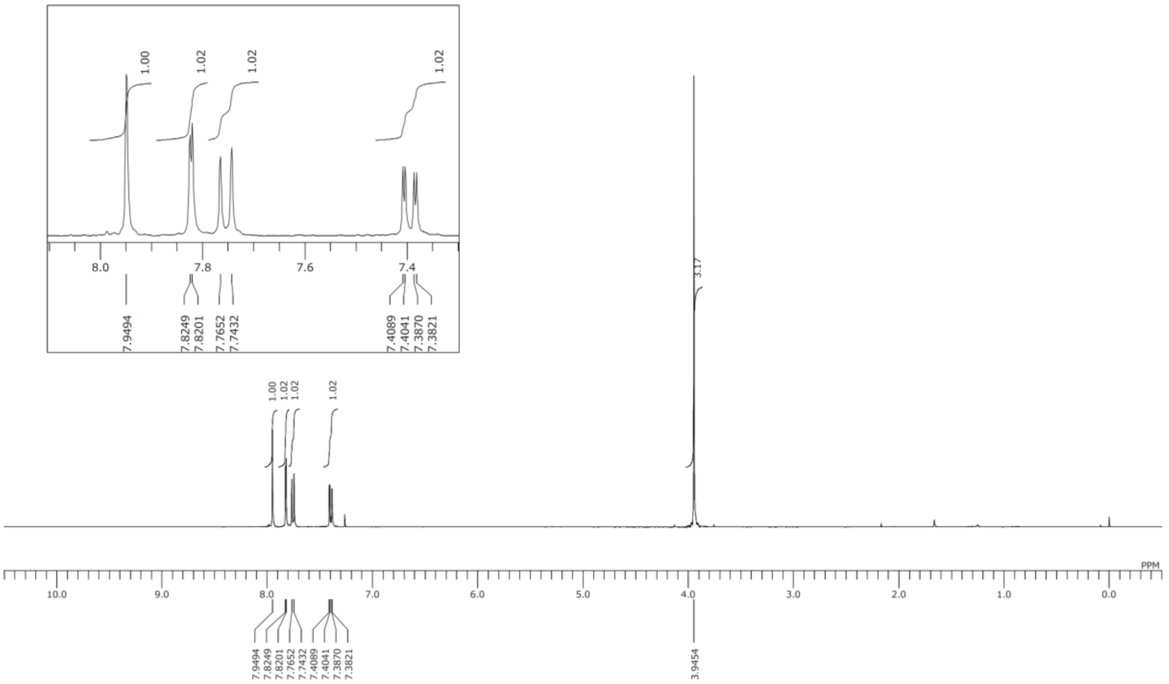

^{13}C NMR spectra of **3d** ($CDCl_3$, 100 MHz)

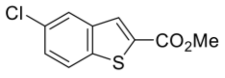

3d

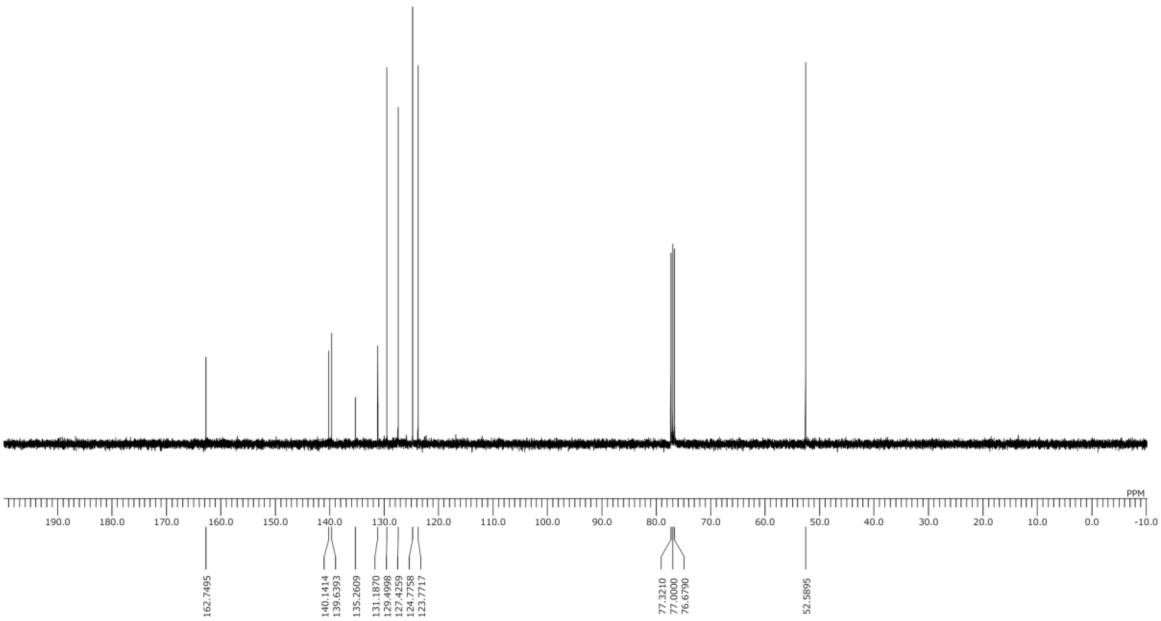

^{1}H NMR spectra of **3e** ($CDCl_3$, 400 MHz)

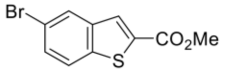

3e

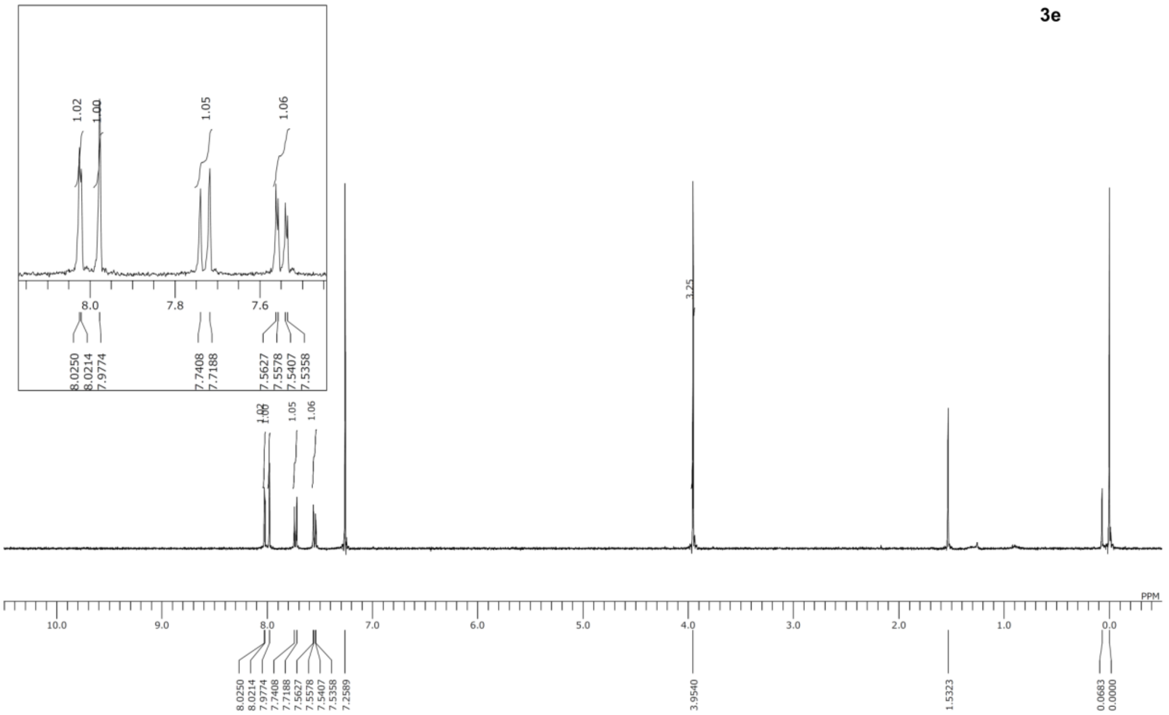

^{13}C NMR spectra of **3e** ($CDCl_3$, 100 MHz)

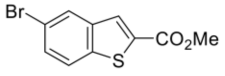

3e

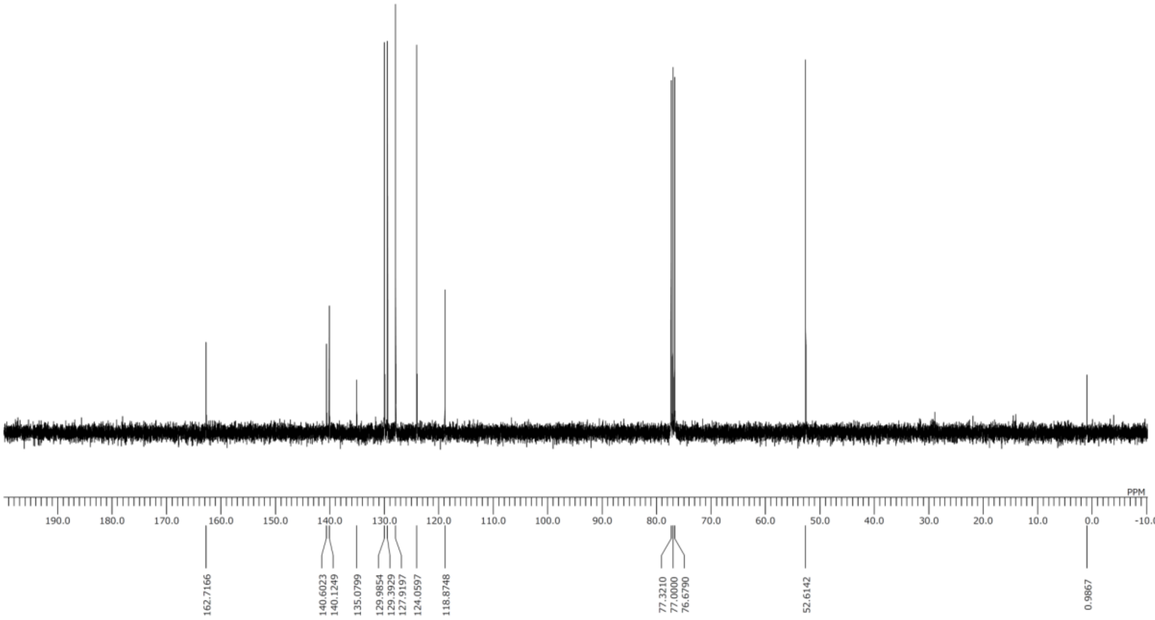

^{1}H NMR spectra of **3f** ($CDCl_3$, 400 MHz)

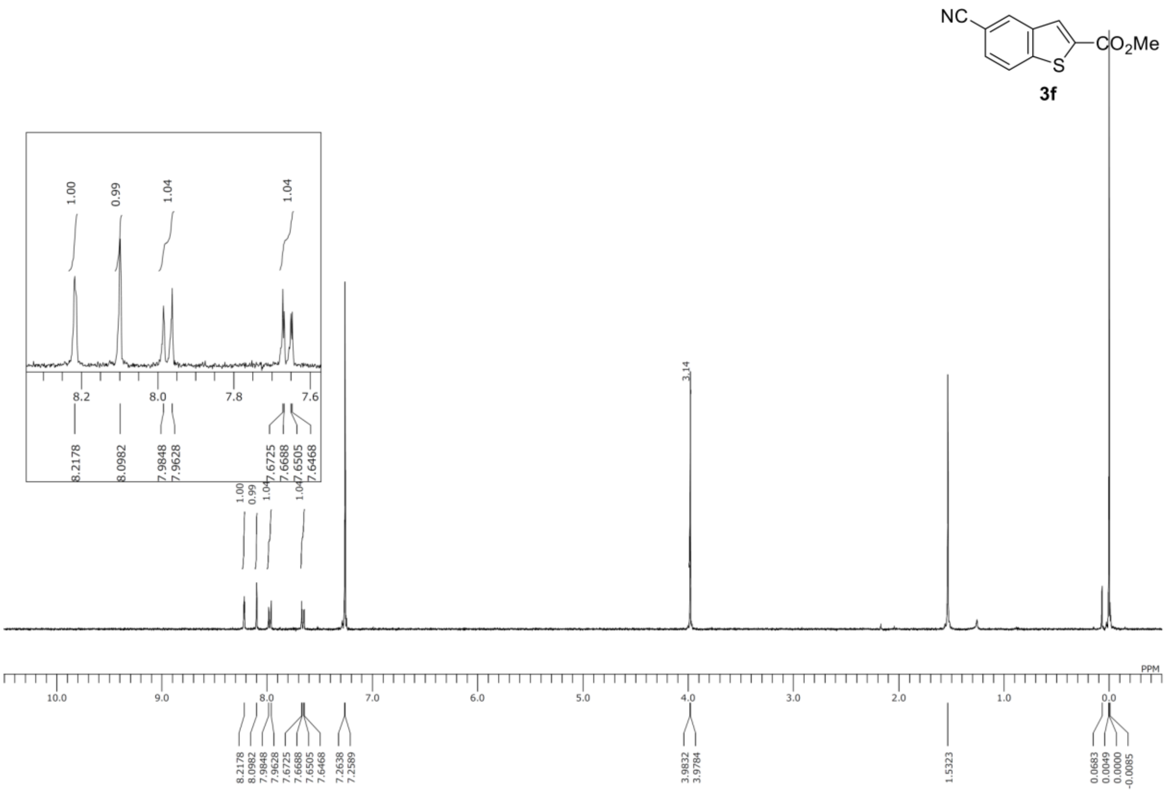

^{13}C NMR spectra of **3f** ($CDCl_3$, 100 MHz)

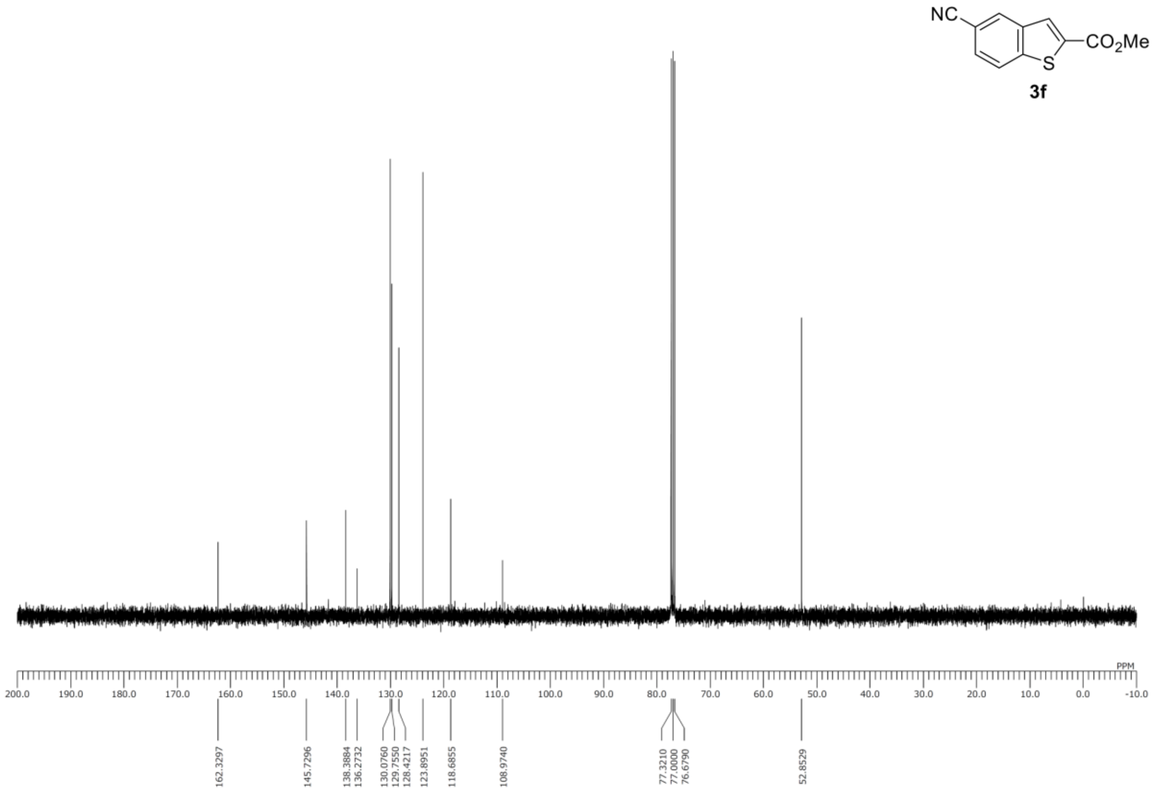

^{1}H NMR spectra of **3g** ($CDCl_3$, 400 MHz)

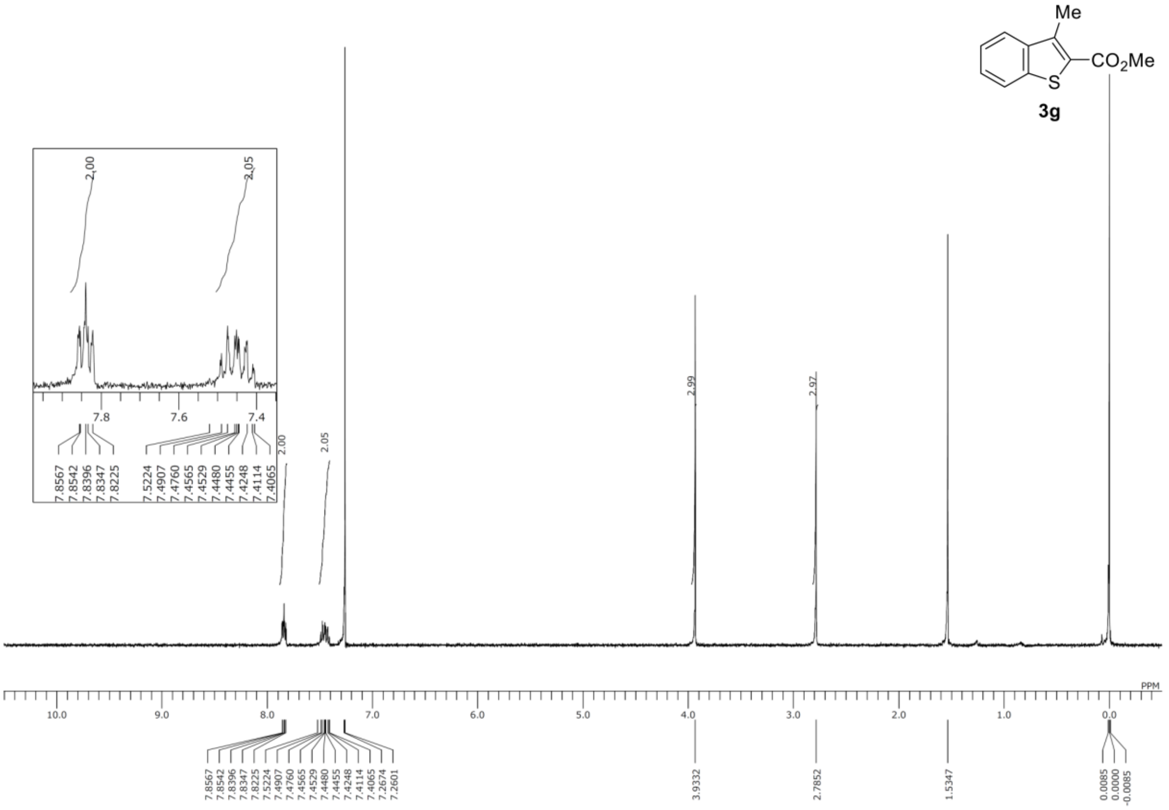

^{13}C NMR spectra of **3g** ($CDCl_3$, 100 MHz)

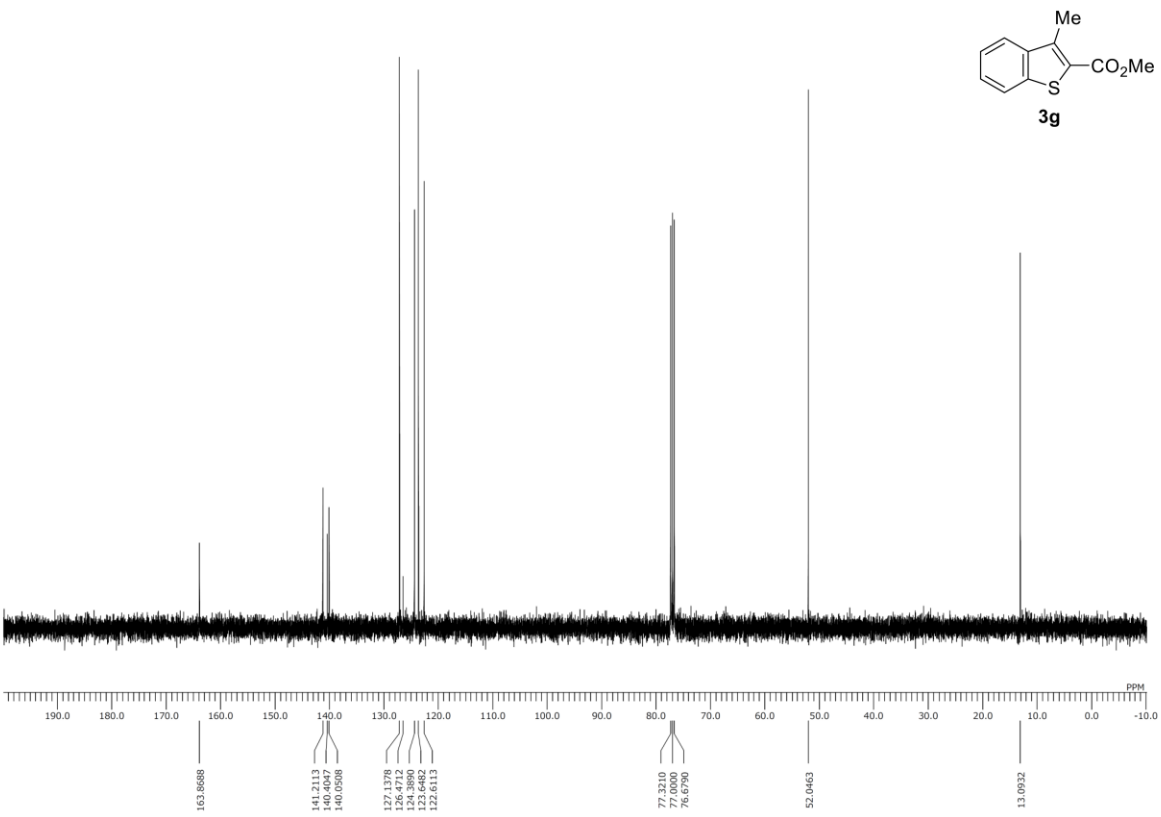

^{1}H NMR spectra of **3h** ($CDCl_3$, 400 MHz)

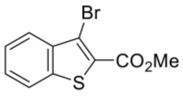

3h

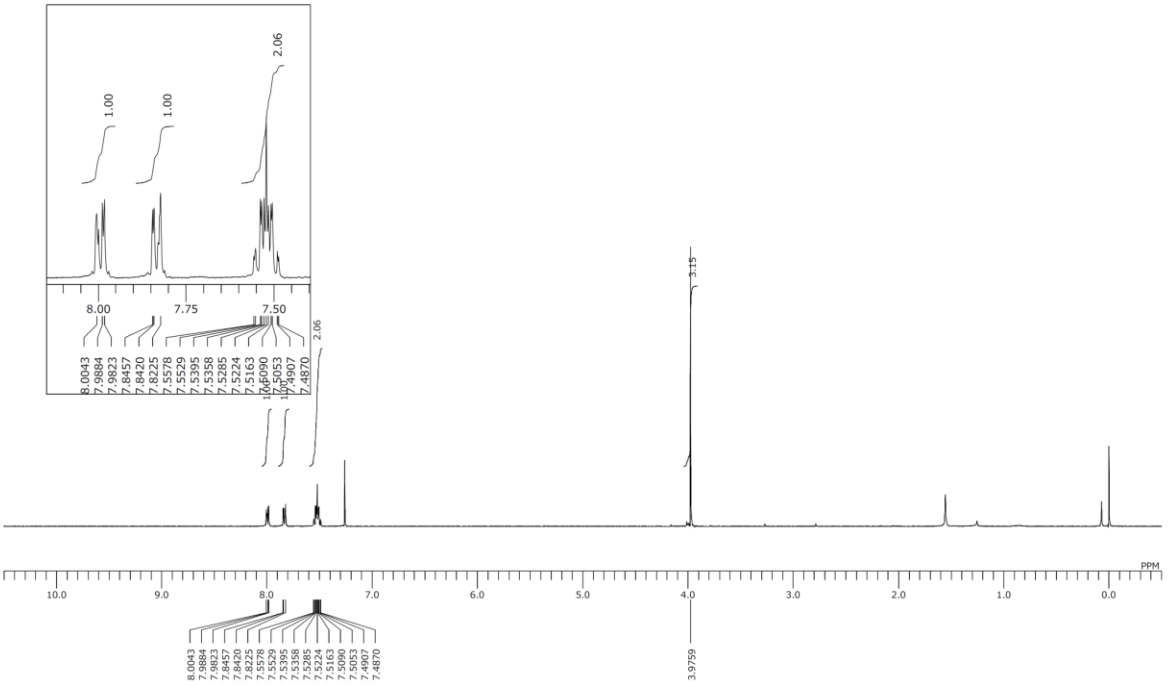

^{13}C NMR spectra of **3h** ($CDCl_3$, 100 MHz)

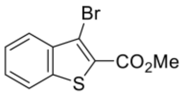

3h

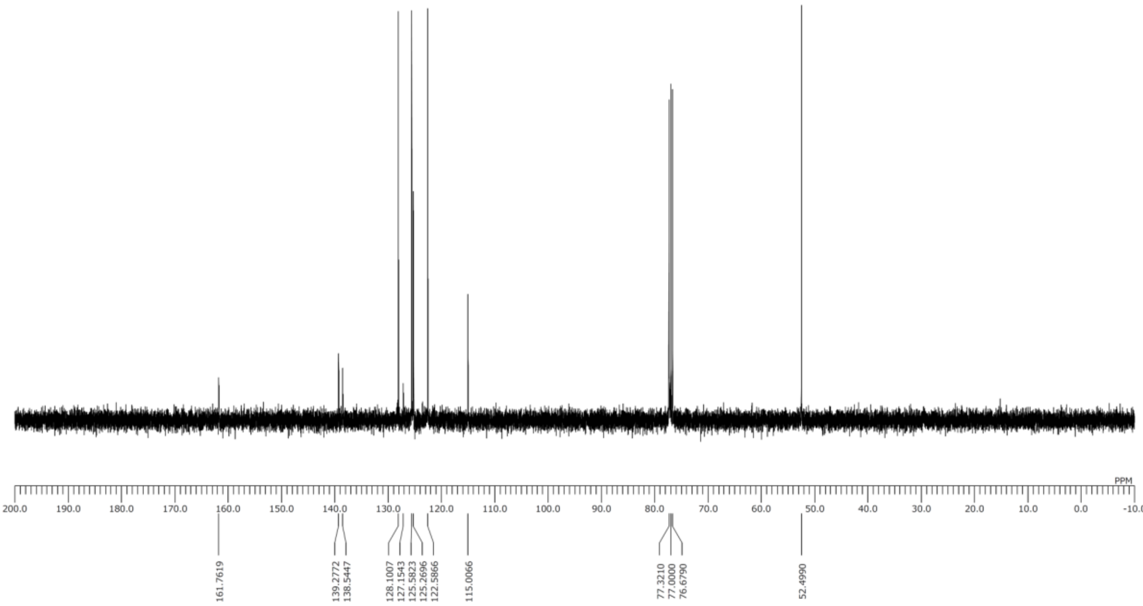

^{1}H NMR spectra of **3i** ($CDCl_3$, 400 MHz)

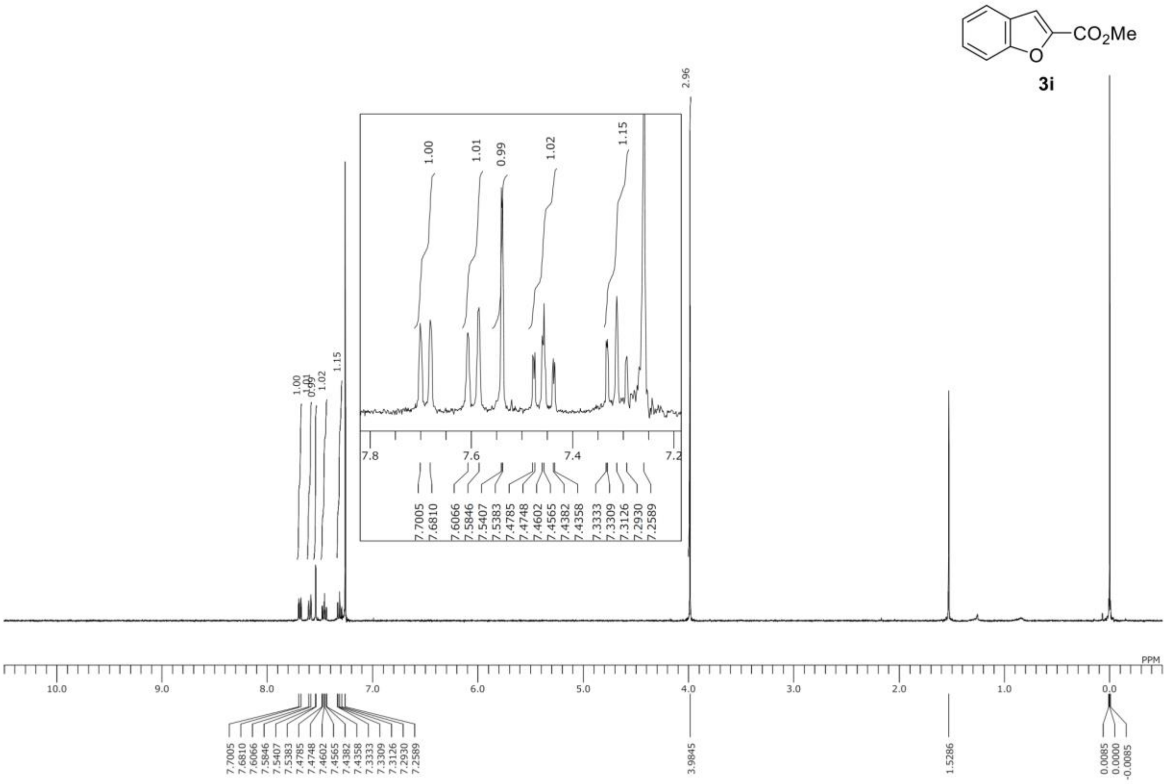

^{13}C NMR spectra of **3i** ($CDCl_3$, 150 MHz)

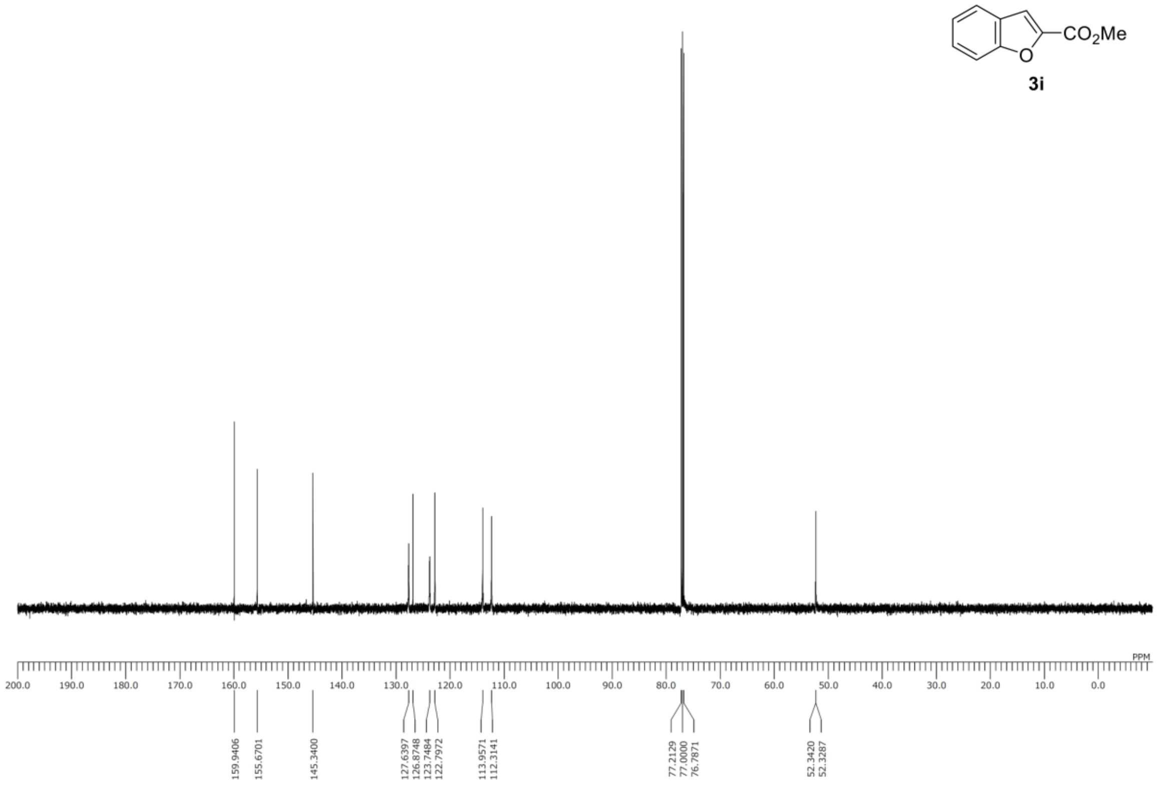

^1H NMR spectra of **3j** ($CDCl_3$, 400 MHz)

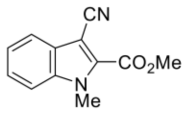

3j

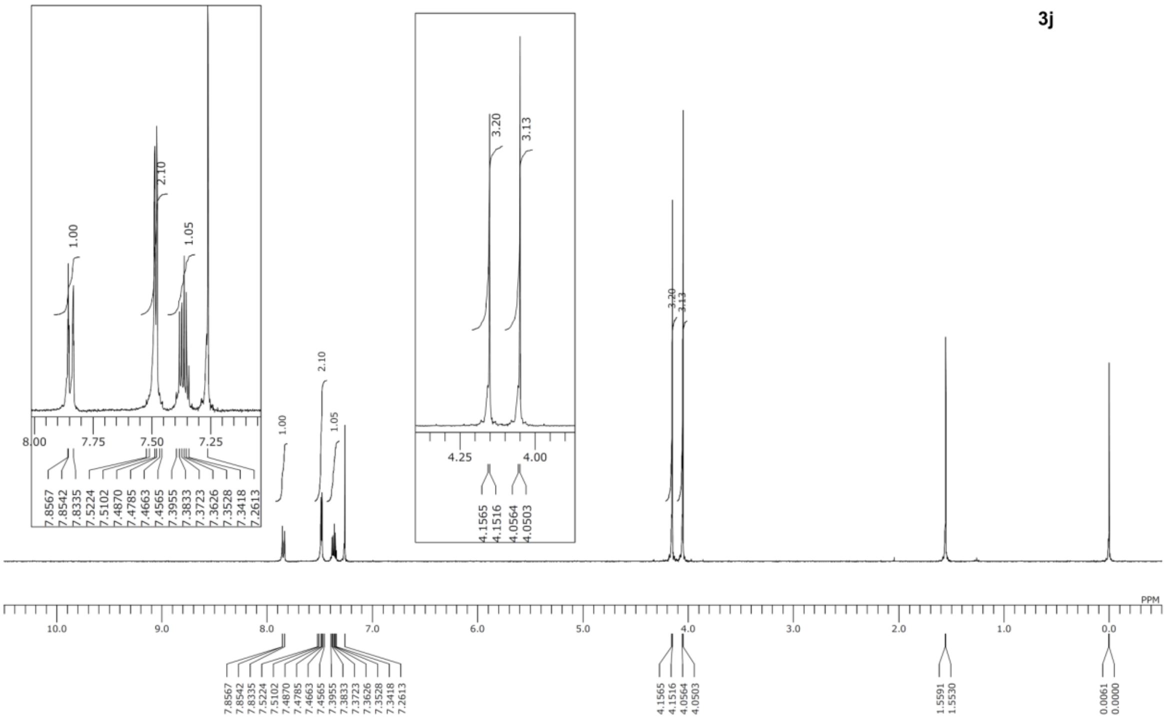

^{13}C NMR spectra of **3j** ($CDCl_3$, 100 MHz)

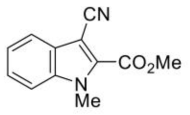

3j

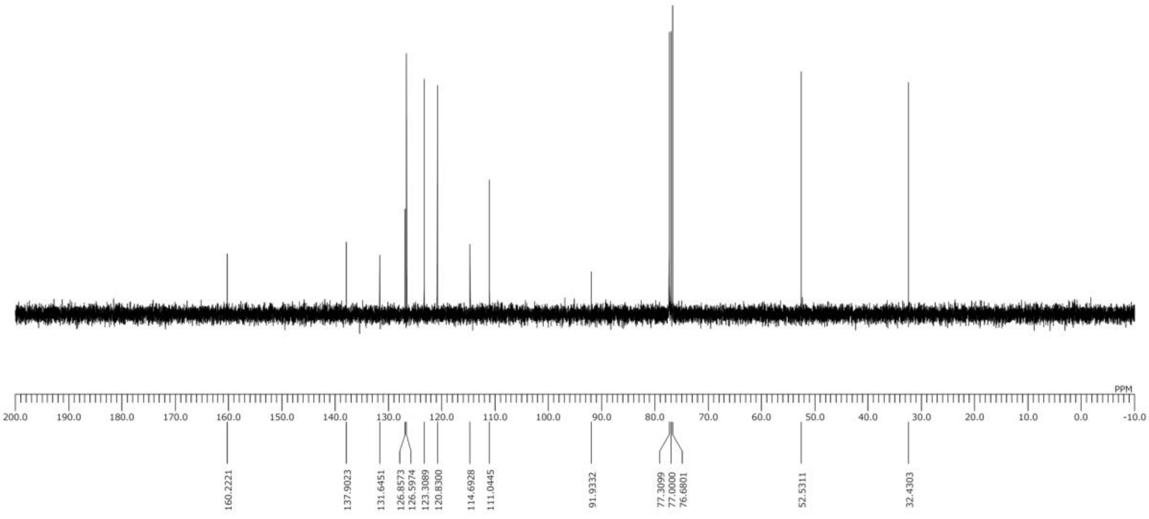

^{1}H NMR spectra of **3k** ($CDCl_3$, 400 MHz)

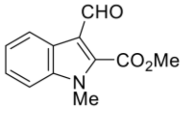

3k

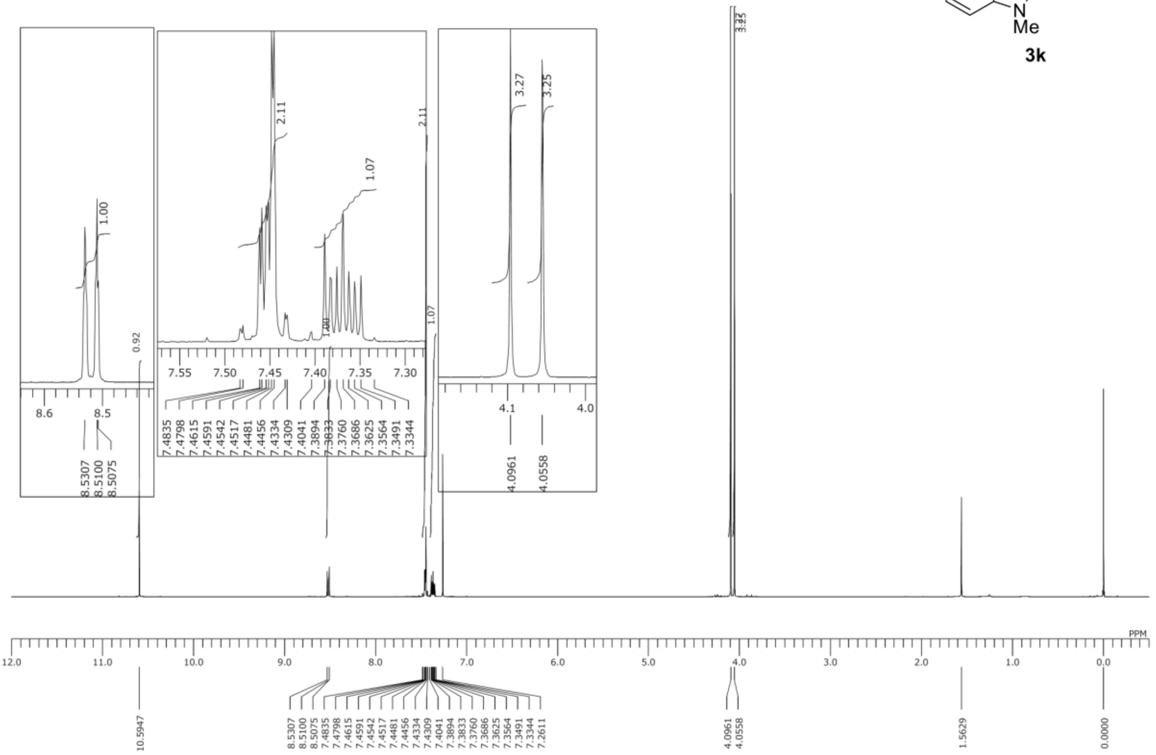

^{13}C NMR spectra of **3k** ($CDCl_3$, 100 MHz)

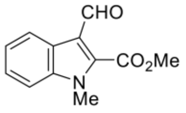

3k

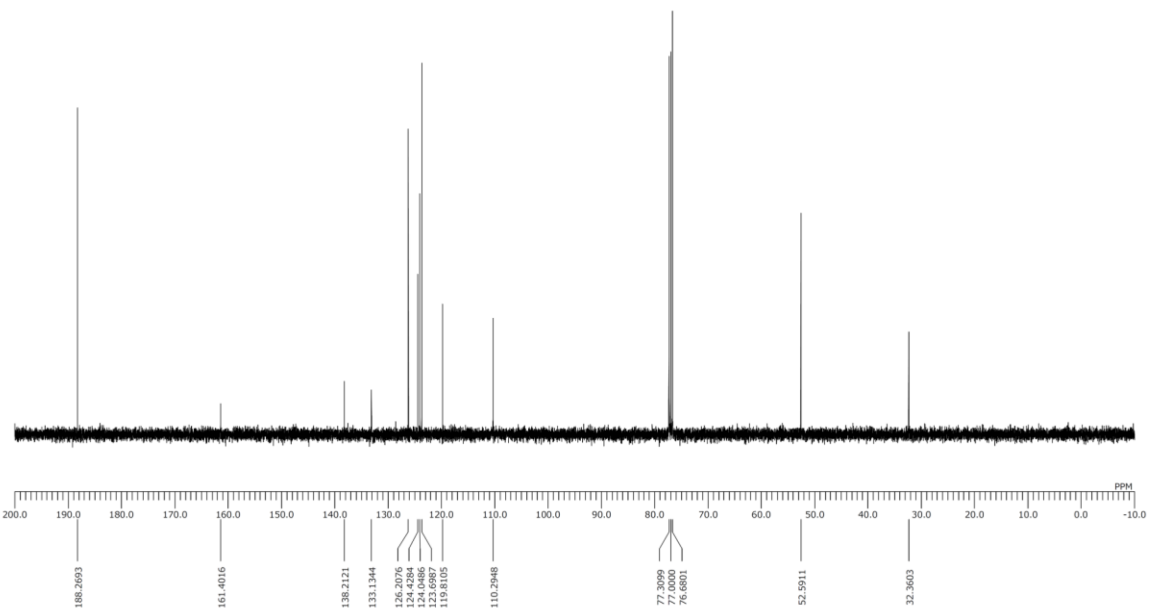

^{1}H NMR spectra of **3l** ($CDCl_3$, 400 MHz)

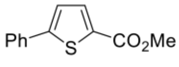

3l

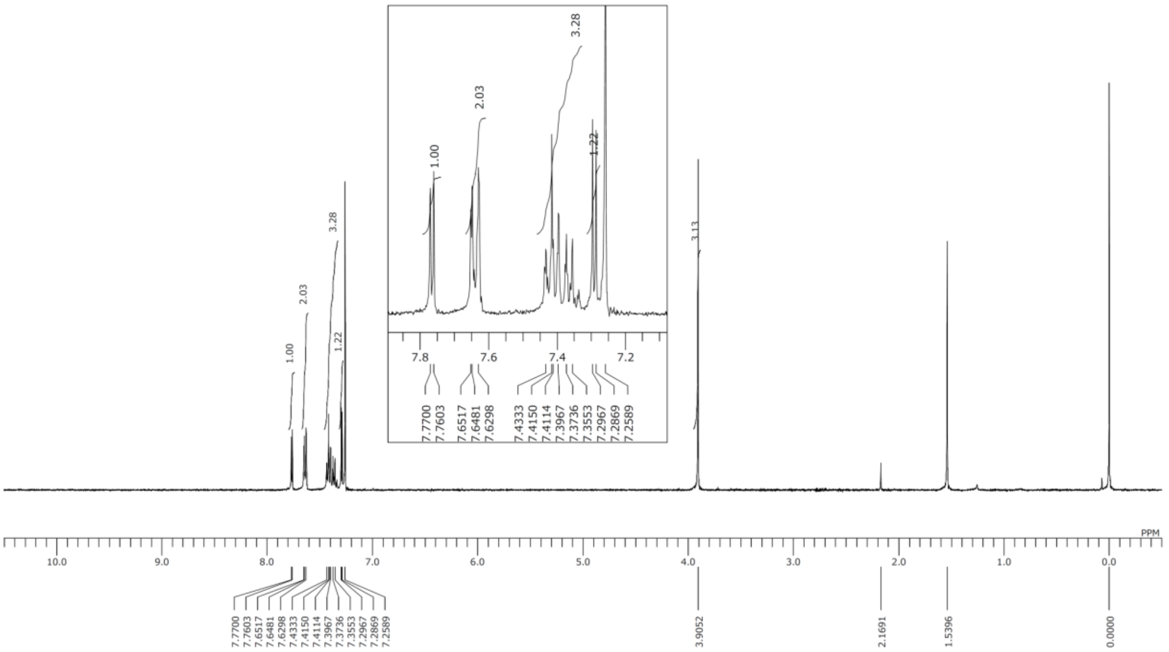

^{13}C NMR spectra of **3l** ($CDCl_3$, 100 MHz)

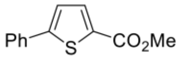

3l

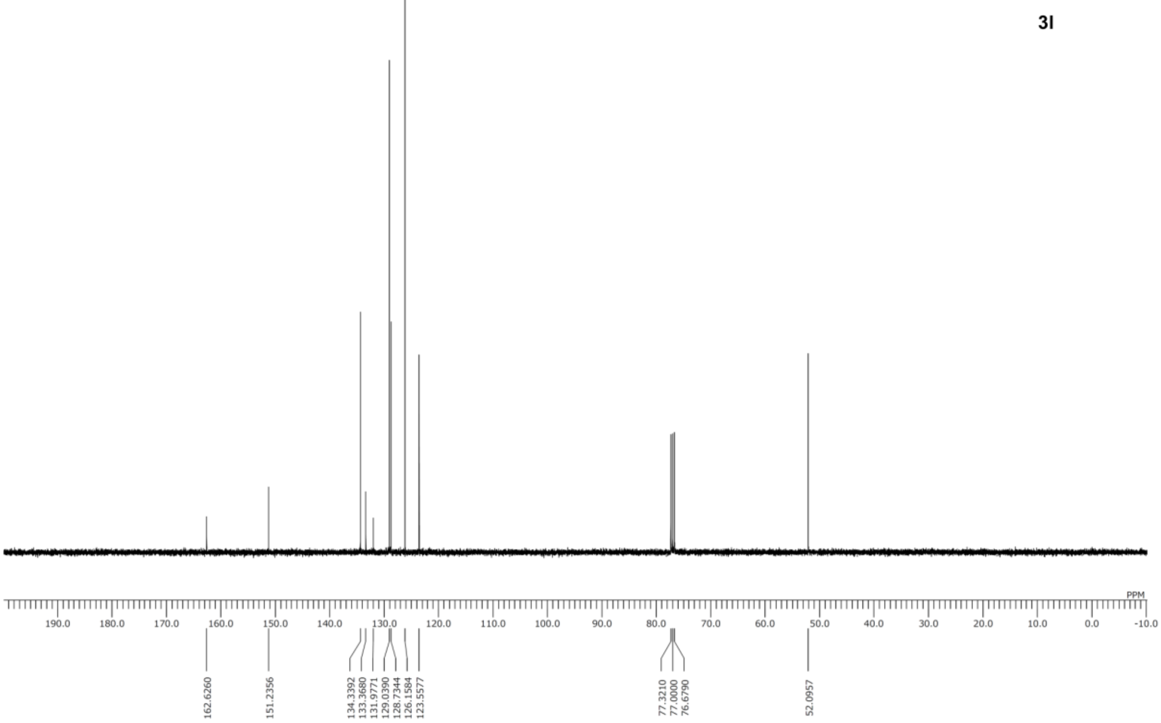

^{1}H NMR spectra of **3m** ($CDCl_3$, 400 MHz)

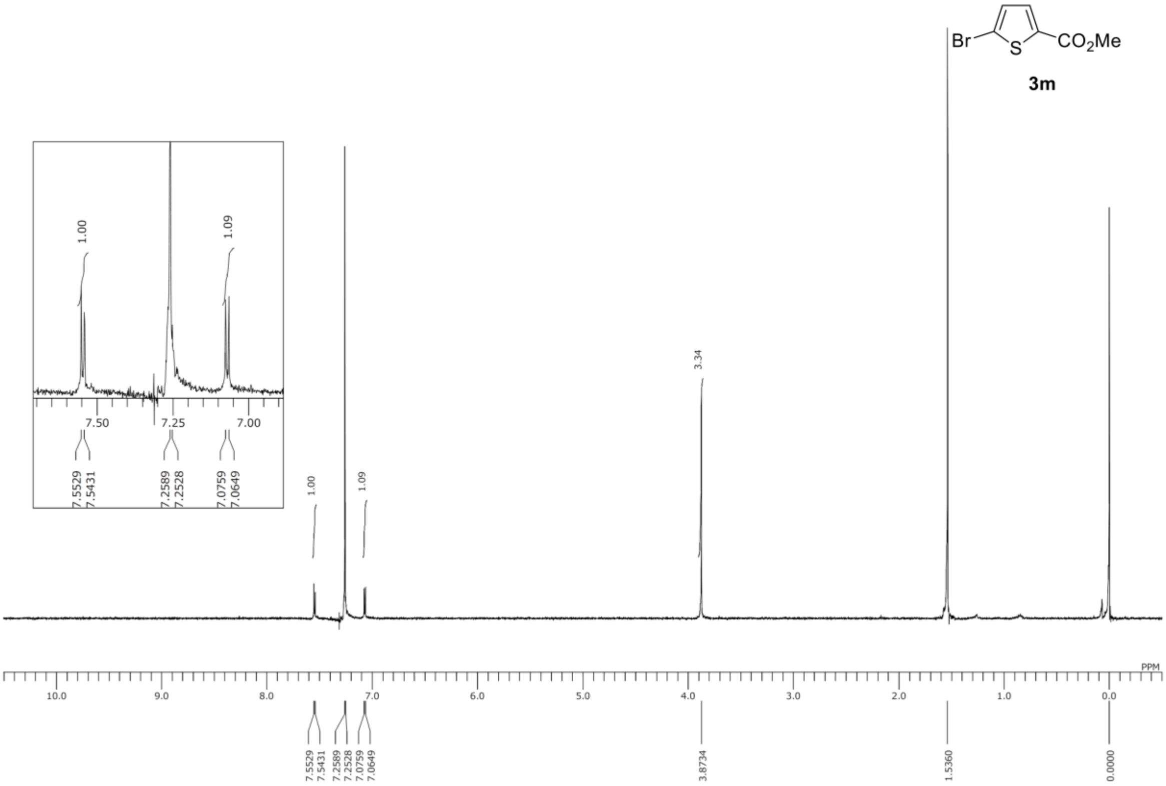

^{13}C NMR spectra of **3m** ($CDCl_3$, 100 MHz)

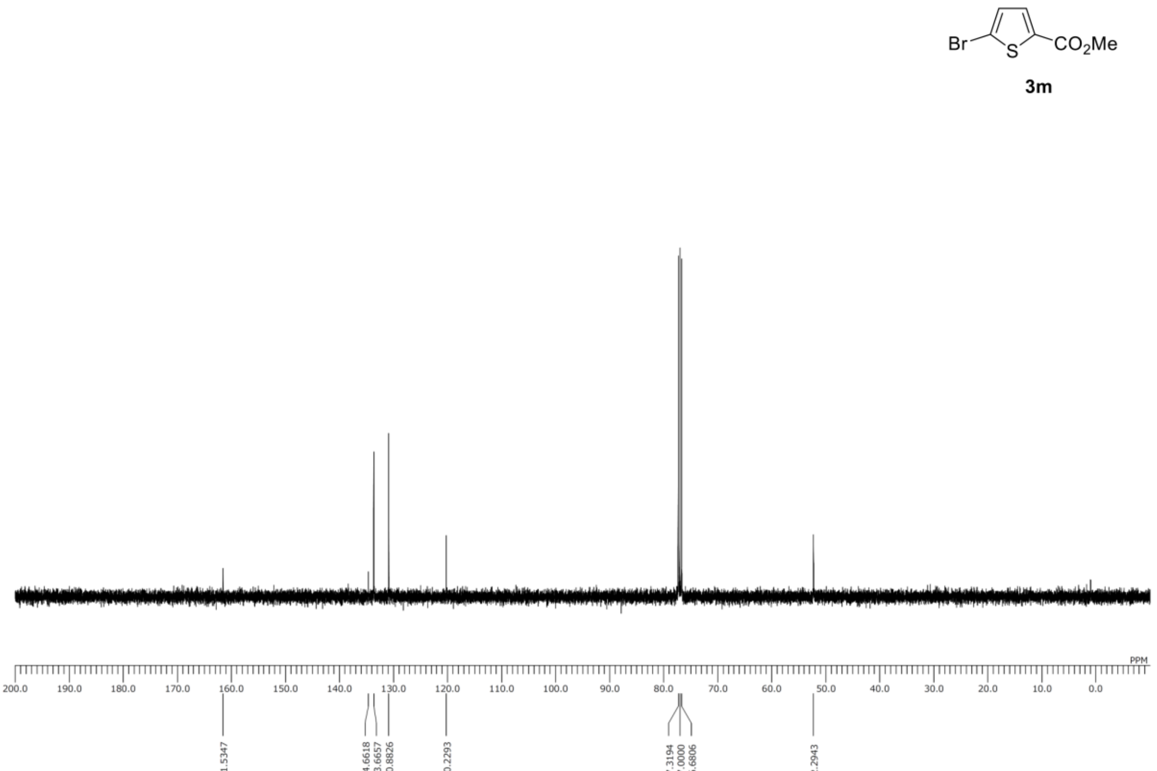

^{1}H NMR spectra of **3n** ($CDCl_3$, 400 MHz)

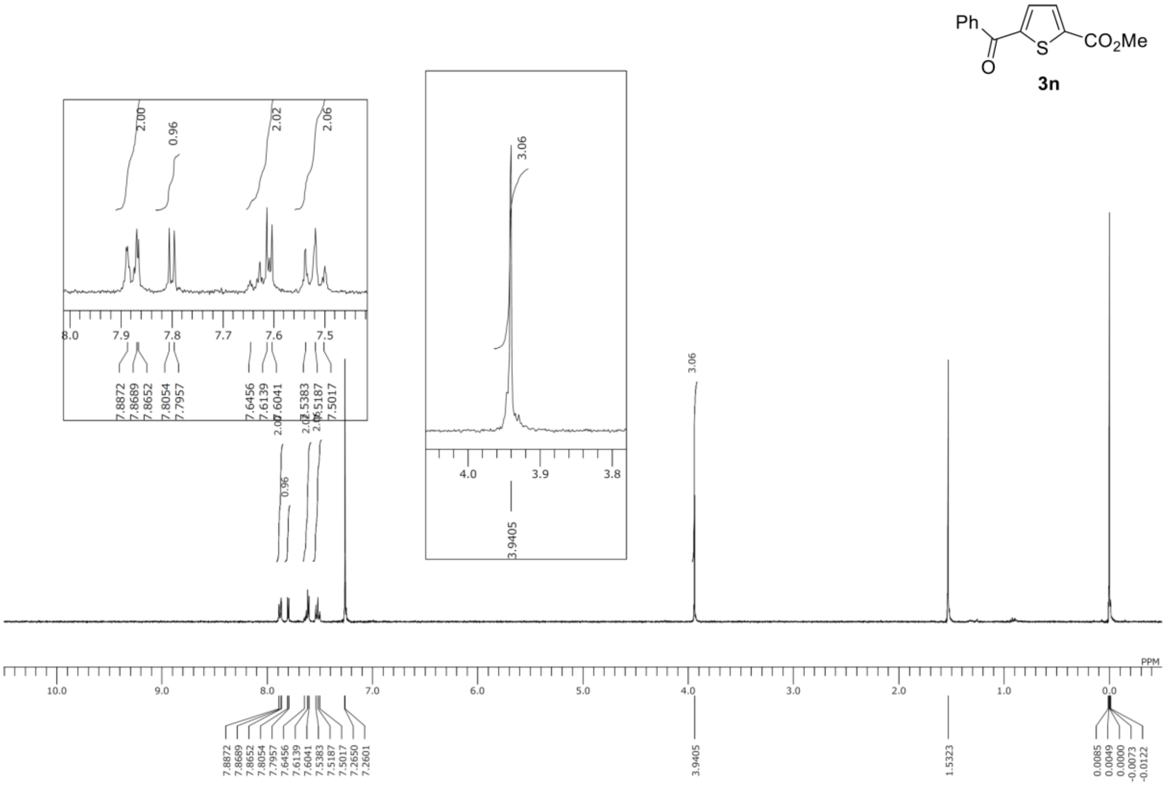

^{13}C NMR spectra of **3n** ($CDCl_3$, 100 MHz)

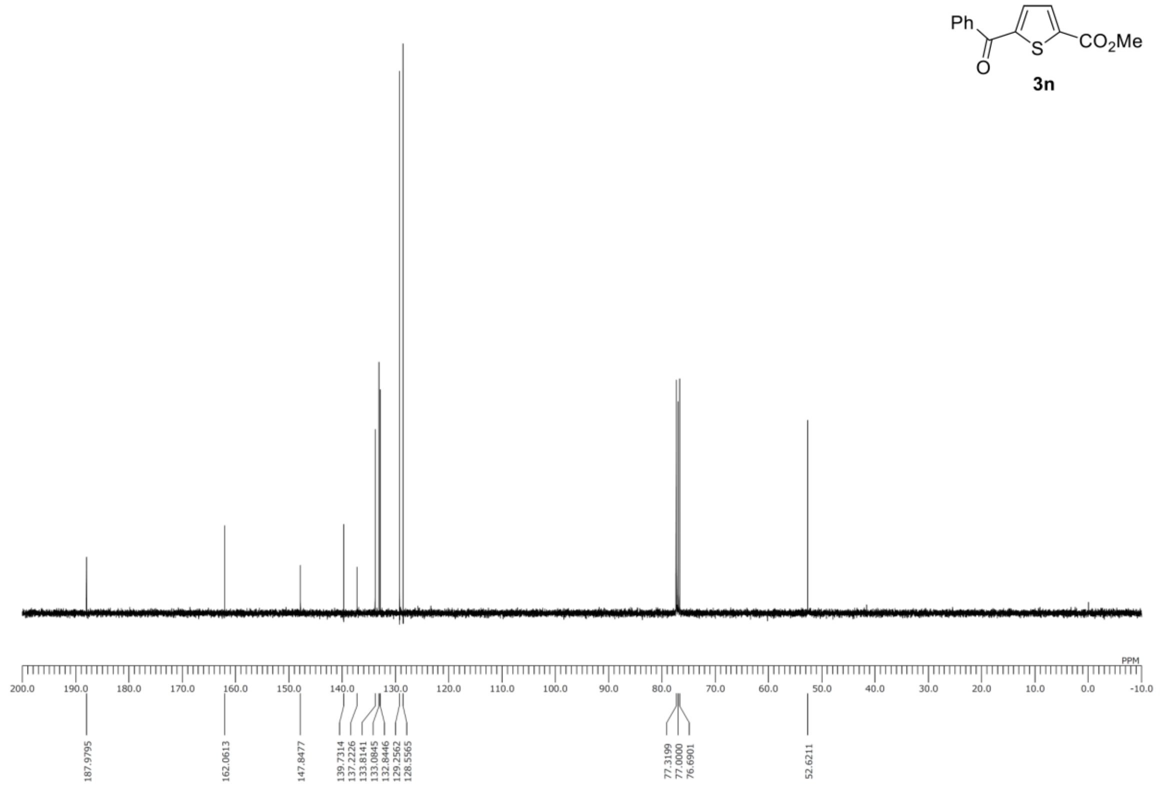

^{1}H NMR spectra of **3o** ($CDCl_3$, 400 MHz)

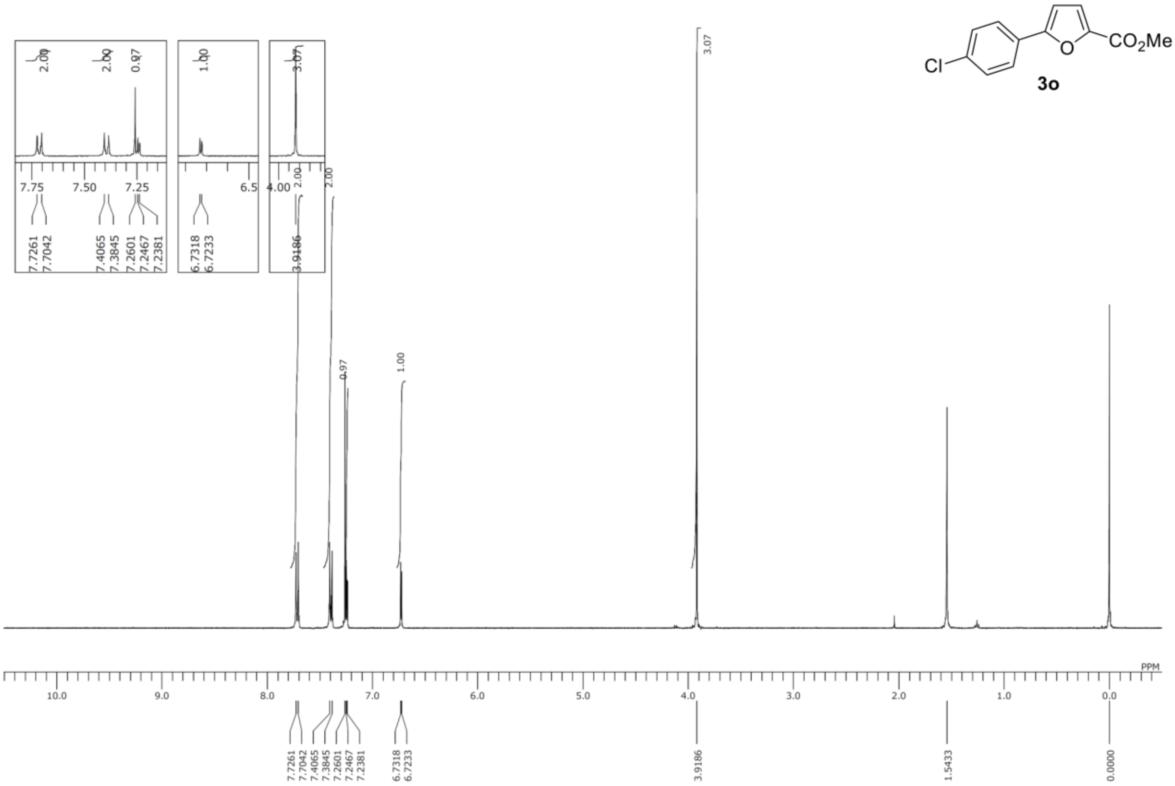

^{13}C NMR spectra of **3o** ($CDCl_3$, 100 MHz)

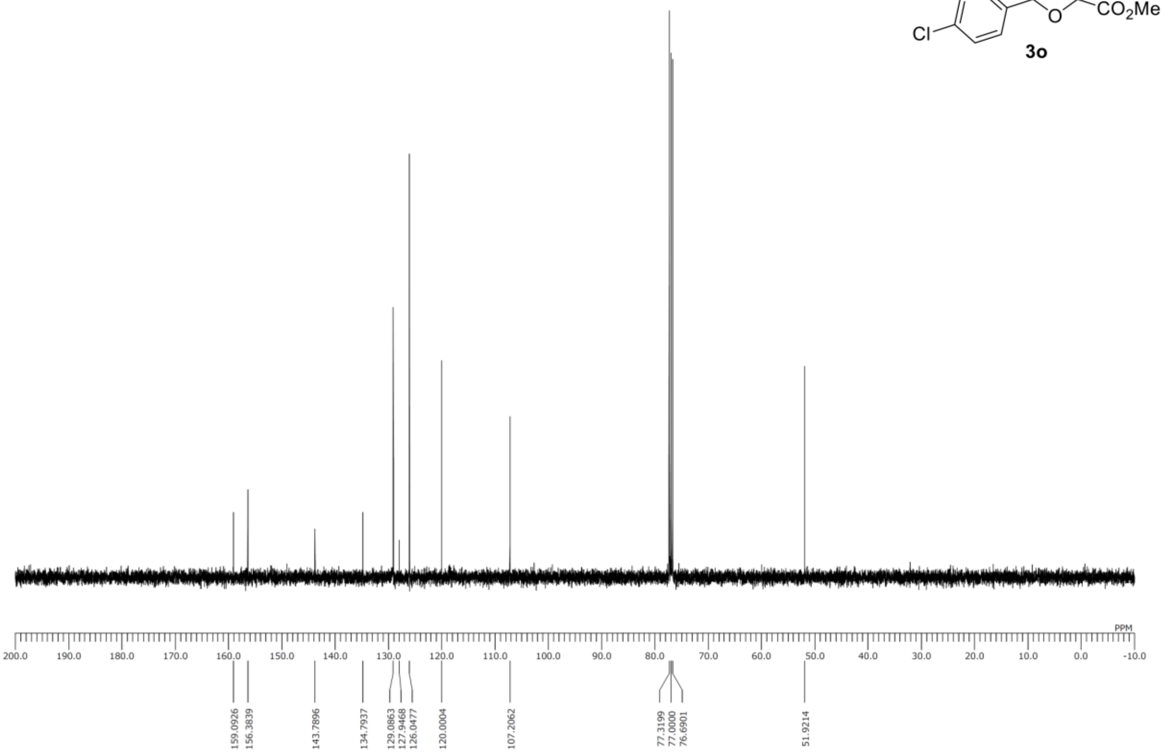

^{1}H NMR spectra of **5a** ($CDCl_3$, 400 MHz)

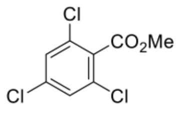

5a

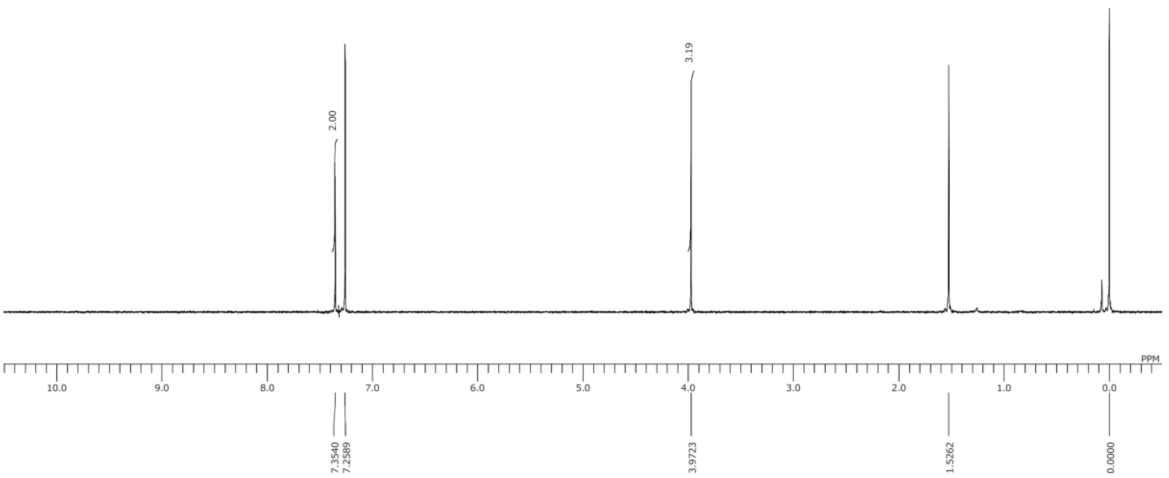

^{13}C NMR spectra of **5a** ($CDCl_3$, 100 MHz)

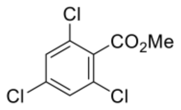

5a

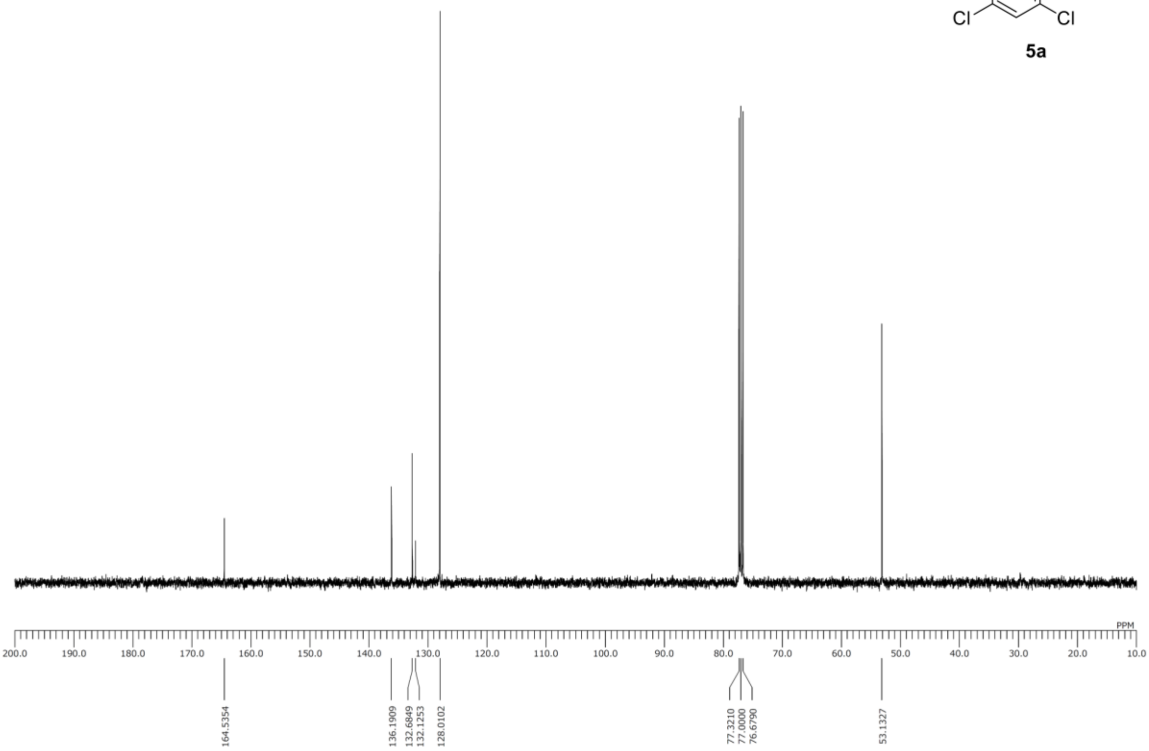

^{1}H NMR spectra of **5b** ($CDCl_3$, 400 MHz)

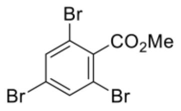

5b

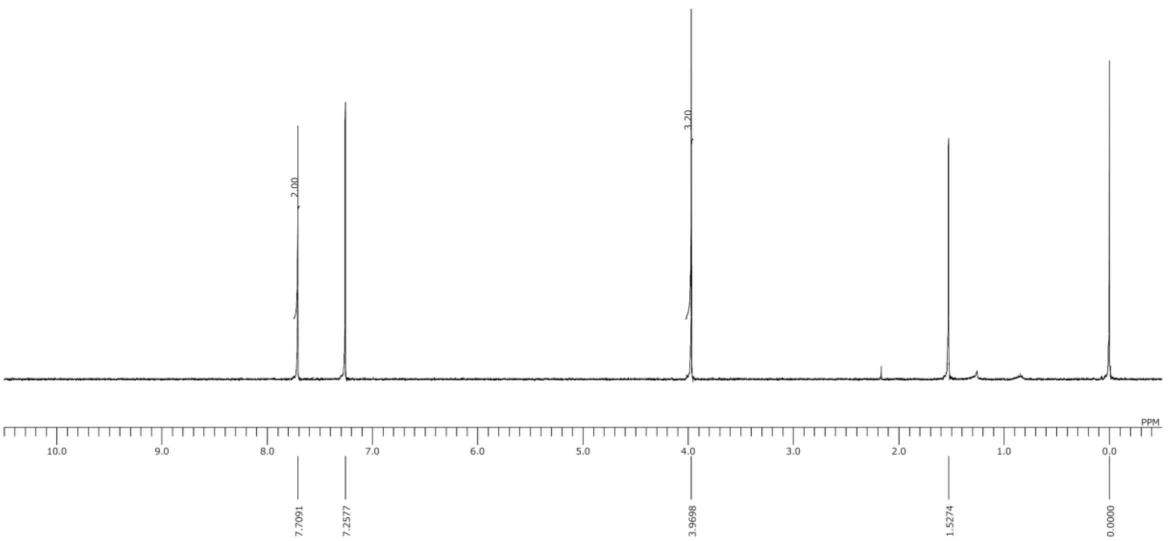

^{13}C NMR spectra of **5b** ($CDCl_3$, 100 MHz)

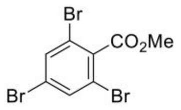

5b

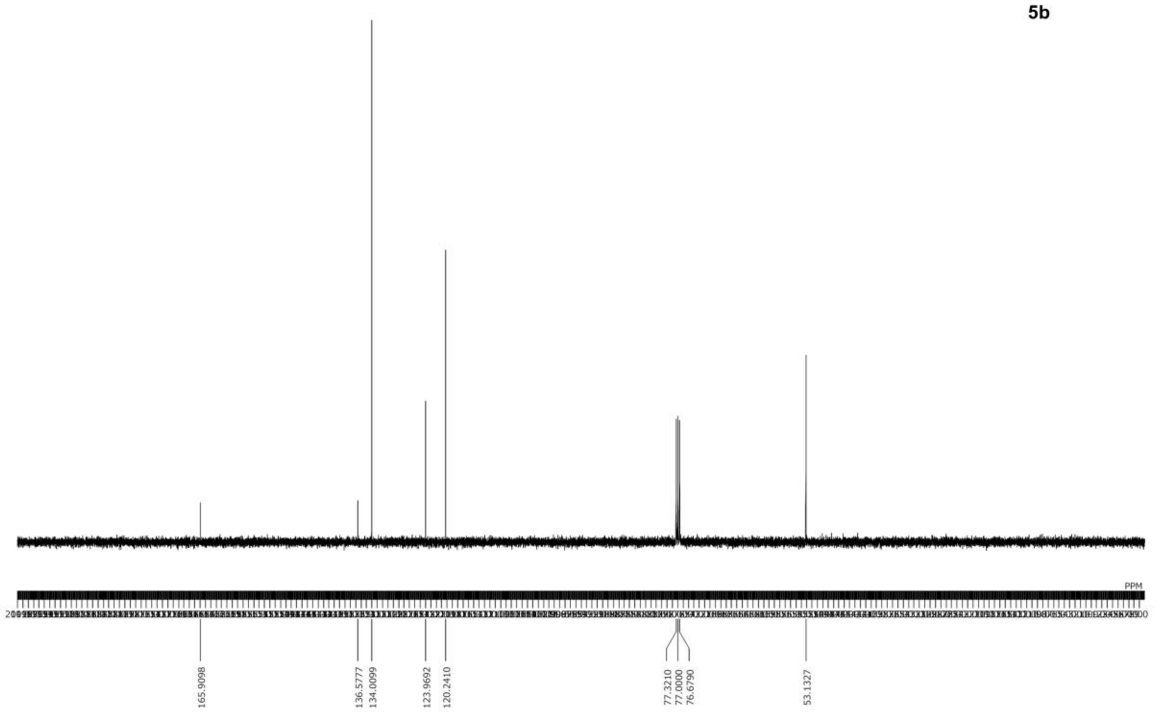

^{1}H NMR spectra of **5c** ($CDCl_3$, 400 MHz)

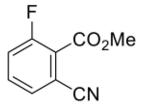

5c

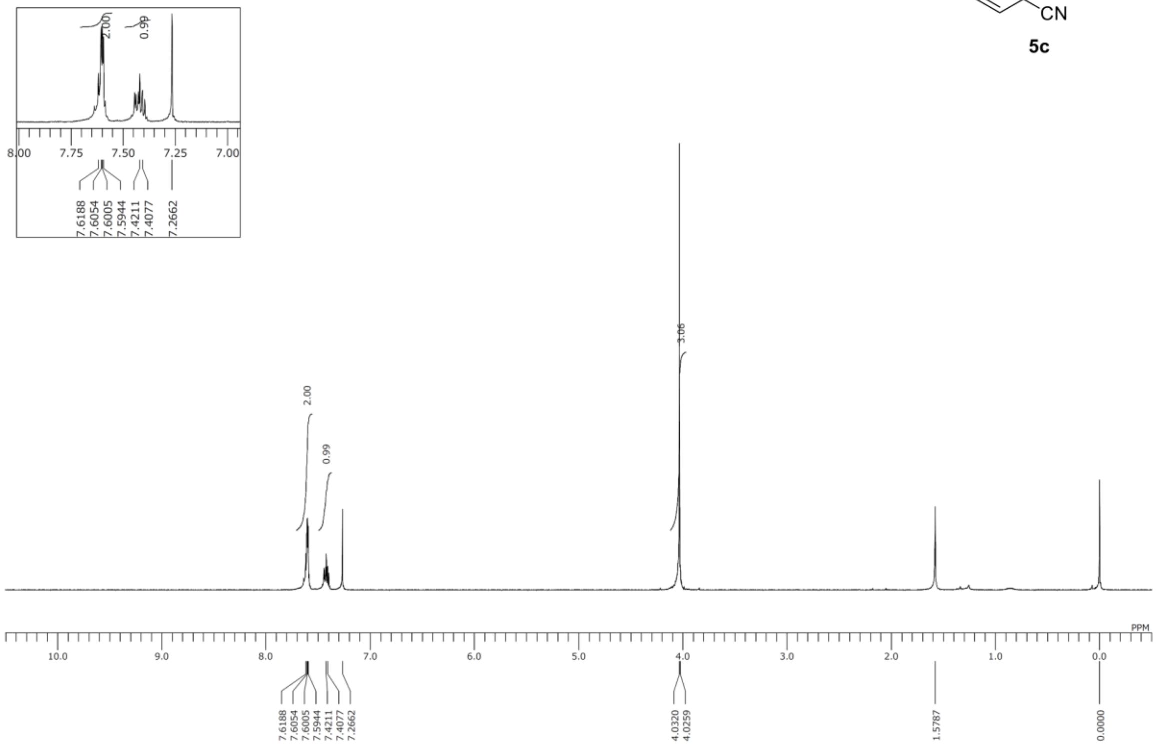

^{13}C NMR spectra of **5c** ($CDCl_3$, 100 MHz)

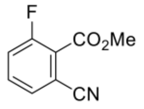

5c

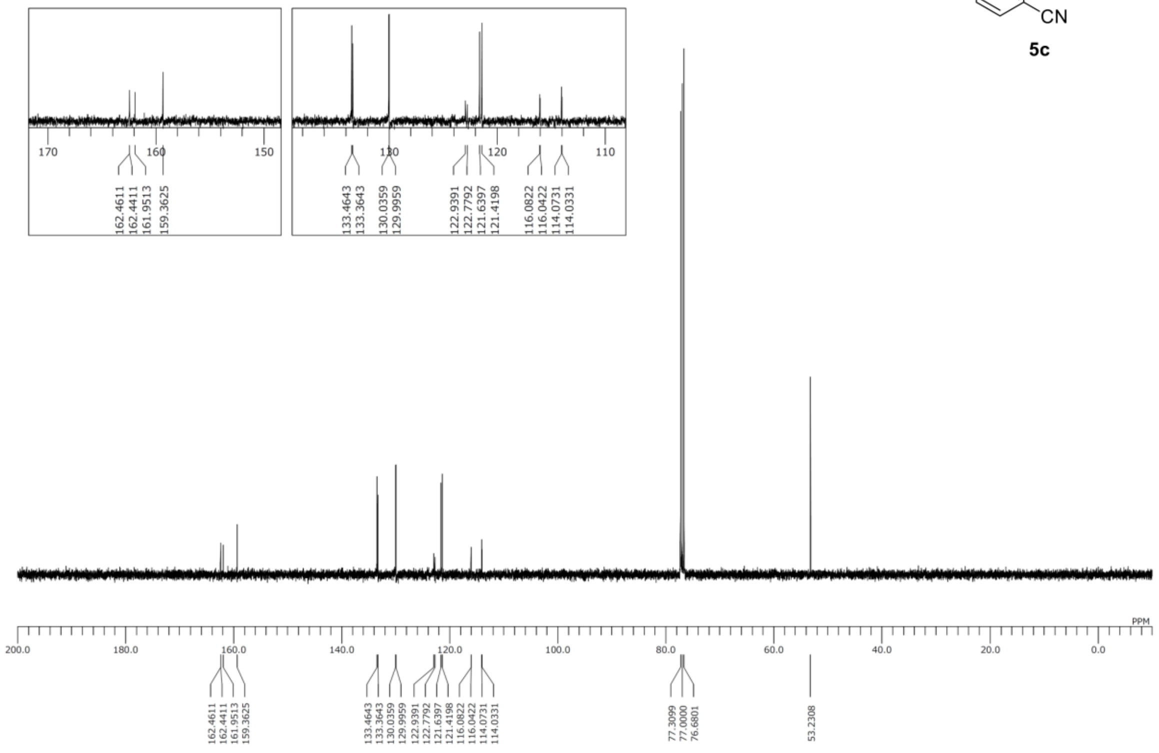

^{19}F NMR spectra of **5c** ($CDCl_3$, 376 MHz)

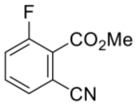

5c

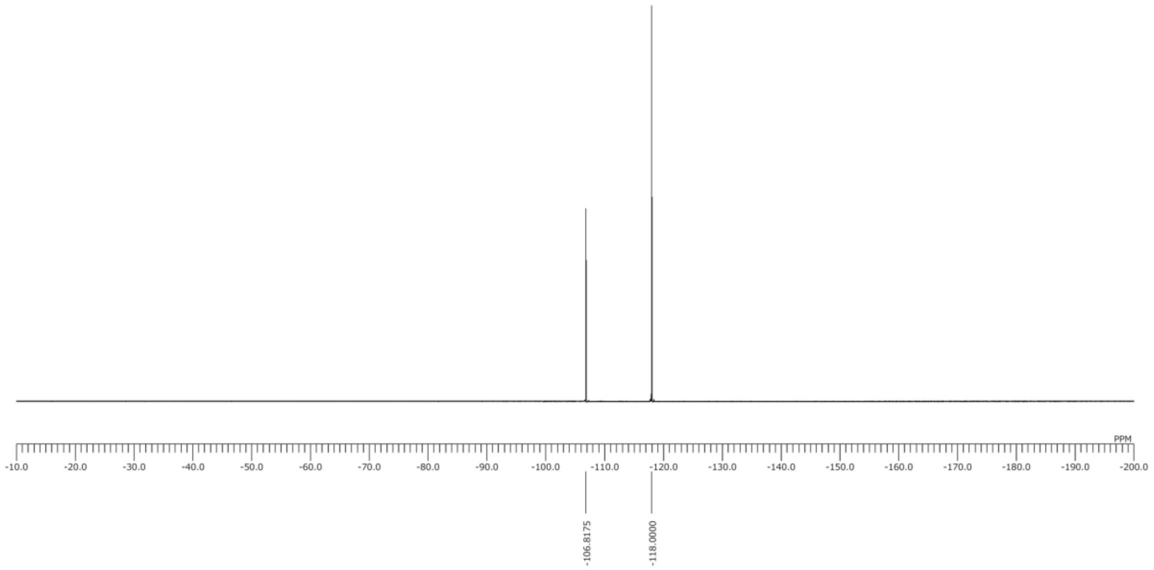

^{1}H NMR spectra of **5d** ($CDCl_3$, 400 MHz)

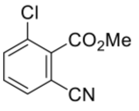

5d

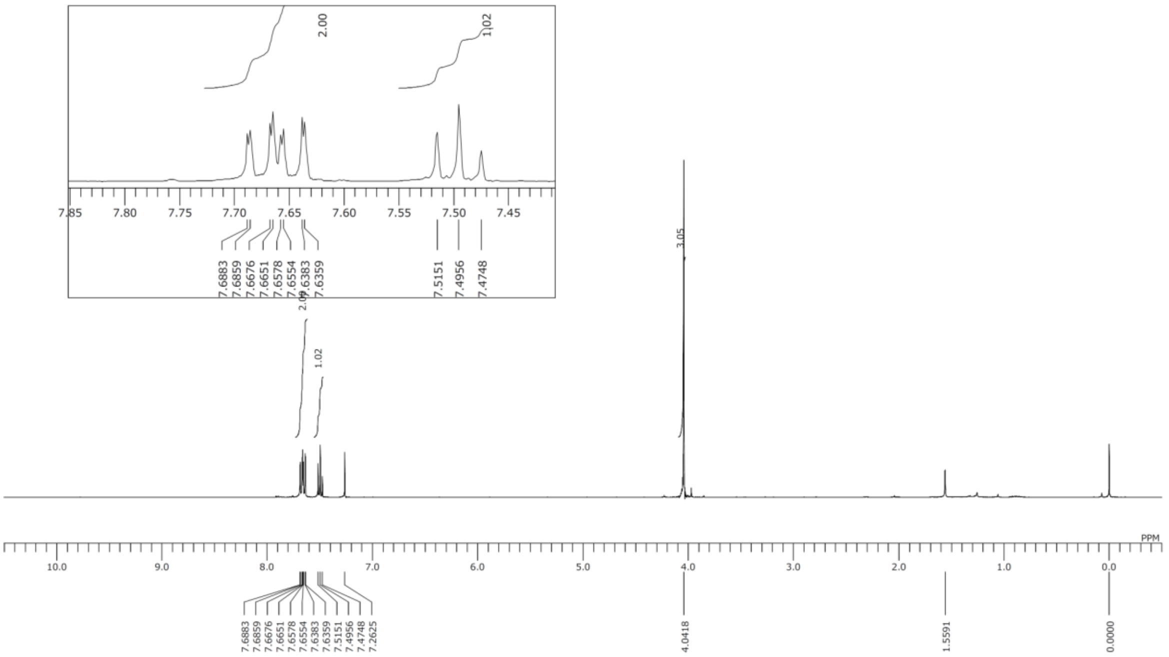

^{13}C NMR spectra of **5d** ($CDCl_3$, 100 MHz)

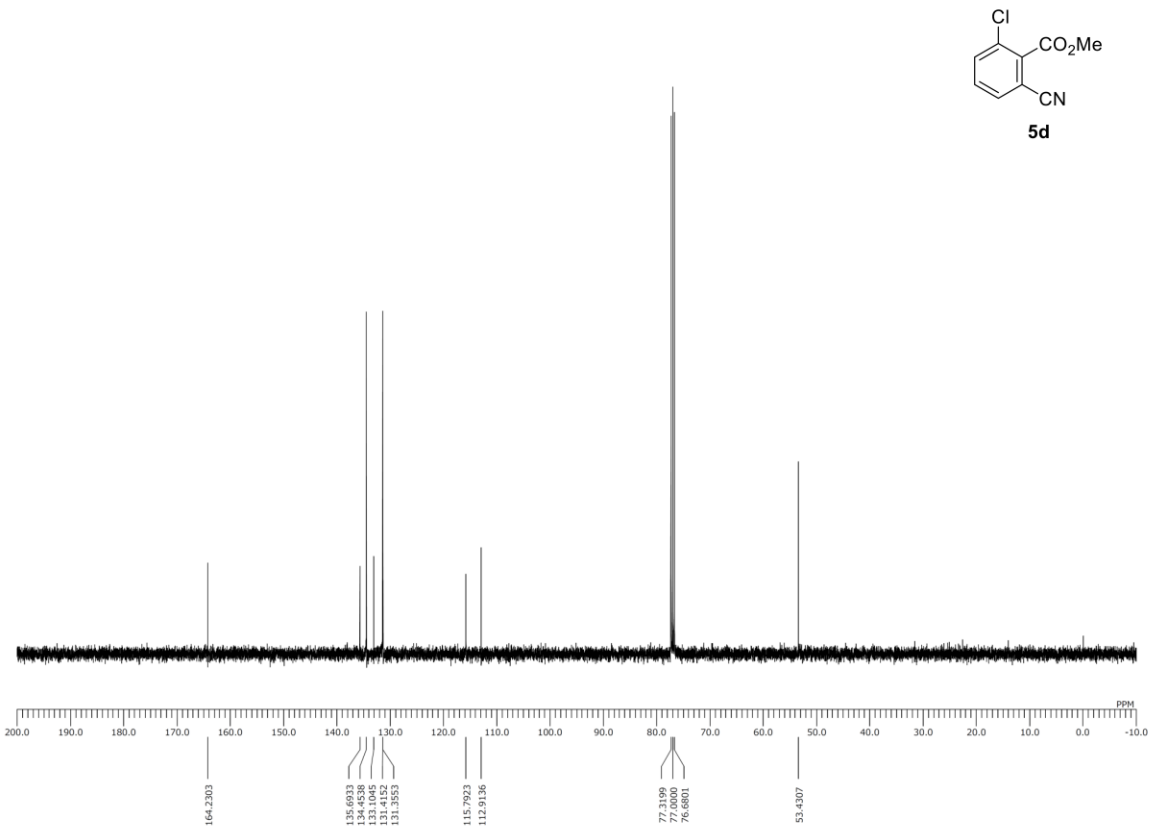

^{1}H NMR spectra of **5e** ($CDCl_3$, 400 MHz)

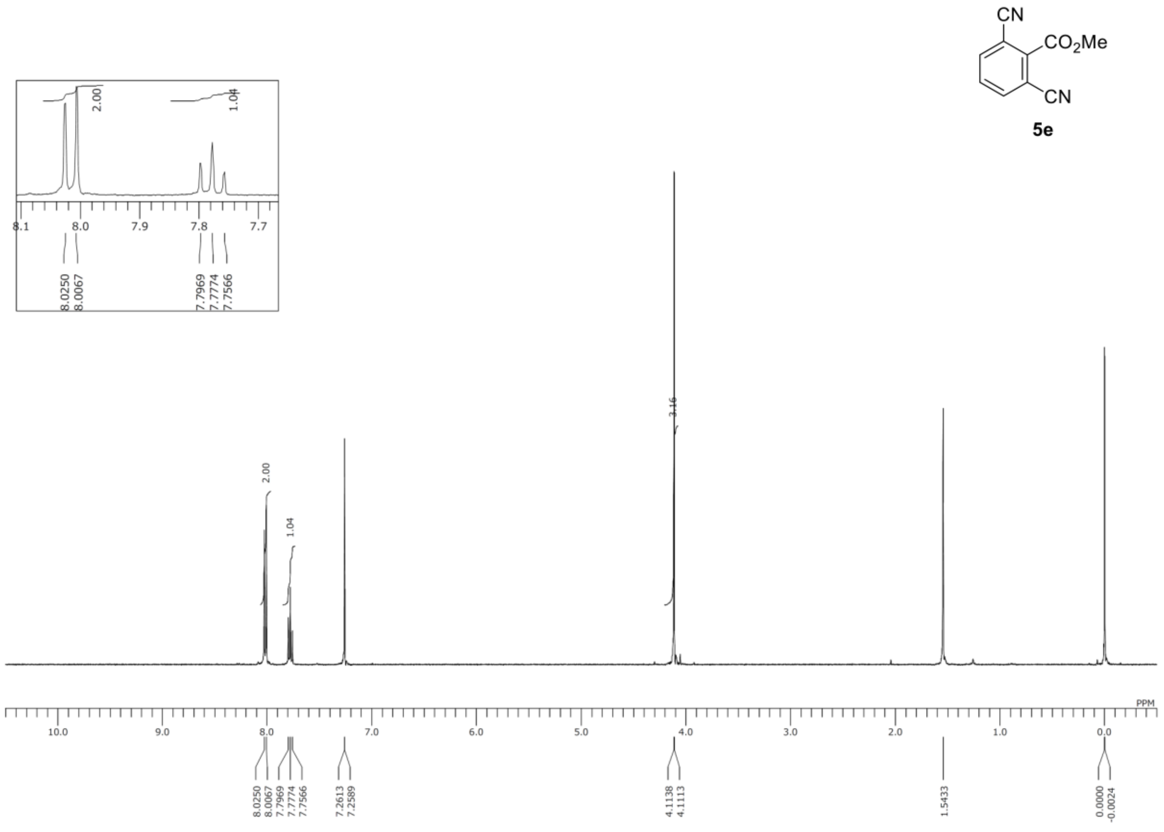

^{13}C NMR spectra of **5e** ($CDCl_3$, 100 MHz)

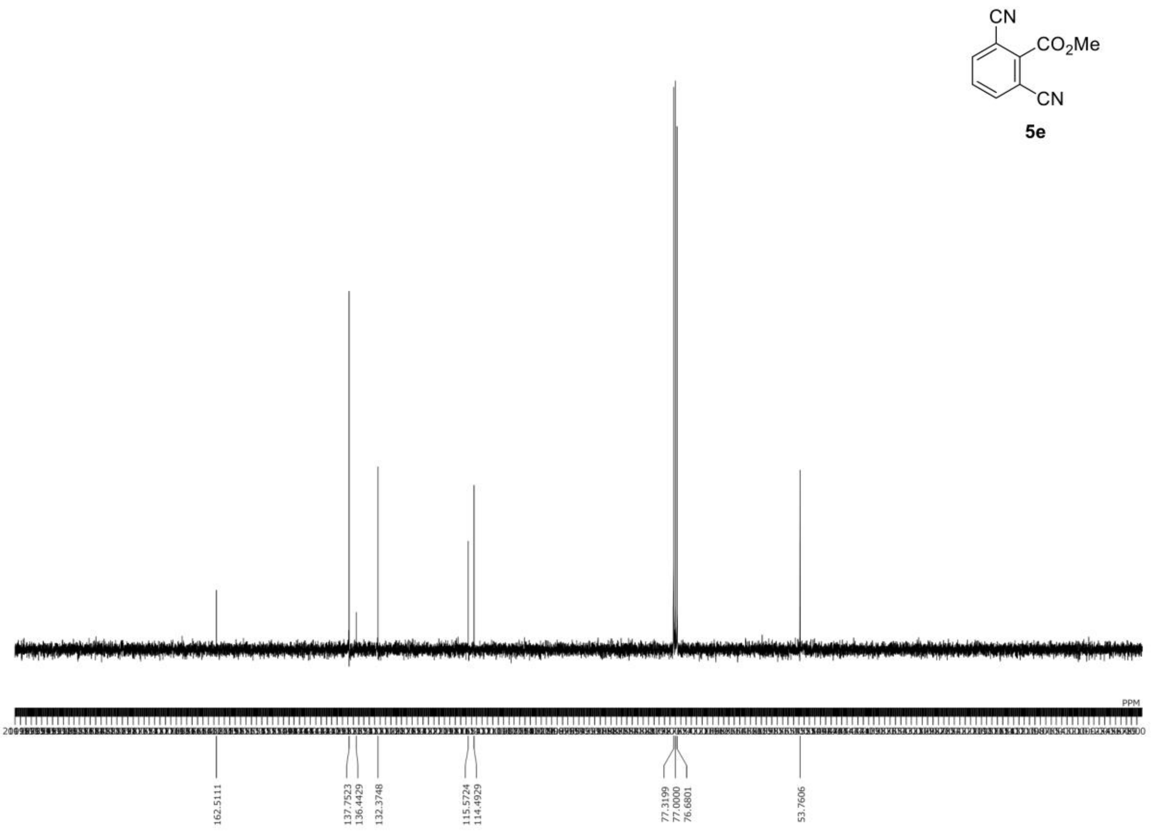

^{1}H NMR spectra of **5f** ($CDCl_3$, 400 MHz)

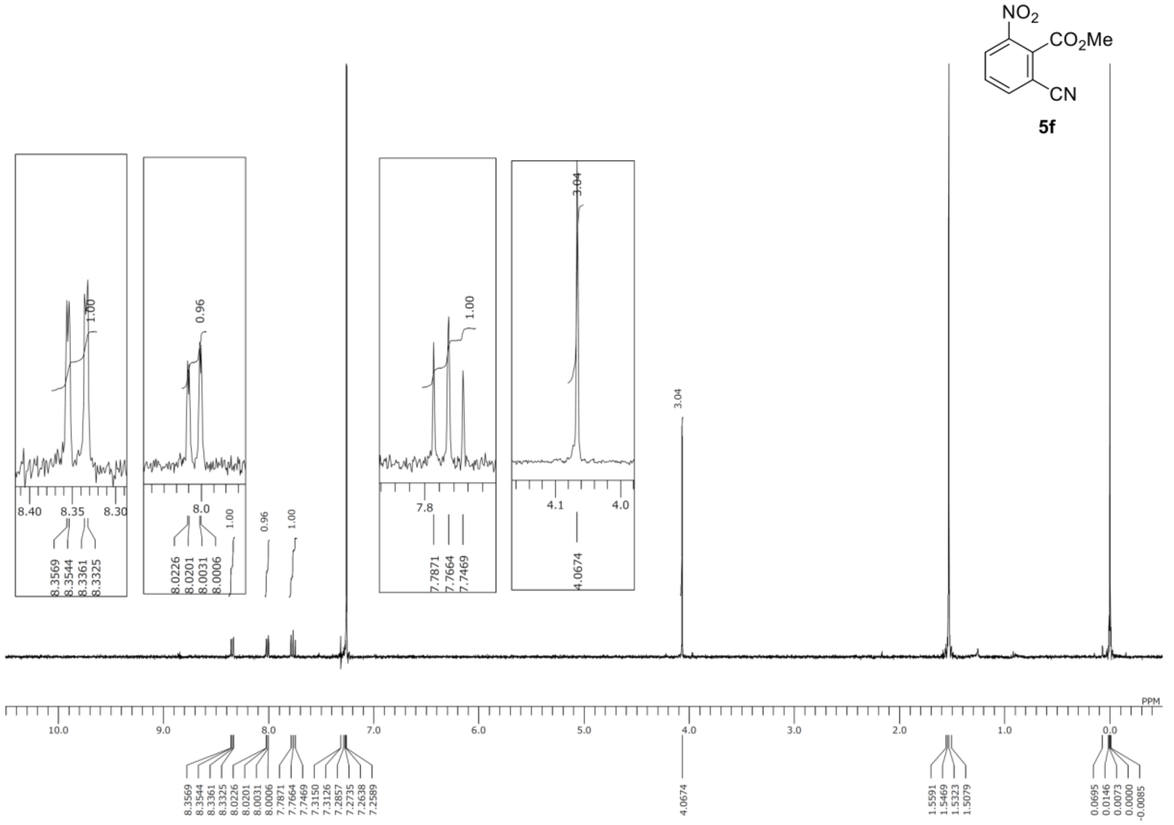

^{13}C NMR spectra of **5f** ($CDCl_3$, 100 MHz)

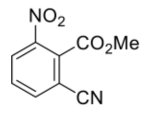

5f

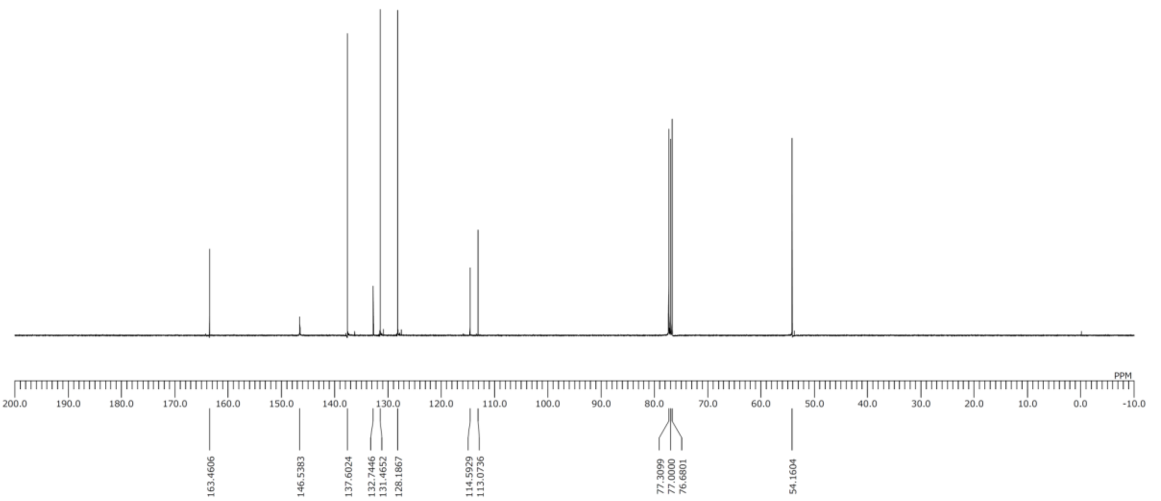

^{1}H NMR spectra of **5g** ($CDCl_3$, 600 MHz)

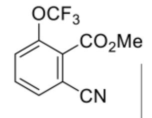

5g

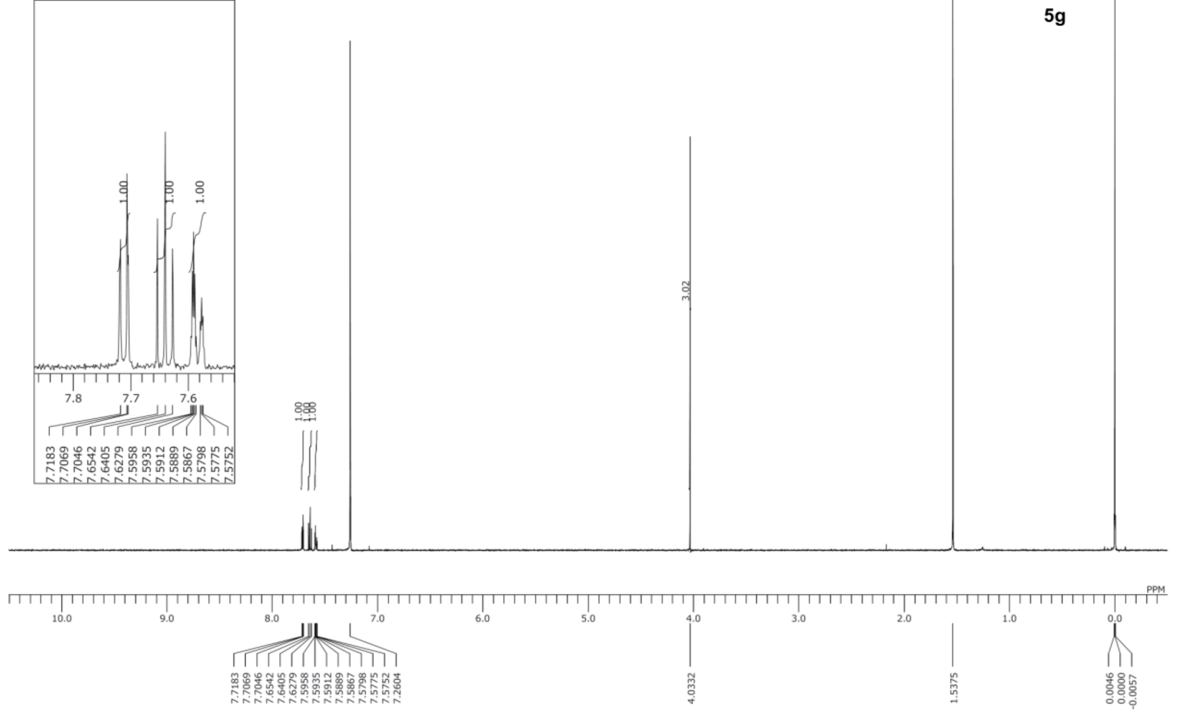

^{13}C NMR spectra of **5g** ($CDCl_3$, 100 MHz)

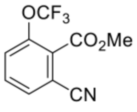

5g

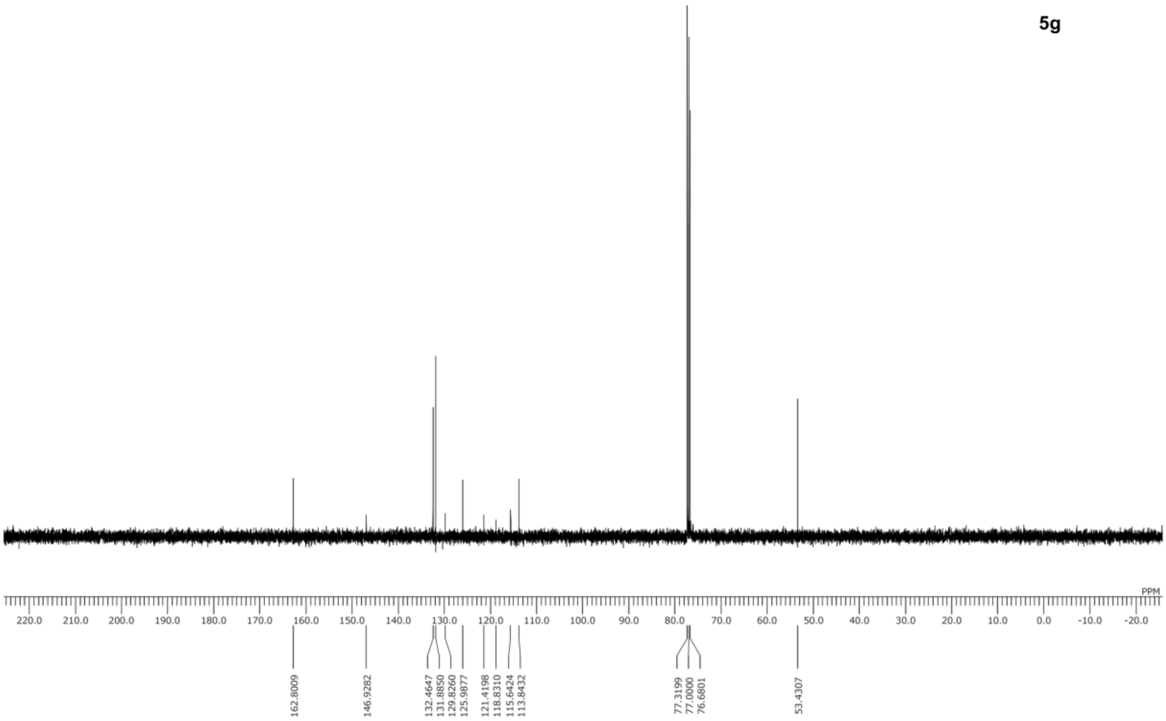

^{19}F NMR spectra of **5g** ($CDCl_3$, 376 MHz)

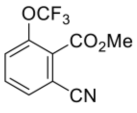

5g

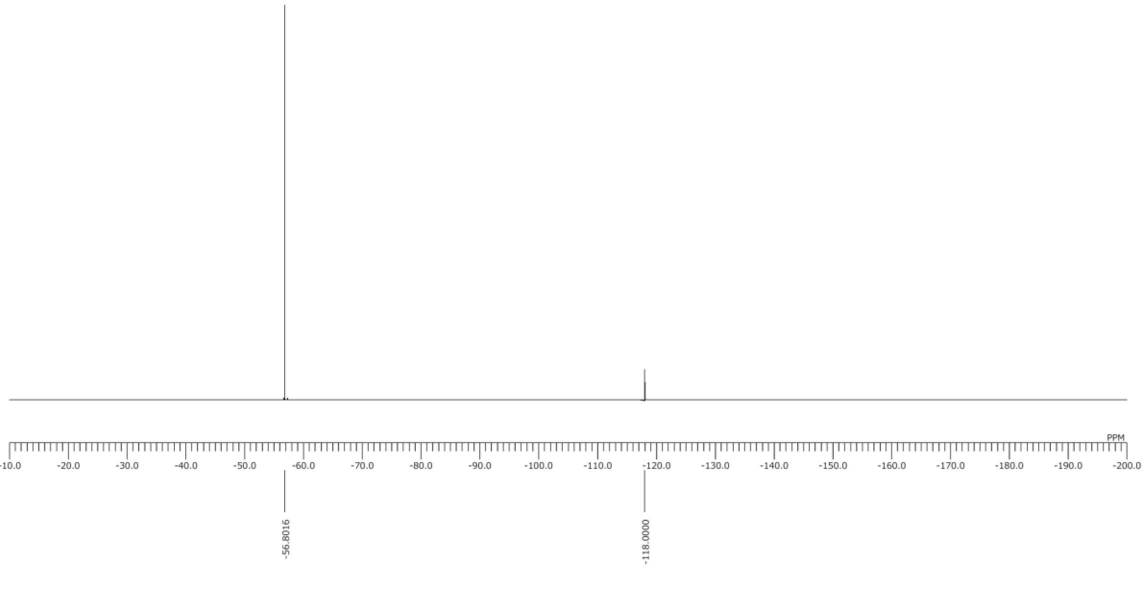

^{1}H NMR spectra of **5h** ($CDCl_3$, 400 MHz)

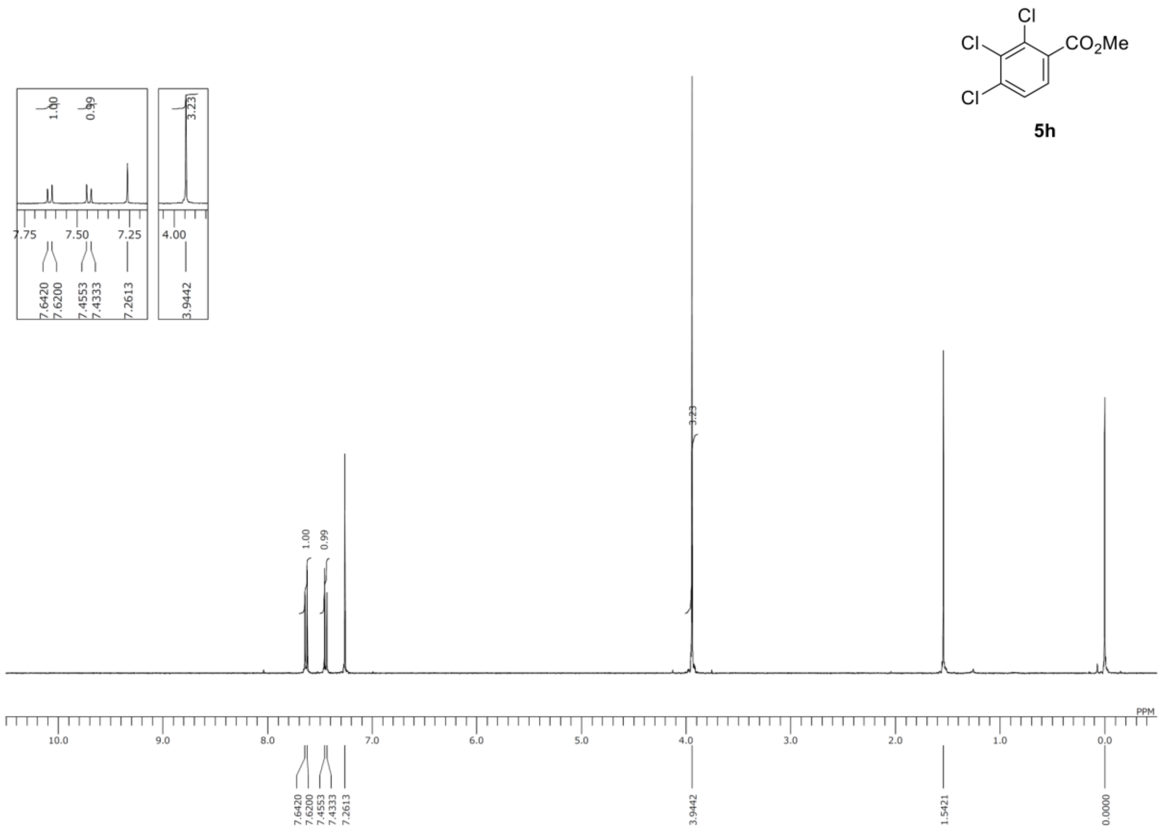

^{13}C NMR spectra of **5h** ($CDCl_3$, 100 MHz)

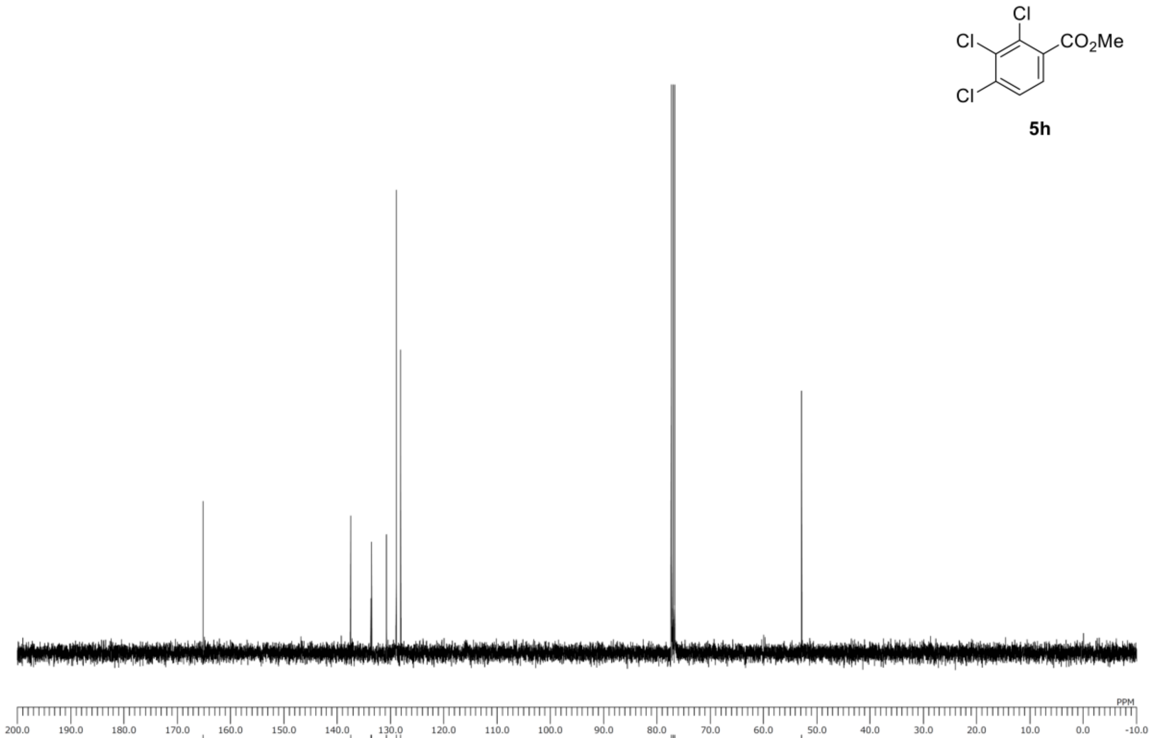

^{1}H NMR spectra of **5i** ($CDCl_3$, 400 MHz)

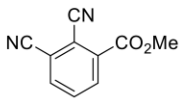

5i

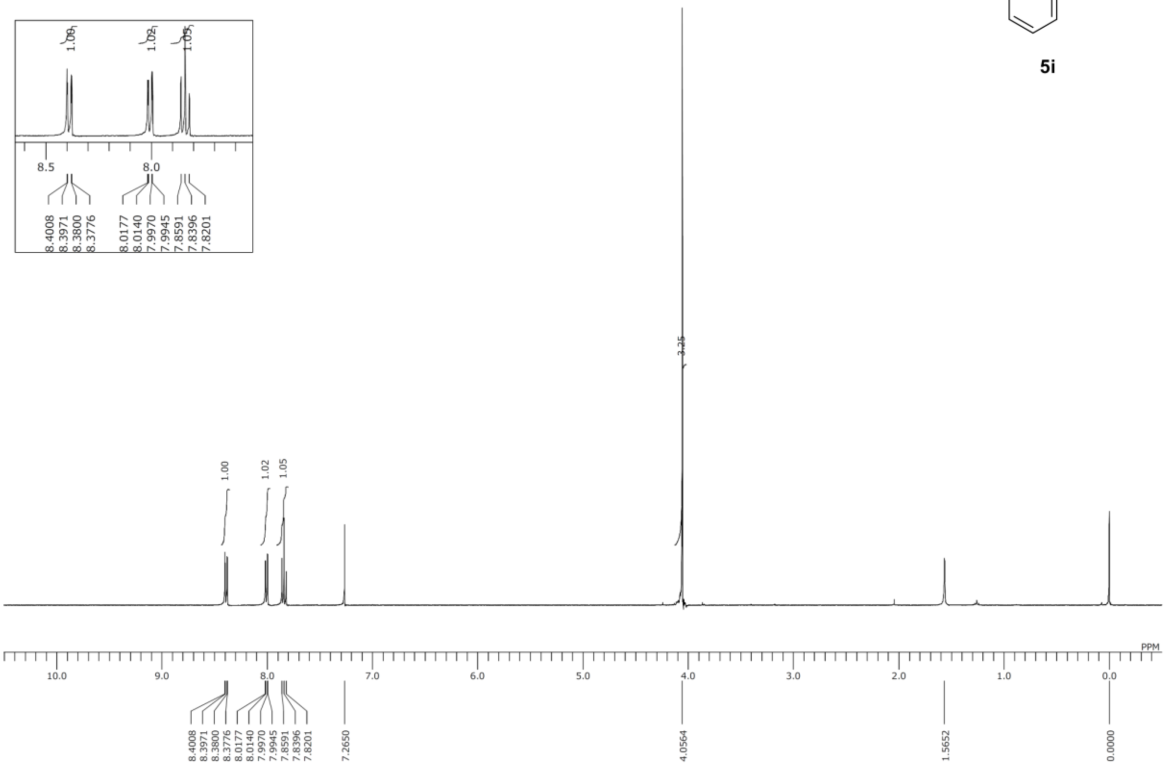

^{13}C NMR spectra of **5i** ($CDCl_3$, 100 MHz)

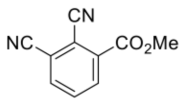

5i

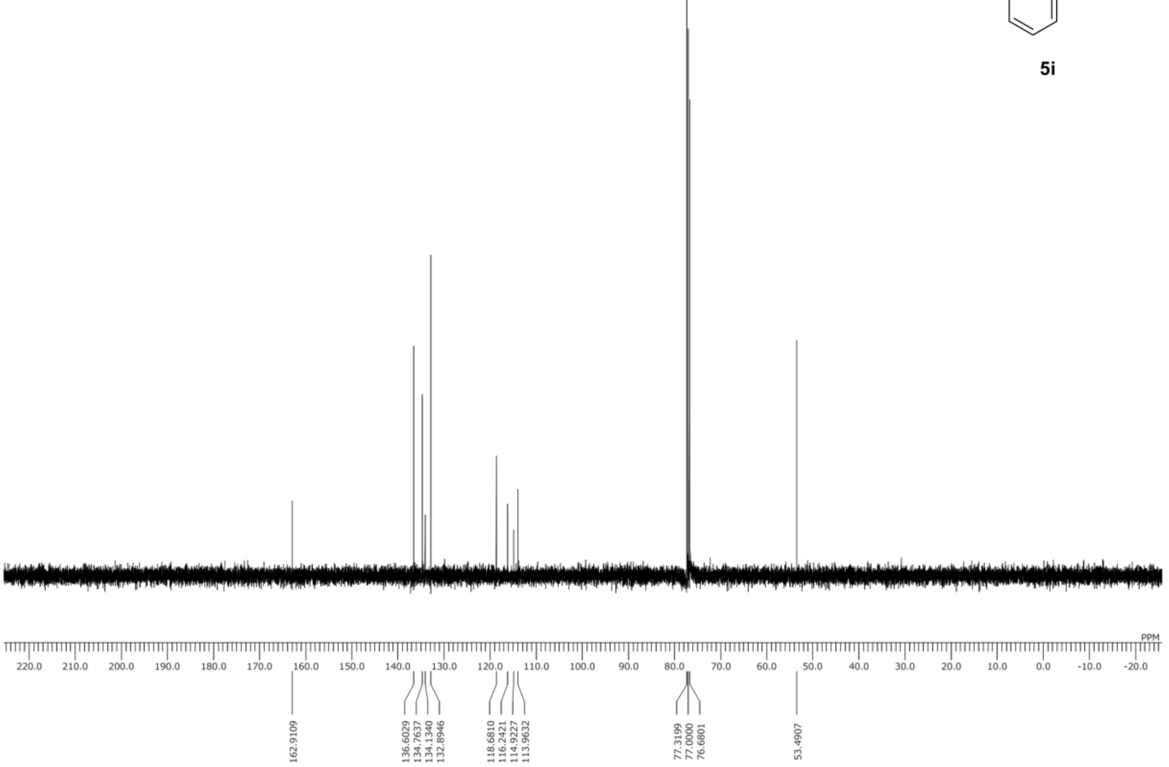

^{1}H NMR spectra of **7** ($CDCl_3$, 400 MHz)

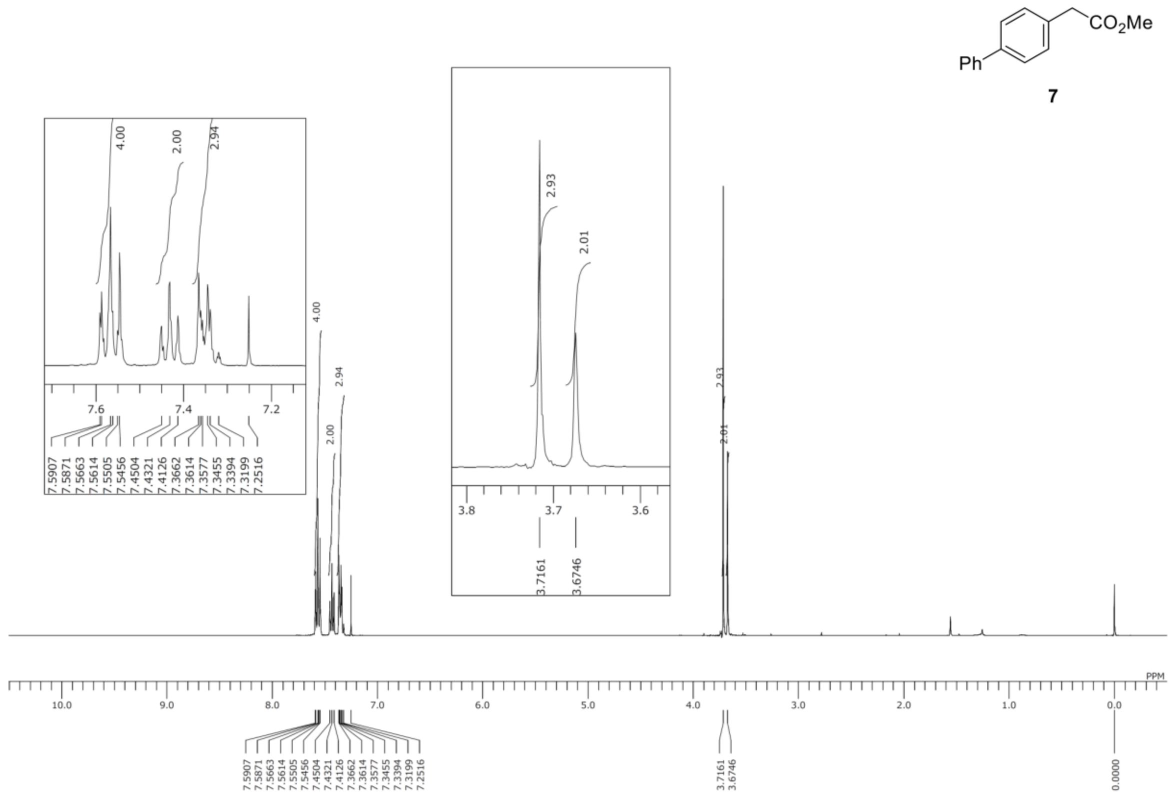

^{13}C NMR spectra of **7** ($CDCl_3$, 150 MHz)

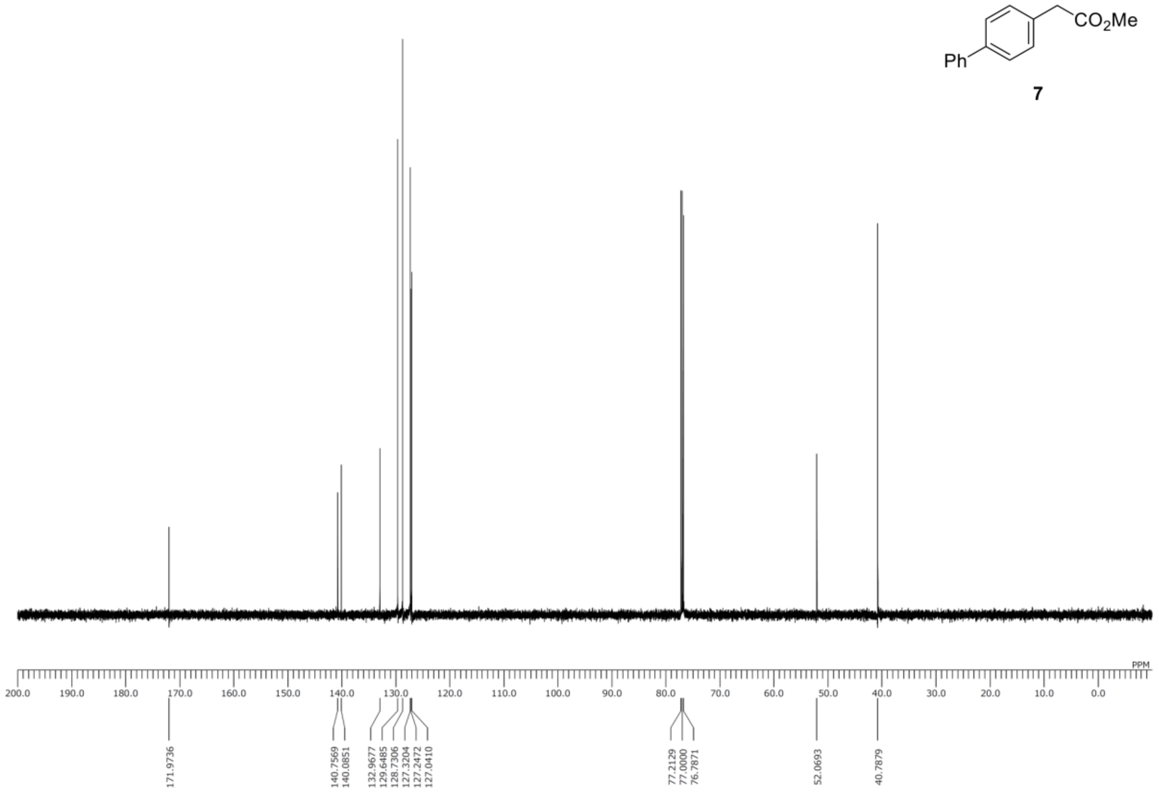

^{1}H NMR spectra of **9** ($CDCl_3$, 400 MHz)

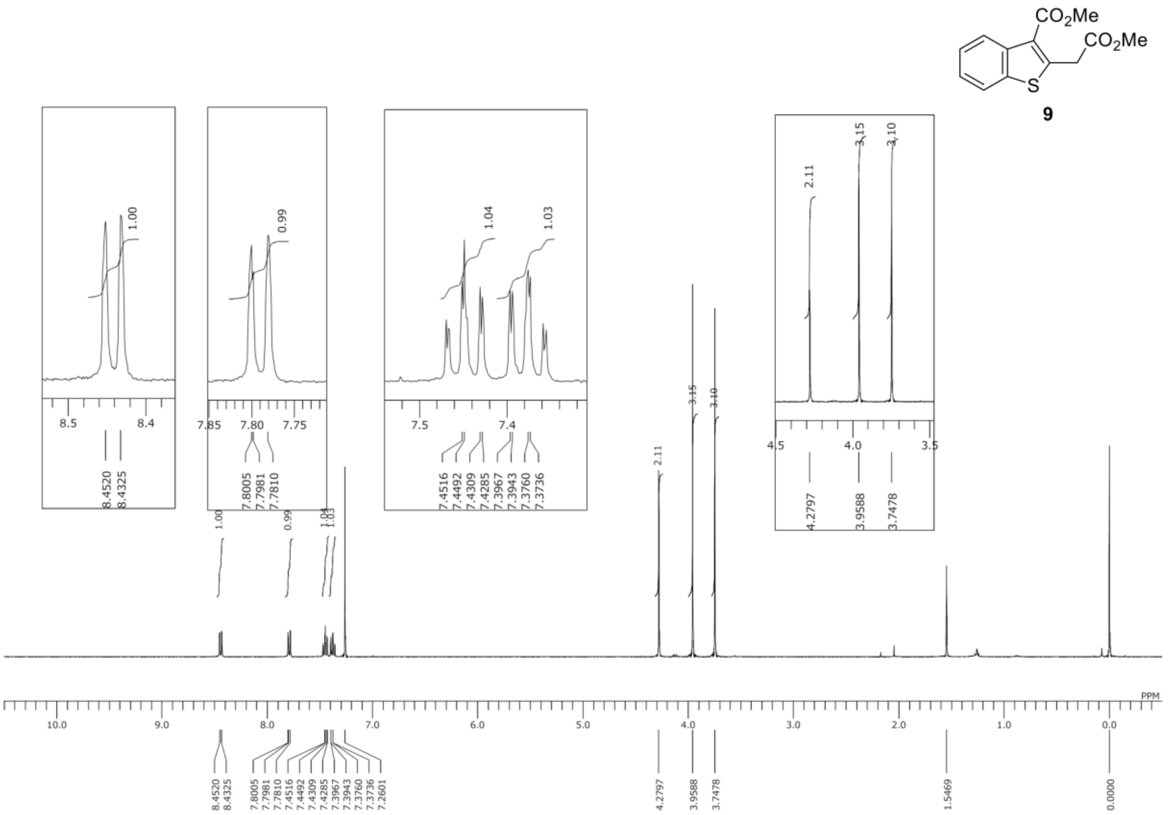

^{13}C NMR spectra of **9** ($CDCl_3$, 150 MHz)

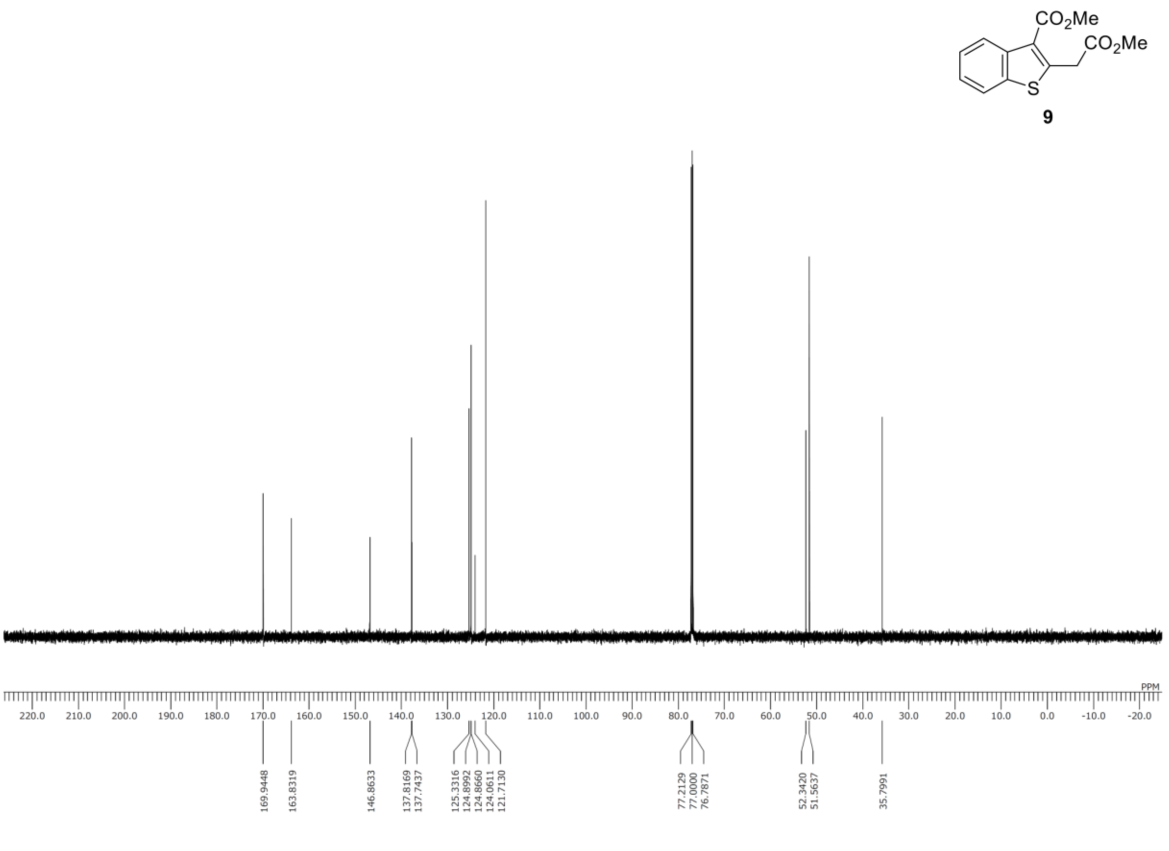

^{1}H NMR spectra of **3a*** ($CDCl_3$, 400 MHz)

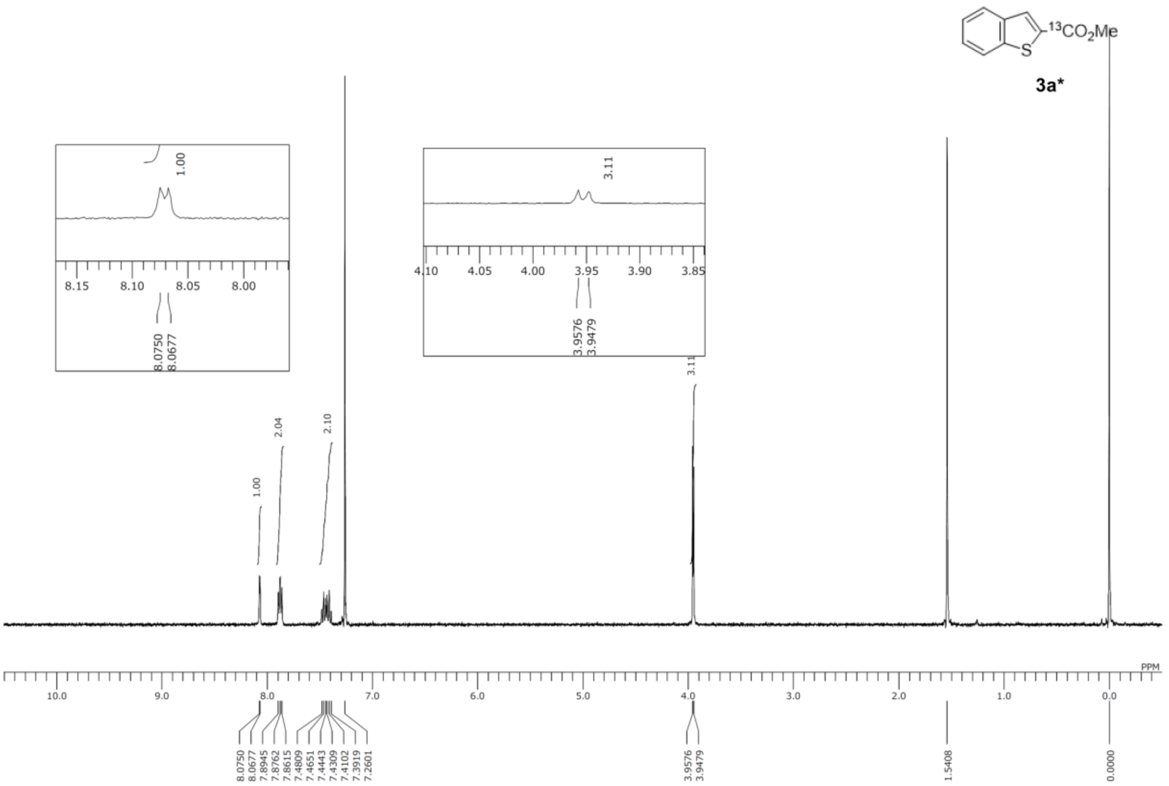

^{13}C NMR spectra of **3a*** ($CDCl_3$, 100 MHz)

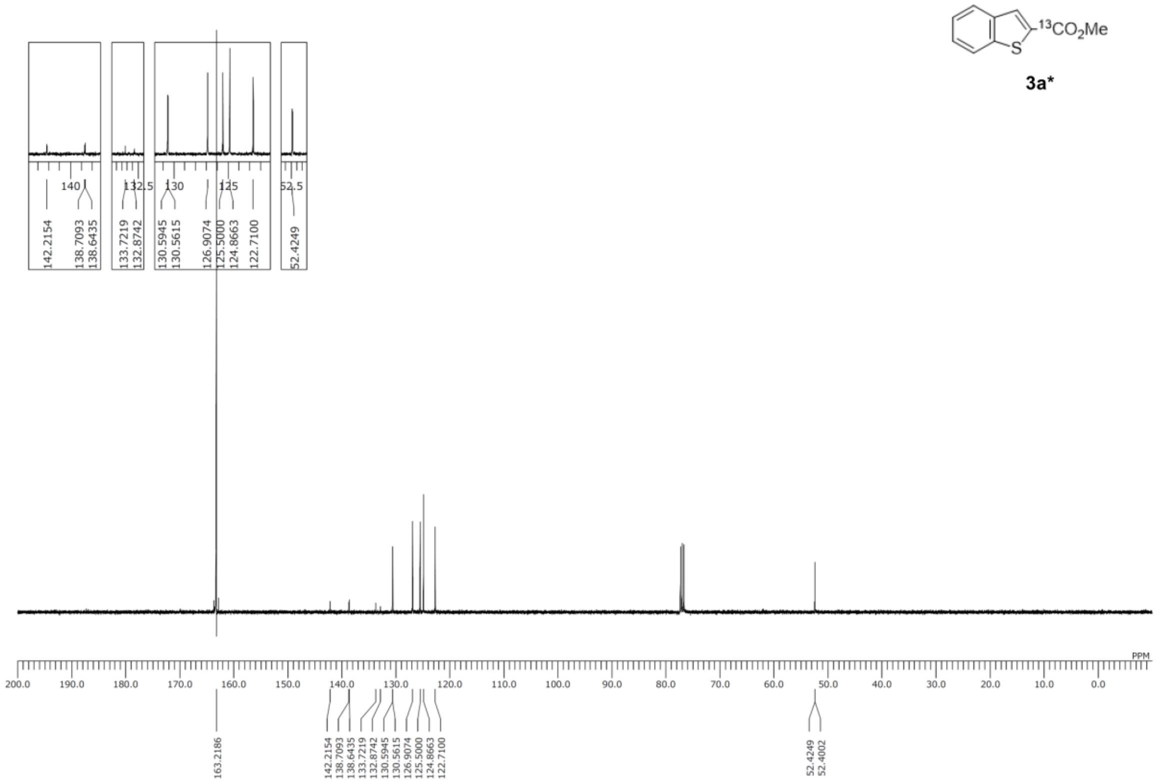

^{1}H NMR spectra of **9*** ($CDCl_3$, 400 MHz)

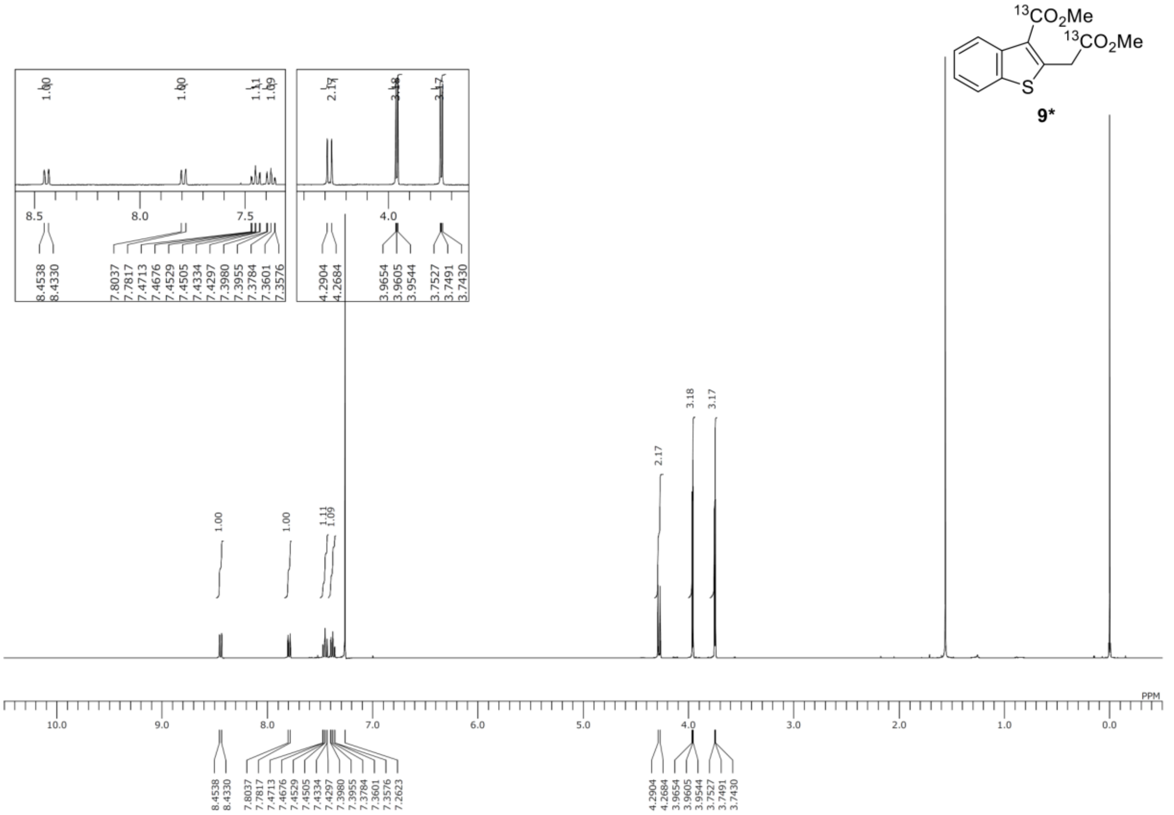

^{13}C NMR spectra of **9*** ($CDCl_3$, 100 MHz)

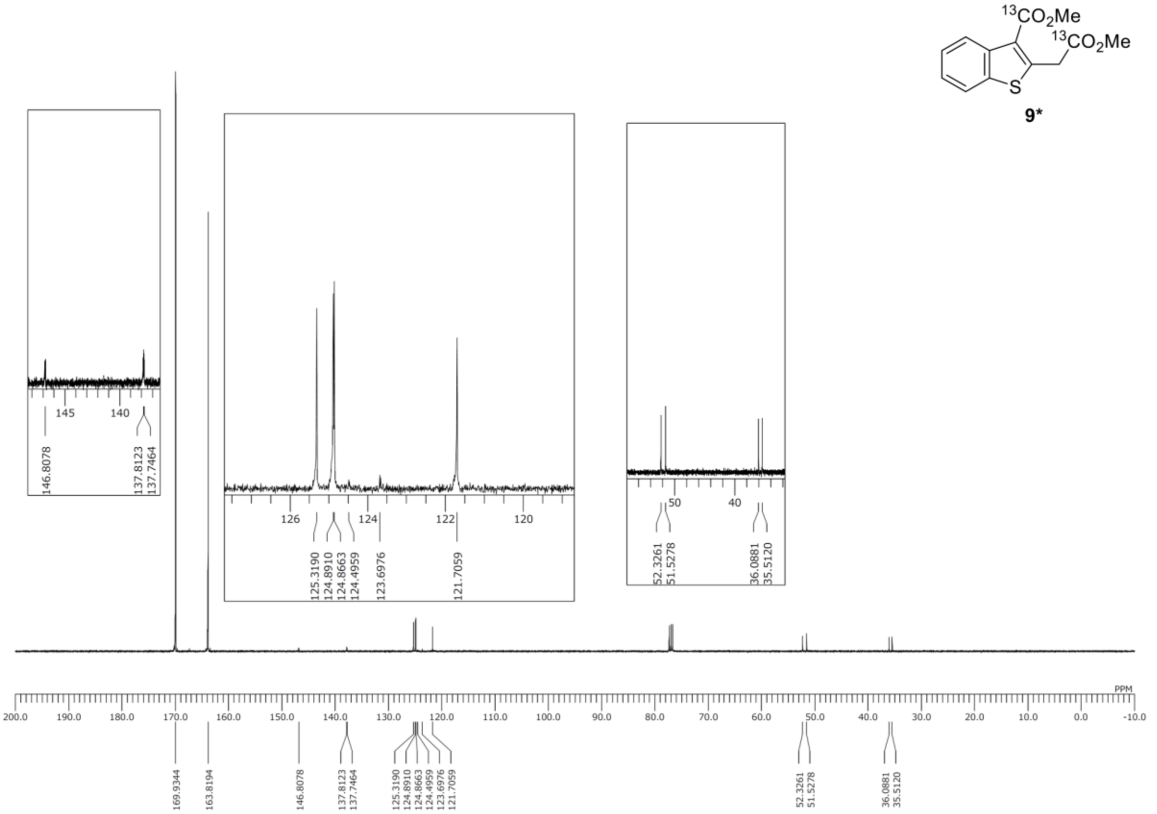

Supplement: Supplementary file 1 — ol4c04388_si_001.pdf [file ol4c04388_si_001.pdf]
